# Evolutionary insights into sequence modifications governing chitin recognition and chitinase inactivity in YKL-40 (HC-gp39, CHI3L1)

**DOI:** 10.1016/j.jbc.2024.107365

**Published:** 2024-05-13

**Authors:** Keita Suzuki, Kazuaki Okawa, Masashi Ohkura, Tomoki Kanaizumi, Takaki Kobayashi, Koro Takahashi, Hiromu Takei, Momo Otsuka, Eri Tabata, Peter O. Bauer, Fumitaka Oyama

**Affiliations:** 1 Department of Chemistry and Life Science, Kogakuin University, Hachioji, Tokyo, Japan; 2 Research Fellow of Japan Society for the Promotion of Science (PD), Chiyoda-ku, Tokyo, Japan; 3 Bioinova a.s., Prague, Czech Republic

**Keywords:** asthma, biomarker, cartilage biology, chitin, chitin-binding, chitinase, enzyme inactivation, inflammation, tumor marker, YKL-40

## Abstract

YKL-40, also known as human cartilage glycoprotein-39 (HC-gp39) or CHI3L1, shares structural similarities with chitotriosidase (CHIT1), an active chitinase, but lacks chitinase activity. Despite being a biomarker for inflammatory disorders and cancer, the reasons for YKL-40's inert chitinase function have remained elusive. This study reveals that the loss of chitinase activity in YKL-40 has risen from multiple sequence modifications influencing its chitin affinity. Contrary to the common belief associating the lack of chitinase activity with amino acid substitutions in the catalytic motif, attempts to activate YKL-40 by creating two amino acid mutations in the catalytic motif (MT-YKL-40) proved ineffective. Subsequent exploration that included creating chimeras of MT-YKL-40 and CHIT1 catalytic domains (CatDs) identified key exons responsible for YKL-40 inactivation. Introducing YKL-40 exons 3, 6, or 8 into CHIT1 CatD resulted in chitinase inactivation. Conversely, incorporating CHIT1 exons 3, 6, and 8 into MT-YKL-40 led to its activation. Our recombinant proteins exhibited properly formed disulfide bonds, affirming a defined structure in active molecules. Biochemical and evolutionary analysis indicated that the reduced chitinase activity of MT-YKL-40 correlates with specific amino acids in exon 3. M61I and T69W substitutions in CHIT1 CatD diminished chitinase activity and increased chitin binding. Conversely, substituting I61 with M and W69 with T in MT-YKL-40 triggered chitinase activity while reducing the chitin-binding activity. Thus, W69 plays a crucial role in a unique subsite within YKL-40. These findings emphasize that YKL-40, though retaining the structural framework of a mammalian chitinase, has evolved to recognize chitin while surrendering chitinase activity.

Chitin is a polymer of *N*-acetyl-D-glucosamine units linked by β-1, 4-bonds and a structural component of crustaceans and insects' exoskeleton, the microfilaria sheath of parasitic nematodes, and fungi cell wall (1, 2, 3). Chitinases hydrolyze the chitin's β-1, 4 glycosidic bonds (1, 3, 4). These enzymes are present in mammals even though they do not synthesize chitin. Two types of chitinases are active in humans and mice: chitotriosidase (Chit1) and acidic chitinase (Chia), respectively (5, 6, 7, 8).

Chitinase-like proteins (CLPs) are similar to chitinases but do not have related enzymatic activity (3, 4, 9, 10). Increased CLP mRNA and/or protein levels have been reported in many inflammatory diseases (1, 4). Mice express BRP-39 (Chi3l1), Ym1 (Chil3), and Ym2 (Chil4), whereas humans express YKL-40 (CHI3L1) and YKL-39 (CHI3L2) (11, 12, 13, 14, 15, 16, 17, 18, 19, 20, 21).

YKL-40 was originally discovered in the culture supernatant of human osteoblastic cells. It was named based on its three N-terminal amino acids, Tyr, Lys, and Leu (YKL), with a molecular weight of 40 kDa (22, 23). This protein was also identified in the culture of human articular chondrocytes and was called human cartilage glycoprotein-39 (HC-gp39) (11). YKL-40 is expressed in osteoarthritic cartilage and osteophytes and has been shown to induce arthritis with pathological changes in bone and cartilage (24, 25). Serum YKL-40 in the rheumatoid arthritis patient group is significantly higher than in other patient groups and healthy controls (26, 27). YKL-40 is also upregulated in asthma, chronic obstructive pulmonary disease, inflammatory bowel disease, alcoholic cirrhosis, Alzheimer’s disease, and cancer (26, 27, 28, 29, 30, 31, 32, 33, 34, 35, 36). Thus, YKL-40 may serve as a biomarker and, once its pathophysiological contribution is well understood, potentially become a therapeutic target in these diseases (3).

YKL-40 plays an important role in protecting against pathogens, antigen-induced and ischemic injury responses, inflammation, and tissue repair (37, 38). In addition, YKL-40 regulates several biological processes, including apoptosis, inflammasome activation, oxidative injury, extracellular matrix regulation, M2 macrophage differentiation, pyrolysis, parenchymal scarring, Th1/Th2 inflammatory balance, transforming growth factor-β1 (TGF-β1) expression, and dendritic cells accumulation. Since many of these processes are mediated *via* interleukin-13 receptor subunit α2 (IL-13Rα2)-dependent mechanisms, YKL-40 has been found to play an essential role in cellular and tissue responses to such insults (39, 40). It has also been shown that YKL-40 interacts with transmembrane protein 219 (TMEM219), galectin 3 (Gal-3), CD44, heparin, and chitin (11, 41, 42, 43, 44, 45, 46, 47). Thus, although the biological functions and mechanisms of action of YKL-40 have been studied, most of its roles and interactions are still not fully elucidated (3).

Mammalian chitinases and CLPs belong to family 18 of glycosyl hydrolases (9, 10, 48, 49). Although the amino acid sequence identity and similarity between YKL-40 and the catalytic domain (CatD) of CHIT1 are 53% and 71%, respectively, YKL-40 lacks chitinase activity. It has generally been believed to be caused by the amino acid substitutions at positions 138 (A) and 140 (L) in the catalytic motif as compared to those in active chitinases (D138 and E140) (4, 9, 10, 20, 50).

Our study aimed to identify the cause of YKL-40's loss of chitinase activity, explore mutations at the catalytic motif and evaluate chimeric proteins composed of YKL-40 and CHIT1 CatD. Contrary to conventional beliefs, our findings indicate that YKL-40 has lost chitin-degrading activity while retaining the ability to bind chitin.

## Results and discussion

### A138D and L140E substitutions in the catalytic motif do not lead to YKL-40 activation

**Figure 1:**
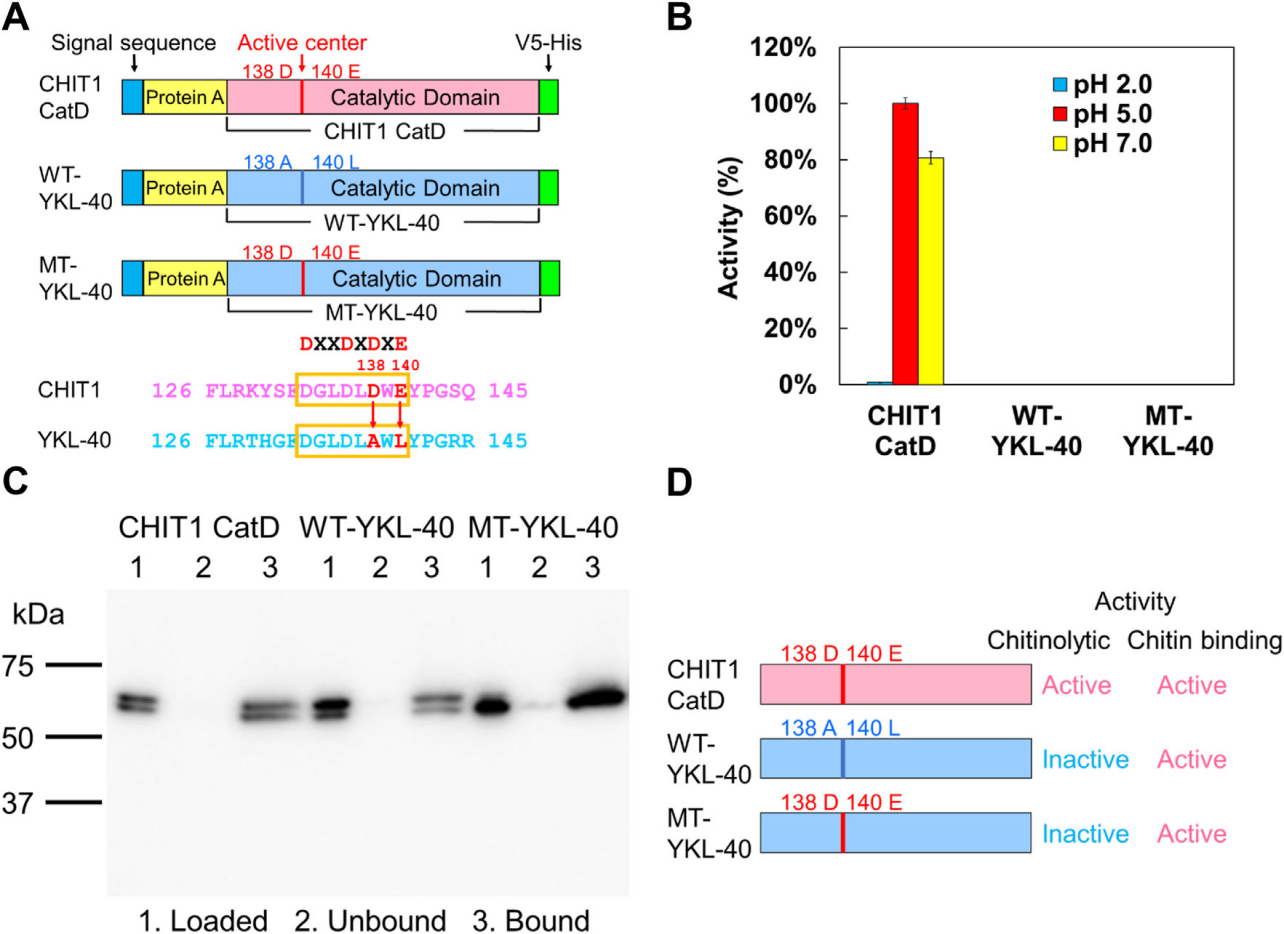
**Introduction of amino acid substitutions of A138D and L140E into YKL-40.***A*, schematic representation of *Escherichia coli*-expressed CHIT1 CatD, WT-YKL-40, and MT-YKL-40 fusion proteins. MT-YKL-40 was constructed by substituting A138D and L140E using WT-YKL-40. The amino acid sequences are color coded: *pink*, CHIT1 CatD; *blue*, YKL-40 sequence. *B*, comparison of the chitinolytic activities of recombinant proteins using the fluorogenic substrate under different pH conditions. Values are represented as relative activity with activity of CHIT1 CatD at pH 5.0 set as 100%. Error bars represent the mean ± SD from a single experiment conducted in triplicate. *C*, binding analysis of recombinant proteins to chitin beads by Western blot. The results were obtained at pH 7.0. Lane 1, loaded sample; lane 2, unbound fraction; and lane 3, bound fraction. *D*, the summary of results presents Figure 1, *B* and *C*. CatD, catalytic domain; CHIT1, chitotriosidase.

The catalytic motif in the family 18 chitinases has a DXXDXDXE sequence (Fig. 1*A*) (9, 10, 48, 49). It has been generally believed that amino acid mutations at positions 138 and 140 within this motif define the chitinase activity of YKL-40. Therefore, we prepared a mutant form of YKL-40 (MT-YKL-40) by introducing amino acid substitutions A138D and L140E. Human CHIT1 CatD, WT-YKL-40, and MT-YKL-40 were expressed as fusion proteins containing *Staphylococcus aureus* Protein A and V5-His tags using *Escherichia coli* as described in the Experimental procedures (Figs. 1*A* and S1) (51). To compare the chitinase activities of these recombinant proteins, fluorogenic substrate 4-methylumbelliferyl *N, N′*-diacetyl-β-D-chitobioside [4-MU-(GlcNAc)_2_] was used at different reaction pH (51, 52).

CHIT1 CatD had high activity at pH 5.0 and 7.0 while low at pH 2.0 (53) (Figs. 1*B*; S2 and Table S1). In contrast, WT- and MT-YKL-40 showed no chitinolytic activity at any of the pH conditions. Next, we aimed to evaluate whether the lack of substrate degradation could be caused by the inability of our recombinant YKL-40 proteins to interact with chitin as observed in endogenous YKL-40 (46) and found that both WT- and MT-YKL-40 bind to chitin (Fig. 1*C*). This suggests that the lack of chitinase activity in YKL-40 is not merely due to simple amino acid substitutions in the conserved catalytic motif and indicating the presence of other causative regions involved in the inactivation of YKL-40 (Fig. 1*D*).

### Changes in multiple regions of the molecule cause loss of YKL-40 chitinase activity

**Figure 2:**
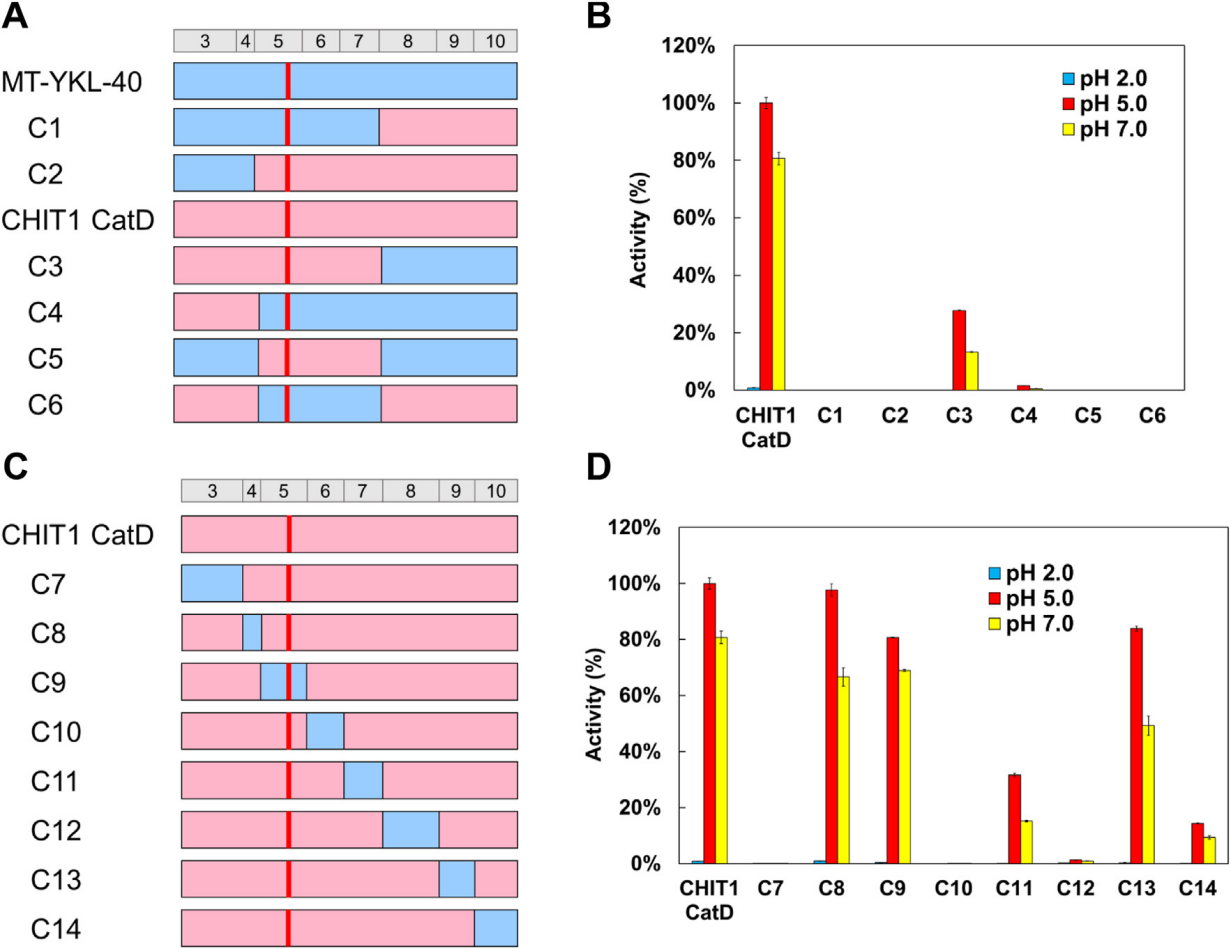
**Identifying the cause of MT-YKL-40 inactivation using chimeric proteins.***A*, schematic representation of MT-YKL-40 and WT-CHIT1 CatD chimeric proteins (C1-C6). The size of each exon is shown above the schematic diagram of the chimera. The amino acid sequences are color coded: *pink*, CHIT1 CatD; *blue*, YKL-40 sequence. *B*, the chitinolytic activities of MT-YKL-40-CHIT1 CatD chimeric proteins (C1-C6), MT-YKL-40, and CHIT1 CatD under different pH conditions. Error bars represent the mean ± SD from a single experiment conducted in triplicate. *C*, schematic representation of MT-YKL-40-CHIT1 CatD chimeras (C7-C14). *D*, comparison of the chitin-degrading activity of C7-C14. Error bars represent the mean ± SD from a single experiment conducted in triplicate. CatD, catalytic domain; CHIT1, chitotriosidase.

To determine the region responsible for YKL-40 inactivation, several chimeric proteins based on MT-YKL-40 (inactive) and CHIT1 CatD (active) were constructed (Figs. 2*A* and S3). Chimera C1, where CHIT1 CatD exons 8 to 10 were introduced into MT-YKL-40, was completely inactive (Figs. 2*B*; S2 and S3 and Table S1). Chimera C2, CHIT1 CatD with YKL-40 exons 3 and 4 also showed no activity. In contrast, chimera C3, having CHIT1 CatD exons 3 to 7 and YKL-40 exons 8 to 10, had chitinase activity, although lower than Chit1 CatD (28% of CHIT1 CatD at pH 5.0, 13% at pH 7.0). In chimera C4, MT-YKL-40 with CHIT1 CatD exons 3 and 4 exhibited a very low but detectable chitinolytic activity (1.5% CHIT1 CatD at pH 5.0, 0.4% at pH 7.0). These data indicate that exons 3 and 4 are involved in the YKL-40 inactivation. At the same time, changes in exons 5 to 10 also contribute to the loss of chitinolytic activity.

To elucidate the involvement of these exons, we constructed chimeras C5 and C6, where CHIT1 CatD exons 5 to 7 were introduced to MT-YKL-40, and MT-YKL-40 exons 5 to 7 were introduced to CHIT1 CatD, respectively (Figs. 2*A* and S3). Chimera C5 remained inactive suggesting that exons 5 to 7 only partly control the protein activity (Figs. 2*B*; S2 and S3 and Table S1). On the other hand, in C6, MT-YKL-40 exons 5 to 7 completely inactivated CHIT1 CatD. These results suggest that the causative region that inactivates chitinase activity is present throughout the YKL-40 molecule.

### Contribution of YKL-40 exons to CHIT1 activity suppression

To identify the exons with major inactivating effects, we prepared chimeras C7-C14 with individually replaced CHIT1 CatD exons with the YKL-40 sequences (Figs. 2*C* and S3). Presence of YKL-40 exons 3 (C7), 6 (C10), and 8 (C12) led to an almost complete loss of chitinolytic activity (less than 1.5% of the CHIT1 CatD activity at pH 5.0) (Figs. 2*D*; S2 and S3 and Table S1). Substitution of exons 7 (C11) and 10 (C14) resulted in a major activity reduction by more than 32% and 14% at pH 5.0, respectively. Chimeras C8, C9, and C13, where exons 4, 5, and 9 of CHIT1 CatD were replaced with YKL-40, respectively, retained their activity, albeit with some decrease. Next, we examined the affinity of these proteins to chitin. All chimeras were able to bind the substrate (Fig. S4). These results indicate that introducing exons 3, 6, or 8 of YKL-40 into CHIT1 led to inactivation, although these chimeric proteins maintained the chitin-binding ability.

### MT-YKL-40 activation by exons replacement

**Figure 3:**
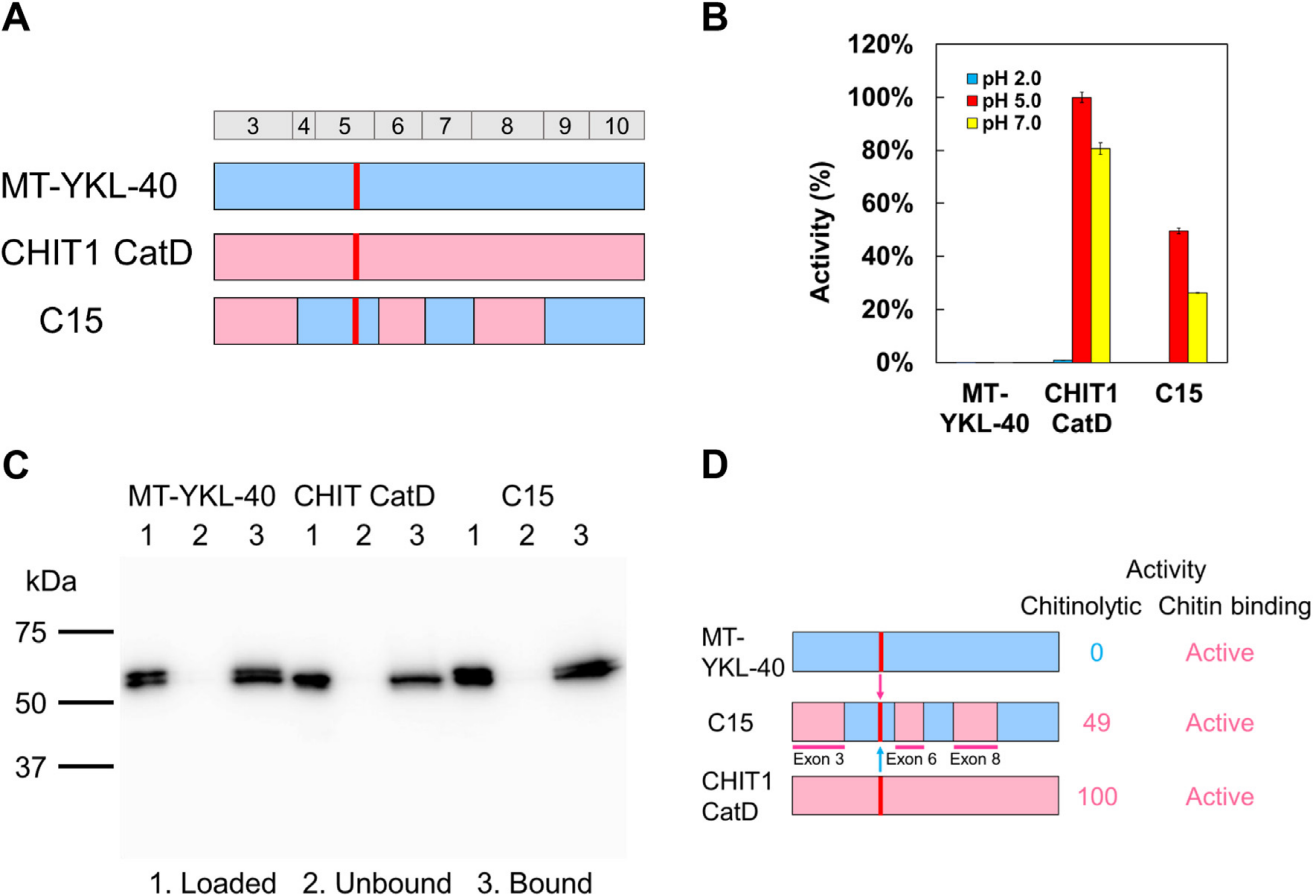
**Activation of YKL-40.***A*, schematic representation of MT-YKL-40-CHIT1 CatD chimera (C15). *B*, comparison of the chitin-degrading activity of C15 with MT-YKL-40 and CHIT1 CatD. Error bars represent the mean ± SD from a single experiment conducted in triplicate. *C*, binding analysis of recombinant proteins to chitin beads by Western blot. The results were obtained under pH 7.0 conditions. Lane 1, loaded sample; lane 2, unbound fraction; lane 3, bound fraction. *D*, summary of results presents Figure 3, *B* and *C*. CatD, catalytic domain; CHIT1, chitotriosidase.

To activate MT-YKL-40, we replaced MT-YKL-40 exons 3, 6, and 8 with those of CHIT1 CatD (chimera C15) (Figs. 3*A* and S3). The activity of C15 was about 49% and 33% of that of CHIT1 CatD at pH 5.0 and 7.0, respectively (Figs. 3*B*; S2 and S3 and Table S1). As expected, C15 interacted with chitin (Fig. 3*C*).

The activation of MT-YKL-40 by about a half of that of CHIT1 CatD by replacing three exons indicates that this protein cannot be fully activated by exchanging only some parts of the sequence (Fig. 3*D*).

### Assessment of folding in recombinant and chimeric proteins for chitinase and chitin-binding

**Figure 4:**
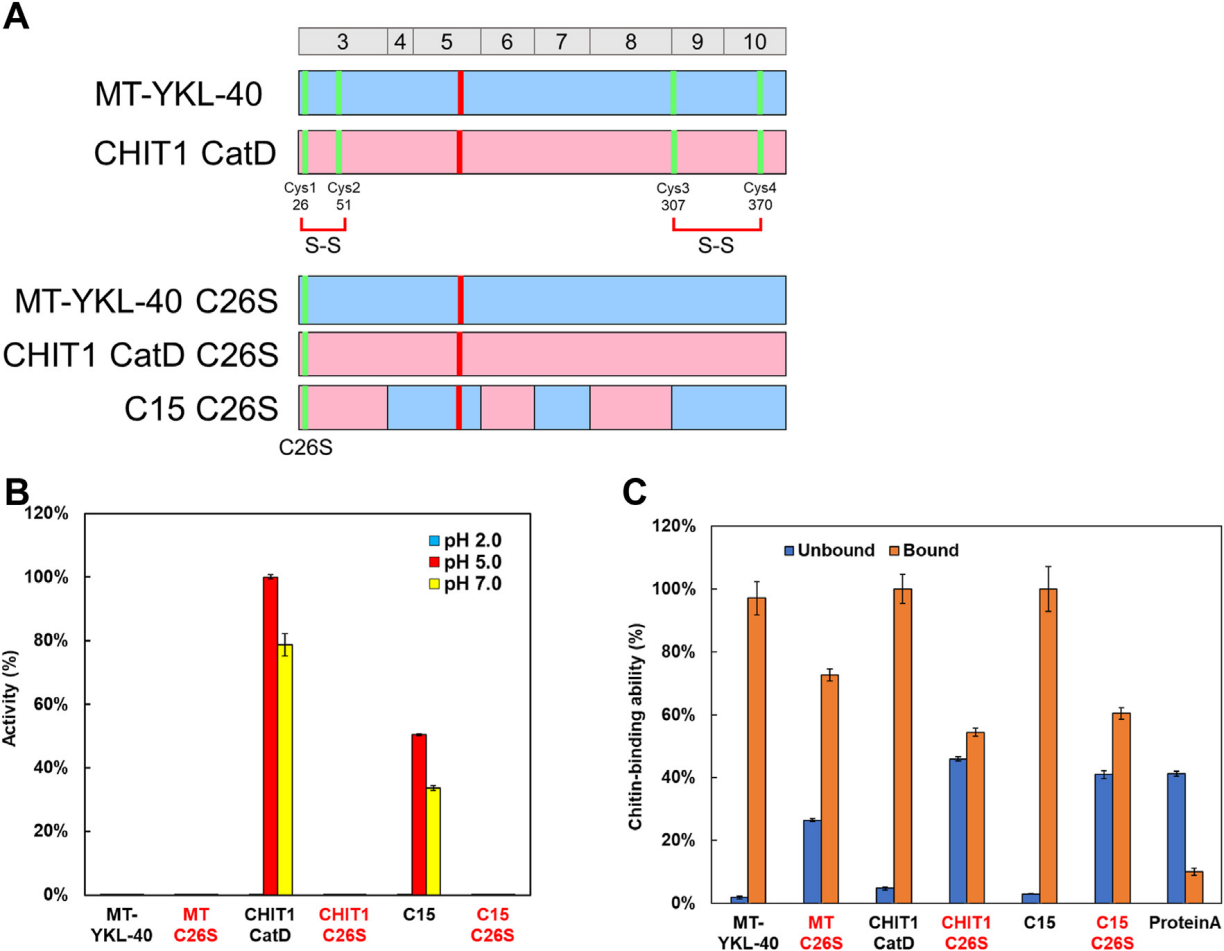
**Confirmation of the folding of recombinant proteins.***A*, schematic representation of MT-YKL-40, CHIT1 CatD, and chimera C15 with and without C26S mutation. Amino acid sequences are color coded: *pink* for CHIT1 CatD and *blue* for YKL-40. Two conserved disulfide bonds at the N- and C-terminals of YKL-40, shared with CHIT1 and CHIA. We substituted Cys26 by Ser, implicated in N-terminal disulfide bond formation, in MT-YKL-40, CHIT1 CatD, and chimera C15. *B*, the chitinolytic activity of MT-YKL-40, CHIT1 CatD, and C15 with C26S mutation. Error bars represent mean ± SD from a single experiment conducted in triplicate. *C*, effect of amino acid substitution on chitin-binding activity. Protein A exhibited minimal chitin binding and was considered as background. Error bars represent mean ± SD from a single experiment conducted in triplicate. CatD, catalytic domain; CHIA, acidic chitinase; CHIT1, chitotriosidase.

Next, we focused on the folding and disulfide bond formation in MT-YKL-40, CHIT1 CatD, and C15. Previous studies and X-ray crystallography results have shown that YKL-40 possesses two conserved disulfide bonds at the N terminals and C terminals, a feature shared with CHIT1 and CHIA (54, 55, 56, 57) (Fig. 4*A*). The critical role of the N-terminal disulfide bond is highlighted by the fact that WT lycaon Chia, with Ser at position 26, displayed no chitinase activity, whereas introducing Cys at this position resulted in chitinase activity (51). This observation emphasizes the importance of the N-terminal disulfide bond, which is preserved in the N-terminal regions of YKL-40 and CHIT1 CatD.

To confirm these observations, we replaced Cys at position 26 with Ser in the tested molecules (Figs. 4*A* and S3). As expected, MT-YKL-40 exhibited no activity regardless of the amino acid at position 26, while CHIT1 CatD and C15 lost their activity upon the Cys to Ser (C26S) mutation (Figs. 4*B*; S2 and S3 and Table S1).

We also examined the influence of the C26S substitution on chitin-binding activity and it decreased chitin-binding activity by 40% to 50%, resulting in an increased pass-through fraction in the chitin-binding experiment (Fig. 4*C*). This suggests that replacing Cys at position 26 leads to a change in the tertiary structure, reducing the affinity to chitin and the loss of chitinase activity.

Our experiments indicate that the recombinant proteins exhibit properly formed disulfide bonds, confirming the presence of a well-defined structure in molecules with chitinase and chitin-binding activity. This provides indirect evidence of correct folding in our recombinant proteins, as supported by the observed changes in chitinase and chitin-binding activity.

### Identifying the key amino acids for chitinase inactivation while maintaining chitin-binding activity

**Figure 5:**
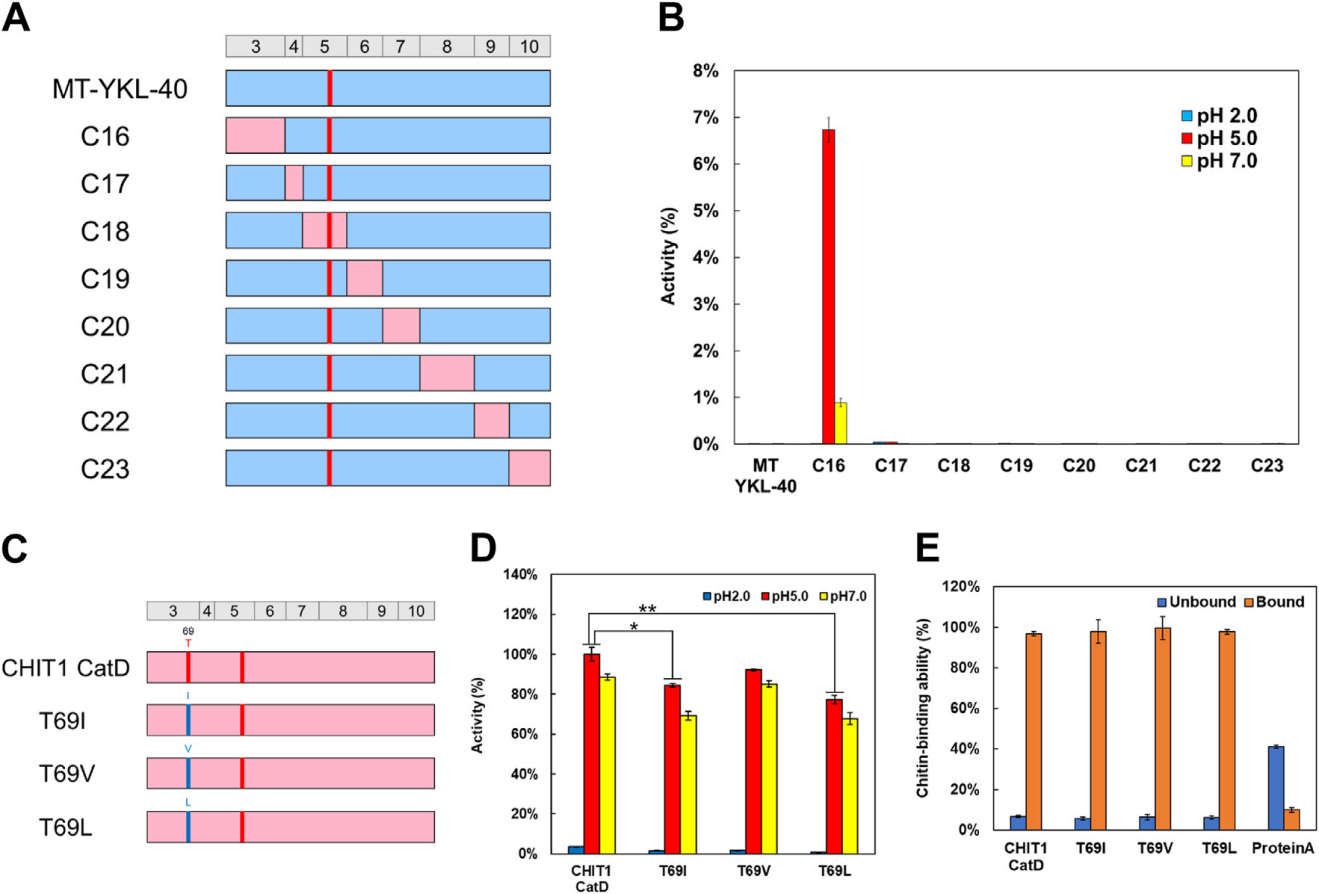
**Identification of key amino acids for chitinase inactivation.***A*, schematic representation of MT-YKL-40-CHIT1 CatD chimeras (C16-C23). *B*, comparison of chitinase activity of C16-C23. Error bars represent mean ± SD from a single experiment conducted in triplicate. *C*, schematic representation of WT-CHIT1 CatD and mutant proteins (T69I, T69V, or T69L). *D*, chitinase activity of CHIT1 CatD mutants. Error bars represent mean ± SD from a single experiment conducted in triplicate. The *p*-values were determined by Welch’s *t* test. ∗*p* < 0.05, ∗∗*p* < 0.01. *E*, investigation of chitin-binding activity of CHIT1CatD mutants. Error bars represent the mean ± SD from a single experiment conducted in triplicate. CatD, catalytic domain; CHIT1, chitotriosidase.

We have shown that MT-YKL-40 can be activated by introducing CHIT1 CatD exons 3, 6, and 8 (Fig. 3). Subsequently, we conducted a detailed examination of the primary structural modifications that impair chitinase activity in MT-YKL-40 while preserving the chitin-binding activity. To achieve this, we substituted MT-YKL-40 exons one by one with corresponding CHIT1 CatD exons (chimeras C16 to C23) (Figs. 5*A* and S3) and measured chitinase activity.

Most MT-YKL-40 and chimeric constructs exhibited minimal to no activity (Figs. 5*B*; S2 and S3 and Table S1). Notably, only chimera C16, with exon 3 replacement, displayed approximately 7% of the enzyme activity observed in Chit1 CatD, indicating that exon 3 is one of the major contributors to the inactivity of YKL-40.

To better understand the evolution of YKL-40 and CHIT1 CatD, we collected gene sequences from 28 primates using the National Center for Biotechnology Information (NCBI) database, adding mouse, rat, and pig Chit1 and YKL-40 (Chi3l1) as an outgroup. Using the CODEML program in the PAML package (58), we conducted evolutionary analysis with three pairs of site models to investigate potential positive selection in YKL-40 or CHIT1 CatD among primates. For YKL-40, Arg at position 304 (R304) (exon 9) was identified as being under positive selection (Table 1). Similarly, analyses of CHIT1 CatD revealed two sites, Thr at position 69 (T69) (exon 3) and Tyr at position 190 (Y190) (exon 6), inferred to be under positive selection (Table 1). Thus, evolutionary analysis also highlights T69 (exon 3) in CHIT1 CatD as a positively selected amino acid.

**Table 1:** Evolutionary analysis of YKL-40 and CHIT1 using PAML CODEML site model

Gene family	Model	lnL	np	TL	*κ*	Parameter estimates	Test	LR	*p*-Value	Positively selected sites
***YKL-40***	M0	−6971.3	62	15.1	3.59	*ω* = 0.216				
	M1a	−6897.9	63	35.1	3.78	*ω*_*0*_ = 0.0826 (79.8%)				
						*ω*_*1*_ = 1.000 (20.2%)				
	M2a	−6897.9	65	35.1	3.78	*ω*_*0*_ = 0.0826 (79.8%)	M1a *versus* M2a	0.000	1.000	Not allowed
						*ω*_*1*_ = 1.000 (0%)				
						*ω*_*2*_ = 1.000 (20.2%)				
	M7	−6882.4	63	52.6	3.71	*p* = 0.560				
						*q* = 1.88				
	M8	−6876.2	65	52.6	3.75	*p*_*0*_ = 0.891	M7 *versus* M8	12.5	*P* < 0.005	304R (0.955∗)
						*p* = 0.757				
						*q* = 4.54				
						*ω* = 1.000 (10.9%)				
***CHIT1***	M0	−6166.5	62	3.14	4.02	*ω* = 0.284				
	M1a	−6076.9	63	3.23	4.20	*ω*_*0*_ = 0.123 (77.8%)				
						*ω*_*1*_ = 1.000 (22.2%)				
	M2a	−6076.9	65	3.23	4.20	*ω*_*0*_ = 0.123 (77.8%)	M1a *versus* M2a	0.000	1.000	Not allowed
						*ω*_*1*_ =1.000 (19.1%)				
						*ω*_*2*_ =1.000 (3.10%)				
	M7	−6073.6	63	3.24	4.09	*p* = 0.352				
						*q* = 0.851				
	M8	−6065.8	65	3.26	4.18	*p*_*0*_ = 0.974	M7 *versus* M8	15.6	*P* < 0.001	69T (0.993∗∗), 190Y (0.984∗)
						*p* = 0.451				
						*q* = 1.26				
						*ω* = 2.31 (2.56%)				

Our exon scanning results further emphasize the role of CHIT1 CatD exon 3 in activating MT-YKL-40. Evolutionary analysis suggests that the amino acid at position 69 in exon 3 of CHIT1 CatD contributes to chitinase activity. In the human variant, this amino acid is Thr (T), but in other primates, it can be Ile (I), Val (V), or Leu (L) (Fig. S5). Therefore, we created mutants by replacing T at position 69 with these three amino acids (Figs. 5*C* and S3) and examined their chitinase and chitin-binding activity. Introducing T69I and T69L significantly reduced the chitinase activity, while T69V resulted in a less pronounced decrease (Figs. 5*D*; S2 and S3 and Table S1). As for chitin binding, there was no difference in activity among the mutants (Fig. 5*E*). These findings indicate that, although the amino acid 69 in CHIT1 was positively selected in humans to enhance chitinase activity while having minimal impact on chitin binding in CHIT1 CatD.

### Effect of amino acid substitutions on chitinase and chitin-binding activity in CHIT1 CatD and MT-YKL-40

**Figure 6:**
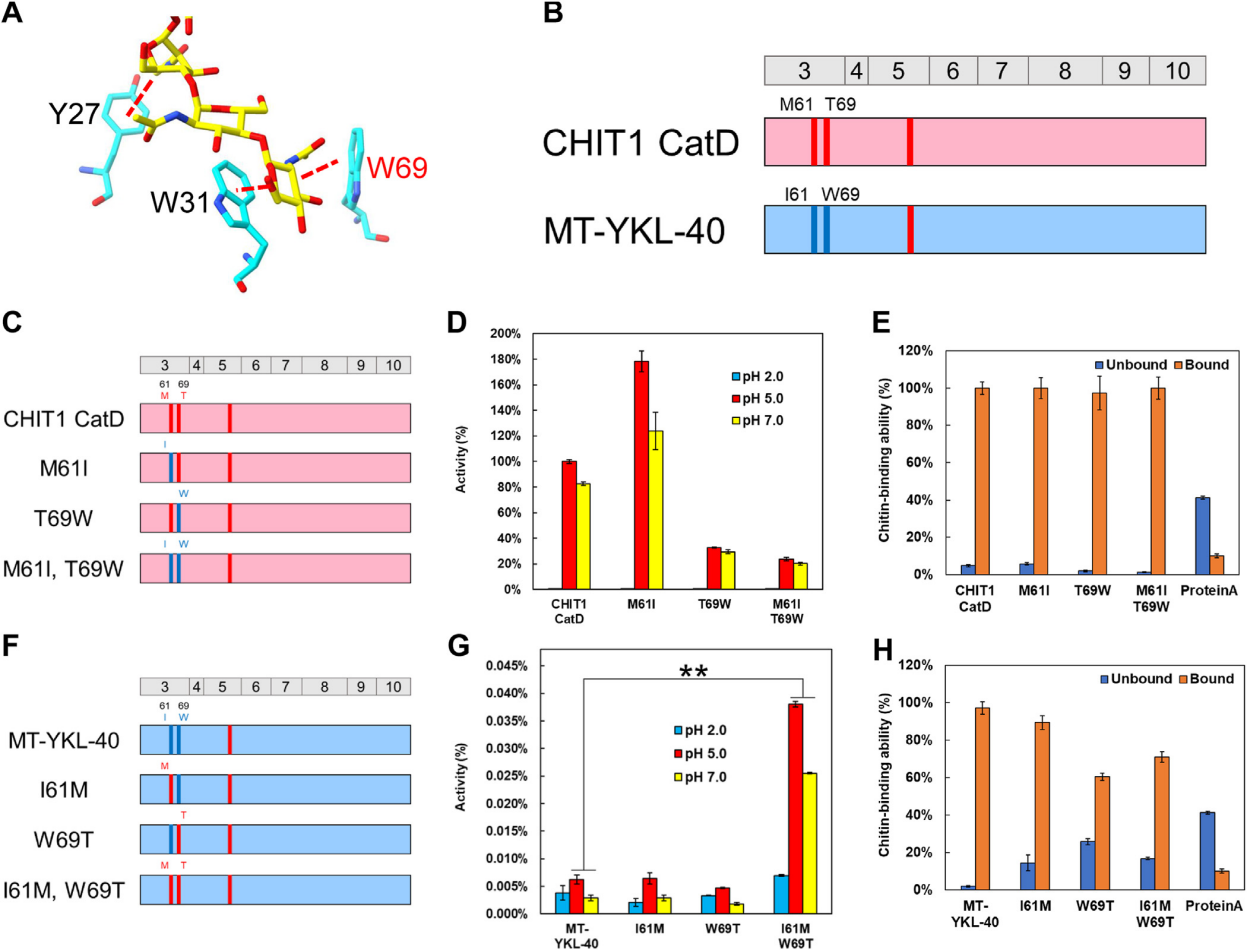
**Effect of amino acid substitutions on chitinase and chitin-binding activity in CHIT1 CatD and MT-YKL-40.***A*, W69 involvement in substrate binding. The three-dimensional structure of YKL-40 is based on the reported crystal structure (PDB accession code 1NWT). Amino acid residues of YKL-40 are shown in *cyan*, and GlcNAc oligomer in *yellow*. Known X-ray crystallographic analysis of catalytic motif, surrounding amino acids, and binding of substrate chitin oligomers. Amino acid residues with hydrophobic interactions are schematically drawn as dashed *red* lines. *B*, schematic representation of key amino acids in CHIT1 CatD and MT-YKL-40. The amino acid sequences are color coded: *pink*, CHIT1 CatD; *blue*, YKL-40 sequence. Human CHIT1 CatD has M61 and T69; YKL-40 has I61 and W69. *C*, schematic representation of WT-CHIT1 CatD and mutants M61I, T69W, and M61I/T69W. *D*, chitinase activity of CHIT1 mutants. Error bars represent mean ± SD from a single experiment conducted in triplicate. *E*, chitin-binding activity of CHIT1 mutants. Error bars represent mean ± SD from a single experiment conducted in triplicate. *F*, schematic representation of MT-YKL-40 and mutants I61M, W69T, and I61M/W69T. *G*, chitinase activity of YKL-40 mutants. Error bars represent mean ± SD from a single experiment conducted in triplicate. The *p*-values were determined by Welch’s *t* test. ∗∗*p* < 0.01. *H*, chitin-binding activity in MT-YKL-40 mutants. Error bars represent mean ± SD from a single experiment conducted in triplicate. CatD, catalytic domain; CHIT1, chitotriosidase; PDB, Protein Data Bank.

Building on our findings regarding the critical amino acids in exon 3, we explored their relationship with the catalytic motif and surrounding amino acids, as revealed by X-ray crystallographic analyses (54, 55, 56). A key discovery was that Trp (W) at position 69 (W69) in MT-YKL-40 significantly influences enzyme-substrate affinity in exon 3 (Fig. 6*A*) (55). In contrast, human CHIT1 CatD contains T69. Substituting T69 with other amino acids (I, V, or L) in CHIT1 CatD resulted in a substantial decrease in chitinase activity, as shown in Figure 5*D*. These data highlight the pivotal role of the amino acid at position 69 (W69 in MT-YKL-40 and T69 in CHIT1 CatD) in substrate binding and degradation (54, 55, 56).

Furthermore, our investigation extended to another chitinase, human CHIA, where Met (M) at position 61 in exon 3 is reported to be crucial for chitinase activity (52). In contrast, activity in CHIA with Ile (I) is low. Given that YKL-40 contains I at position 61 and CHIT1 contain M at position 61, resembling CHIA, we examined the impact of these amino acids at positions 61 and 69 on chitinase and chitin-binding activity by generating several amino acid mutants (Fig. 6*B*).

The structural setup for the tested proteins is shown in Figure 6*C* and Fig. S3. In human CHIT1, Met (M) at position 61 and/or Thr (T) at position 69 were substituted with Ile (I) and Trp (W), respectively.

The M61I mutation in CHIT1 CatD increased enzyme activity by almost 80%, while the T69W and compound mutations led to an approximately 70 and 80% decrease in activity, respectively (Figs. 6*D*; S2 and S3 and Table S1). As for the chitin-binding activity, M61I substitution had no significant effect, but substituting T69W and the compound mutation resulted in a slight increase (Fig. 6*E*).

Similar experiments were conducted with MT-YKL-40 with mutations I61M and/or W69T (Figs. 6*F* and S3). Evaluating chitinase activity, individual substitutions at positions 61 and 69 did not lead to activation (Figs. 6*G*; S2 and S3 and Table S1). However, the simultaneous introduction of the mutations resulted in still very low (less than 0.04% of the CHIT1 CatD) but severalfold increased chitinase activity (Fig. 6*G*). The mutation of I61M led to a slight decrease and W69T to an approximately 40% reduction in chitin-binding activity. Similarly, introducing both mutations led to a decrease by 30% (Fig. 6*H*).

These findings reveal the critical role of specific amino acids in regulating chitinase activity and substrate binding in CHIT1 CatD and MT-YKL-40, shedding light on their structural and functional characteristics.

### Structural comparison of YKL-40 with CHIT1 and YKL-39

**Figure 7:**
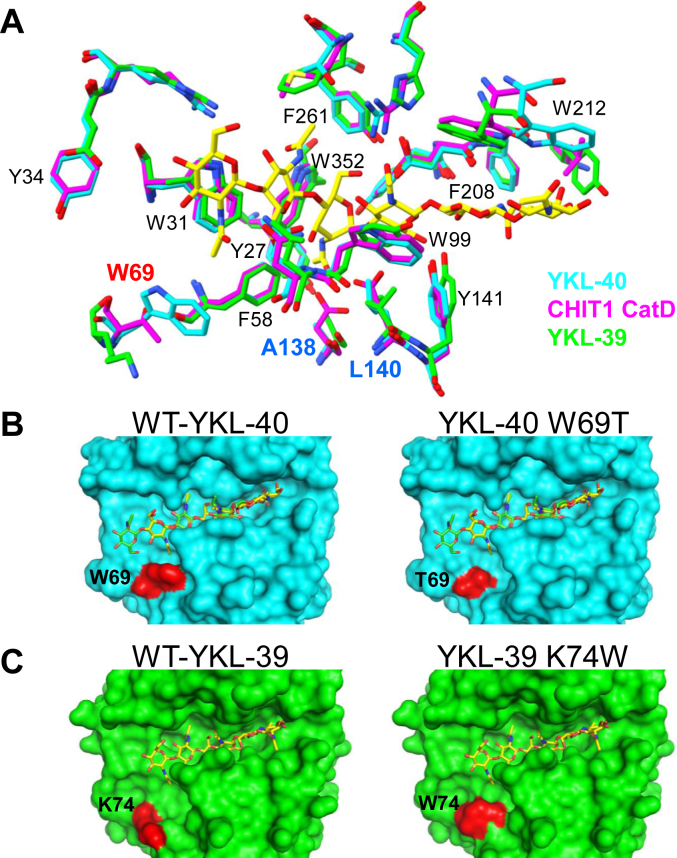
**Superimposition of YKL-40 and its human GH-18 homologs of CHIT1 CatD and YKL-39.***A*, structural overlay showing YKL-40 (*cyan*) with PDB accession code 1NWT, CHIT1 CatD (*magenta*) with PDB accession code 1LG1, and YKL-39 (*green*) with PDB accession code 4P8X. Amino acid residues involved in sugar binding are shown. For YKL-40, residues such as W69 that are crucial for substrate binding are specifically detailed with their position numbers and single-letter abbreviations to facilitate clear identification. Only the (GlcNAc)_6_ complex with YKL-40 is displayed (*yellow*) to highlight these interactions. *B*, surface representation of the sugar-binding cleft of YKL-40, showing chitohexaose occupying subsites −3 to +3 (PDB accession code 1NWT, *yellow*) or subsites −4 to +2 (PDB accession code 1HJW, *green*). W69T substitution causes notable differences in the sugar-binding cleft (*left*: W69 with the groove part for binding recognition is longer; *right*: T69 with a shorter groove). The amino acid at position 69 is highlighted in *red*. *C*, surface representation of the sugar-binding cleft of YKL-39, showing chitohexaose occupying subsites −3 to +3 (PDB accession code 4P8X, *yellow*). K74W substitution causes notable differences in the sugar-binding cleft (*left*: K74 with the groove part for binding recognition is shorter; *right*: W74 with a longer groove). The amino acid at position 74 is highlighted in *red*. Note: YKL-39 has a 5-amino-acid longer signal sequence than YKL-40, making position 74 in YKL-39 equivalent to position 69 in YKL-40. CatD, catalytic domain; CHIT1, chitotriosidase; PDB, Protein Data Bank.

We compared the subsites of CHIT1 and chitinase-like protein YKL-39 within the GH18 family (Fig. 7*A*). Notably, the position and shape of Trp at position 69 differ significantly between the YKL-39 type and the CHIT1 type. While other areas also show differences, the position 69 plays a significant role in substrate binding. Interestingly, W69 is unique to YKL-40 and is absent in CHIT1 or YKL-39, highlighting its crucial role in chitin binding for YKL-40 (Fig. 7*A*).

Additionally, the substrate-binding groove in YKL-40 (Fig. 7*B*) is longer than in YKL-39 (Fig. 7*C*), as previously reported (21). The region marked in red is occupied by W, and due to the size of this amino acid, it forms a larger and longer cleft (Fig. 7*B*). Conversely, in YKL-39, this region is occupied by Lys (K), widening the groove, with the binding site limited to subsites from −3 to +3 (Fig. 7*C*). In YKL-40, the presence of the large amino acid allows for accommodation of subsites from −4 to +2 and from −3 to +3. We speculate that replacing W69 in YKL-40 with T69 could shorten the groove and alter substrate recognition (Fig. 7*B*). On the other hand, substituting W for the corresponding area in YKL-39 could lengthen the groove and enhance substrate recognition, akin to YKL-40 (Fig. 7*C*).

These structural models comparisons highlight the unique adaptations of YKL-40 in chitin binding, facilitated by the presence of W69, which significantly alters the groove dimensions and substrate interactions. This insight contributes to our understanding of the functional diversification within chitinase and chitinase-like proteins.

### Functional insights into YKL-40 with retained chitin-binding ability and loss of chitin-degrading activity

**Figure 8:**
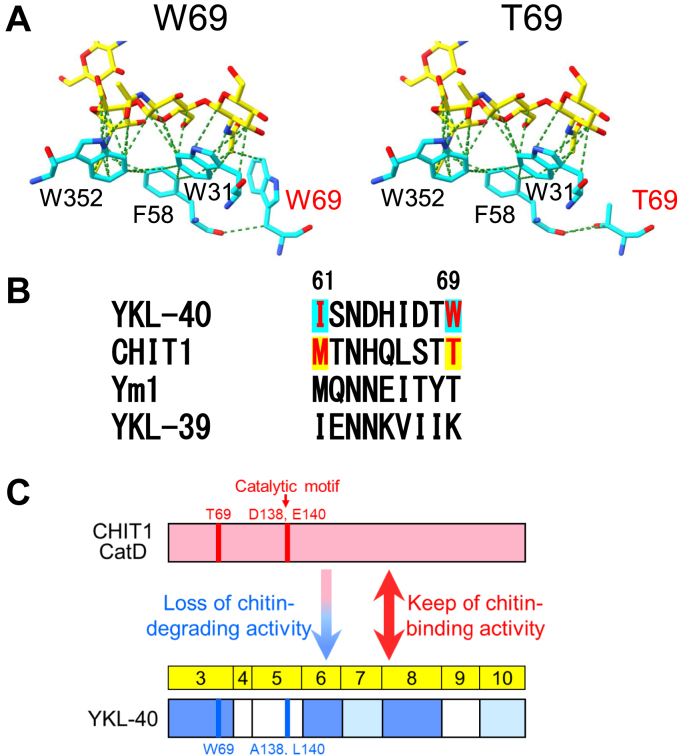
**Functional insights into YKL-40 with retained chitin-binding ability and loss of chitin-degrading activity.***A*, the three-dimensional structure of YKL-40 is based on the reported crystal structure (PDB accession code 1NWT). Amino acid residues of YKL-40 are shown in *cyan*, and GlcNAc oligomer in *yellow*. W69T substitution causes notable differences in the three-dimensional structure (*left*: W69 interacting with the substrate; *right*: T69 lacking this interaction). W69 also interacts with F58, which interacts with the substrate and forms strong interactions with W31 and W352. W31 and W352 play a crucial role in substrate interactions, with W352 forming hydrogen bonds with the substrate, contributing significantly to substrate recognition. Substituting W69 with T69 alters the interaction with F58, leading to cascading effects on W31 and W352, potentially influencing chitinase activity and chitin-binding properties. *B*, comparison of amino acids at positions 61 and 69 between YKL-40, CHIT1, Ym1, and YKL-39. The crucial role of amino acid W at position 69 in YKL-40's strong chitin binding, distinct from Ym1 and YKL-39, underscores its unique evolutionary path. *C*, major causes of YKL-40 inactivation: two amino acid substitutions in the catalytic motif (*blue line*) and amino acid substitutions at exons 3, 6, and 8 (*blue boxes*), especially W at position 69 (*blue line*). Additionally, amino acid substitutions at exons 7 and 10 are weakly involved in the reduction (*light blue boxes*). The *red arrow* indicates the position of the catalytic motif. Catalytic motif (D138 and E140) in CHIT1 and their amino acid substitutions (A138 and L140) in YKL-40 are shown in *red* and *blue*, respectively. *Yellow boxes* indicate exons with numbers conserved between CHIT1 CatD and YKL-40 cDNAs. CatD, catalytic domain; cDNA, complementary DNA; CHIT1, chitotriosidase; PDB, Protein Data Bank.

As revealed by this study, the diminished chitinase activity caused by the introduction of MT-YKL-40 exon 3 into CHIT1 CatD is intricately linked to specific amino acids—I61 and W69. The W69T substitution in YKL-40 causes differences in the three-dimensional structure (Fig. 8*A*). While W69 interacts with the substrate, T69 lacks this interaction. Furthermore, W69 interacts with F58, and this interaction becomes stronger upon the W69T substitution. F58 also interacts with the substrate and strongly interacts with W31 and W352. W31 and W352 form strong interactions with the substrate and are highly conserved among other mammalian CLPs. In particular, W352 forms hydrogen bonds with the substrate, playing a significant role in substrate recognition (55). Thus, introducing the W69T substitution can alter the interaction with F58, leading to cascading effects on W31 and W352, potentially influencing chitinase activity and chitin-binding properties.

Our investigation into the evolutionary trajectory of related CLPs revealed distinctive patterns. For instance, replacing two amino acids at the catalytic motif in Ym1 with a Chia-like sequence (N136D and Q140E) failed to activate Ym1, suggesting other significant amino acid substitutions over its evolutionary history. This led to the loss of its ability to recognize or bind chitin substrates and subsequent impairment of chitin-degrading activity (59). In contrast, YKL-39, while exhibiting chitooligosaccharide binding (21), demonstrated slight but evident activation upon introducing two amino acids within the catalytic motif (20). Similarly, YKL-40 displays robust affinity to chitin and it did not generally respond to the two amino acids mutations within the catalytic motif but could be activated by substituting specific amino acids. The crucial role of amino acid W at position 69 in YKL-40's strong affinity to chitin, distinct from Ym1 and YKL-39, highlight its unique evolutionary path (Fig. 8*B*).

In this study, we found that YKL-40 inactivation is primarily attributed to amino acid substitutions at catalytic motif and exons 3, 6, and 8, with additional contributions in exons 7 and 10 (Fig. 8*C*). The reduction in chitinase activity in exon 3 of MT-YKL-40 is linked to I61 and W69 (Fig. 7*C*). Our findings affirm that YKL-40 retains the structural framework of a mammalian chitinase, maintaining chitin recognition while losing chitin degradation, a characteristic acquired through evolution, marked by the introduction of W at position 69 (Fig. 8).

Structurally resembling CHIT1 CatD, YKL-40, with coding exons 3 to 10, has lost its enzymatic function due to multiple amino acid substitutions across the molecule. We propose an evolutionary connection between YKL-40 and CHIT1, supported by their proximity on chromosome 1 and higher sequence homology to CHIT1 than CHIA. The pH dependency analysis of chitinase activity for the chimeric protein revealed similar profiles to CHIT1 at various pH levels, reinforcing the hypothesis that YKL-40 likely evolved from CHIT1 (Figs. 2, 3, 5 and 6).

Our study identifies key regions within YKL-40 that, when altered, significantly affect its function. Targeting these specific regions could help control its activity in various disease states involving YKL-40. Our findings suggest the potential for developing therapeutic strategies designed to modify YKL-40's role in disease processes.

## Experimental procedures

### Construction of YKL-40 vector for *E. coli* expression

Mature YKL-40 was derived from human lung tissue complementary DNA (cDNA) by PCR using KOD-Plus-DNA polymerase (Toyobo) and oligonucleotide primers (Eurofins Genomics) anchored with the restriction sites for BamHI and XhoI as described previously (Table S2) (60). We used BamHI and Xhol to cut the amplified cDNA and cloned it into the same site of pET22b/pre-Protein A-monkey AMCase-V5-His (61, 62). The complete sequence of the obtained plasmid DNA (pET22b/YKL-40/V5-His) was identified by sequencing (Eurofins Genomics). We constructed MT-YKL-40 and MT-YKL-40-CHIT1 CatD chimeras by site-directed mutagenesis by primer extension (Table S2) (52). YKL-40 and CHIT1 CatD mutant proteins were prepared by PCR using a template and primers (Table S2) (51).

### Preparation of recombinant CHIT1 and YKL-40 proteins expressed in *E. coli*

Preparation of Protein A-CHIT1-V5-His and Protein A-YKL-40-V5-His from the *E. coli* was performed and purified by Ni Sepharose (GE HealthCare) as described (60).

### SDS-PAGE and Western blot

The protein fractions were analyzed using standard SDS-PAGE, followed by Western blot using an anti-V5-HRP monoclonal antibody (Thermo Fisher Scientific). Immunoblots were analyzed and quantified using the Luminescent Image Analyzer (ImageQuant LAS 4000, GE HealthCare) according to the manufacturer's instructions.

### Chitinase enzymatic assays

Chitinase enzyme activity was determined with the fluorogenic substrate 4-MU β-D- *N, N′*-diacetylchitobioside hydrate (Sigma-Aldrich) as a substrate in McIlvaine’s buffer (0.1 M citric acid and 0.2 M Na_2_HPO_4_; pH 2.0, 5.0, and 7.0) at 37 °C for 30 min as described previously (51). The fluorescence of released 4-MU was measured using GloMax Discover System GM3000 (Promega) with excitation at 365 nm and emission at 415 to 445 nm.

### Chitin binding assay

Chitin binding assays are performed as described previously (63, 64). Briefly, chitin beads column (New England Biolabs) column equilibration was performed using McIlvaine buffer (pH 7.0) and 0.5 M NaCl. Bound proteins were eluted with 8 M urea/2%-SDS/2.5% 2-mercaptoethanol solution.

### Evolutional analysis

Primate YKL-40 and CHIT1 CatD cDNA sequences were obtained from the National Center for Biotechnology Information GenBank (Table S3). Rate ratios of nonsynonymous-to-synonymous substitutions (dN/dS) were calculated using the CODEML program within PAML (58) to compare the primate YKL-40 and CHIT1 CatD after removing the nucleotide sequences encoding the signal peptides and stop codons (Supplementary data sets). Specifically, a site model was used, allowing the ω ratio to vary among sites to detect signatures of positive selection at each codon, as previously described (59).

### Analysis of structural changes in MT-YKL-40 with amino acid substitutions

Using AlphaFold2 (65, 66) and PyMOL (https://pymol.org/, Molecular Graphics System, Version 3.0.0, Schrödinger, LLC), we introduced amino acid substitutions into YKL-40 and YKL-39. We then applied the interaction analysis command (Contacts) in ChimeraX (67) to examine the structural changes in MT-YKL-40 and their mutant proteins, focusing on how the 69th amino acid interact with the substrate, GlcNAc oligomer. Additionally, we analyzed the changes in interactions between the W69T substitution and surrounding amino acids using the same command in ChimeraX.

### Statistical analysis

Our experiments were conducted in triplicate in statistical evaluation. Biochemical data were subjected to comparison using Welch’s *t* test.

## Data availability

Data supporting the reported results will be available from the corresponding author (Fumitaka Oyama).

## Supporting information

This article contains supporting information.

## Conflict of interest

The authors declare that they have no conflicts of interest with the contents of this article.
