# Supplementary material for: Evolutionary insights into sequence modifications governing chitin recognition and chitinase inactivity in YKL-40 (HC-gp39, CHI3L1)
Source: J Biol Chem. 2024 May 13;300(6):107365. doi: 10.1016/j.jbc.2024.107365 (PMC11190707; doi:10.1016/j.jbc.2024.107365)
Supplement: Supporting Information [file mmc1.pdf]

Evolutionary insights into sequence modifications governing chitin recognition and chitinase inactivity in YKL-40 (HC-gp39, CHI3L1)

Keita Suzuki[1], Kazuaki Okawa[1], Masashi Ohkura[1], Tomoki Kanaizumi[1], Takaki Kobayashi[1], Koro Takahashi[1], Hiromu Takei[1], Momo Otsuka[1], Eri Tabata[1,2], Peter O. Bauer[3] and Fumitaka Oyama[1,*]

Supplementary Figs. S1-S5, Supplementary Tables S1-S3, and Supplementary data set 1 and 2.

A. Pre Protein A-CHIT1 CatD-V5-His
569 amino acids 63,110 dalton

MKKKNIYSIRKLGVGIASVTLGTLLISGGVTPAANAAQHDEAVDNKFNKEQQNAFYEIL
HLPNLNEEQRNAFIQSLKDDPSQSANLLAEAKKLNDAQAPKVDNKFNKEQQNAFYEILH
LPNLNEEQRNAFIQSLKDDPSQSANLLAEAKKLNDAQAPKVDANSSSVPGDPAKLVCYF
TNWAQYRQGEARFLPKDLDPSLCTHLIYAFAGMTNHQLSTTEWNDETLYQEFNGLKKMN
PKLKTLLAIGGWNFSTQKFTDMVATANNRQTFVNSAIRFLRKYSFDGLDLDWEYPGSQG
SPAVDKERFTTLVQDLANAFQQEAQTSGKERLLLSAAVPAGQTYVDAGYEVDKIAQNLD
FVNLMAYDFHGSWEKVTGHNSPLYKRQEESGAAASLNVDAAVQQWLQKGTPASKLILGM
PTYGRSFTLASSSDTRVGAPATGSGTPGPFTKEGGMLAYYEVCSWKGATKQRIQDQKVP
YIFRDNQWVGFDDVESFKTKVSYLKQKGLGGAMVWALDLDDFAGFSCNQGRYPLIQTLR
QELSLPAAARGHPFEGKPIPNPLLGLDSTRTGHHHHHH

B. Mature Protein A-CHIT1 CatD-V5-His
533 amino acids 59,484 dalton

AQHDEAVDNKFNKEQQNAFYEILHLPNLNEEQRNAFIQSLKDDPSQSANLLAEAKKLND
AQAPKVDNKFNKEQQNAFYEILHLPNLNEEQRNAFIQSLKDDPSQSANLLAEAKKLNDA
QAPKVDANSSSVPGDPAKLVCYFTNWAQYRQGEARFLPKDLDPSLCTHLIYAFAGMTNH
QLSTTEWNDETLYQEFNGLKKMNPKLKTLLAIGGWNFSTQKFTDMVATANNRQTFVNSA
IRFLRKYSFDGLDLDWEYPGSQGSPAVDKERFTTLVQDLANAFQQEAQTSGKERLLLSA
AVPAGQTYVDAGYEVDKIAQNLDFVNLMAYDFHGSWEKVTGHNSPLYKRQEESGAAASL
NVDAAVQQWLQKGTPASKLILGMPTYGRSFTLASSSDTRVGAPATGSGTPGPFTKEGGM
LAYYEVCSWKGATKQRIQDQKVPYIFRDNQWVGFDDVESFKTKVSYLKQKGLGGAMVWA
LDLDDFAGFSCNQGRYPLIQTLRQELSLPAAARGHPFEGKPIPNPLLGLDSTRTGHHHH
HH

C. Pre Protein A-WT-YKL-40-V5-His
564 amino acids 62,615 dalton

MKKKNIYSIRKLGVGIASVTLGTLLISGGVTPAANAAQHDEAVDNKFNKEQQNAFYEIL
HLPNLNEEQRNAFIQSLKDDPSQSANLLAEAKKLNDAQAPKVDNKFNKEQQNAFYEILH
LPNLNEEQRNAFIQSLKDDPSQSANLLAEAKKLNDAQAPKVDANSSSVPGDPYKLVCYY
TSWSQYREGDGSCFPDALDRFLCTHIIYSFANISNDHIDTWEWNDVTLYGMLNTLKNRN
PNLKTLLSVGGWNFGSQRFSKIASNTQSRRTFIKSVPPFLRTHGFDGLDLAWLYPGRRD
KQHFTTLIKEMKAEFIKEAQPGKKQLLLSAALSAGKVTIDSSYDIAKISQHLDFISIMT
YDFHGAWRGTTGHHSPLFRGQEDASPDRFSNTDYAVGYMLRLGAPASKLVMGIPTFGRS
FTLASSETGVGAPISGPGIPGRFTKEAGTLAYYEICDFLRGATVHRILGQQVPYATKGN
QWVGYDDQESVKSKVQYLKDRQLAGAMVWALDLDDFQGSFCGQDLRFPLTNAIKDALAA
TAAARGHPFEGKPIPNPLLGLDSTRTGHHHHHH

D. Mature Protein A-WT-YKL-40-V5-His
528 amino acids 58,989 dalton

AQHDEAVDNKFNKEQQNAFYEILHLPNLNEEQRNAFIQSLKDDPSQSANLLAEAKKLND
AQAPKVDNKFNKEQQNAFYEILHLPNLNEEQRNAFIQSLKDDPSQSANLLAEAKKLNDA
QAPKVDANSSSVPGDPYKLVCYYTSWSQYREGDGSCFPDALDRFLCTHIIYSFANISND
HIDTWEWNDVTLYGMLNTLKNRNPNLKTLLSVGGWNFGSQRFSKIASNTQSRRTFIKSV
PPFLRTHGFDGLDLAWLYPGRRDKQHFTTLIKEMKAEFIKEAQPGKKQLLLSAALSAGK
VTIDSSYDIAKISQHLDFISIMTYDFHGAWRGTTGHHSPLFRGQEDASPDRFSNTDYAV
GYMLRLGAPASKLVMGIPTFGRSFTLASSETGVGAPISGPGIPGRFTKEAGTLAYYEIC
DFLRGATVHRILGQQVPYATKGNQWVGYDDQESVKSKVQYLKDRQLAGAMVWALDLDDF
QGSFCGQDLRFPLTNAIKDALAATAAARGHPFEGKPIPNPLLGLDSTRTGHHHHHH

E. Pre Protein A-MT-YKL-40-V5-His
564 amino acids 62,674 dalton

MKKKNIYSIRKLGVGIASVTLGTLLISGGVTPAANAAQHDEAVDNKFNKEQQNAFYEIL
HLPNLNEEQRNAFIQSLKDDPSQSANLLAEAKKLNDAQAPKVDNKFNKEQQNAFYEILH
LPNLNEEQRNAFIQSLKDDPSQSANLLAEAKKLNDAQAPKVDANSSSVPGDPYKLVCYY
TSWSQYREGDGSCFPDALDRFLCTHIIYSFANISNDHIDTWEWNDVTLYGMLNTLKNRN
PNLKTLLSVGGWNFGSQRFSKIASNTQSRRTFIKSVPPFLRTHGFDGLDLDWEYPGRRD
KQHFTTLIKEMKAEFIKEAQPGKKQLLLSAALSAGKVTIDSSYDIAKISQHLDFISIMT

YDFHGAWRGTTGHHSPLFRGQEDASPDRFSNTDYAVGYMLRLGAPASKLVMGIPTFGRS
FTLASSETGVGAPISGPGIPGRFTKEAGTLAYYEICDFLRGATVHRILGQQVPYATKGN
QWVGYDDQESVKSKVQYLKDRQLAGAMVWALDLDDFQGSFCGQDLRFPLTNAIKDALAA
TAAARGHPFEGKPIPNPLLGLDSTRTGHHHHHH

F. Mature Protein A-MT-YKL-40-V5-His
528 amino acids 59,049 dalton

AQHDEAVDNKFNKEQQNAFYEILHLPNLNEEQRNAFIQSLKDDPSQSANLLAEAKKLND
AQAPKVDNKFNKEQQNAFYEILHLPNLNEEQRNAFIQSLKDDPSQSANLLAEAKKLNDA
QAPKVDANSSSVPGDPYKLVCYYTSWSQYREGDGSCFPDALDRFLCTHIIYSFANISND
HIDTWEWNDVTLYGMLNTLKNRNPNLKTLLSVGGWNFGSQRFSKIASNTQSRRTFIKSV
PPFLRTHGFDGLDLDWEYPGRRDKQHFTTLIKEMKAEFIKEAQPGKKQLLLSAALSAGK
VTIDSSYDIAKISQHLDFISIMTYDFHGAWRGTTGHHSPLFRGQEDASPDRFSNTDYAV
GYMLRLGAPASKLVMGIPTFGRSFTLASSETGVGAPISGPGIPGRFTKEAGTLAYYEIC
DFLRGATVHRILGQQVPYATKGNQWVGYDDQESVKSKVQYLKDRQLAGAMVWALDLDDF
QGSFCGQDLRFPLTNAIKDALAATAAARGHPFEGKPIPNPLLGLDSTRTGHHHHHH

Supplementary Fig. S1. Deduced amino acid sequences and molecular masses of the recombinant CHIT1 CatD, WT-YKL-40 and MT-YKL-40 expressed in *E. coli.* The amino acid sequences are color coded, consistent with Fig. 1**A**. Blue, signal sequence of Protein A; Yellow, truncated form of Protein A; Pink, CHIT1 catalytic domain (CatD); Light blue, YKL-40; Green, V5-His sequence.

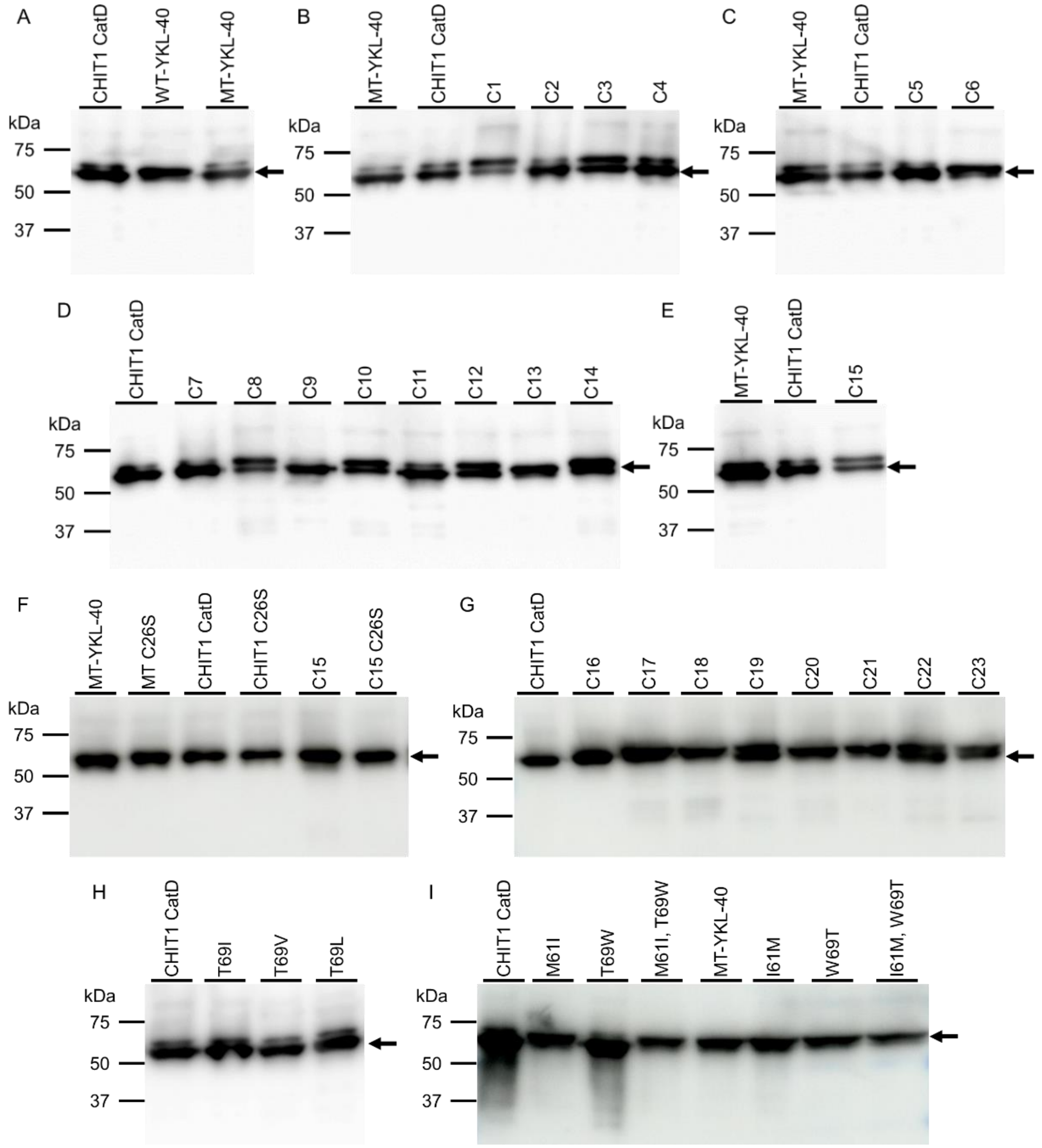

Supplementary Fig. S2. Western blot analysis of the recombinant proteins using anti-V5 antibody. Arrow highlights the positions of the fusion proteins. (A) Expressed CHIT1 CatD, WT-YKL-40, and MT-YKL-40. (B) Expressed CHIT1 CatD, MT-YKL-40, CHIT1 CatD, and MT-YKL-40-CHIT1 CatD chimeras (C1-C4). (C) Expressed MT-YKL-40, CHIT1 CatD, and MT-YKL-40-CHIT1 CatD chimeras (C5 and C6). (D) Expressed CHIT1 CatD, and MT-YKL-40-CHIT1 CatD chimeras (C7-C14). (E) Expressed MT-YKL-40, CHIT1 CatD, and MT-YKL-40-CHIT1 CatD chimera (C15). (F) Expressed MT-YKL-40, CHIT1 CatD, C15 and Cys26 mutations. (G) Expressed CHIT1 CatD, and MT-YKL-40-CHIT1 CatD chimeras (C16-C23). (H) Expressed CHIT1 CatD, and 69th Thr mutations. (I) Expressed CHIT1 CatD, MT-YKL-40 and each of mutations.

Chimera 1
YKLVCYYTSWSQYREGDGSCFPDALDRFLCTHIIYSFANISNDHIDTWEWNDVTLYGMLNTLKN
RNPNLKTLLSVGGWNFGSQRFSKIASNTQSRRTFIKSVPPFLRTHGFDGLDLDWEYPGRRDKQH
FTTLIKEMKAEFIKEAQPGKKQLLLSAALSAGKVTIDSSYDIAKISQHLDFISIMTYDFHGAWR
GTTGHHSPLFRGQEDASPDRFSNTDAAVQQWLQKGTPASKLILGMPTYGRSFTLASSSDTRVGA
PATGSGTPGPFTKEGGMLAYYEVCSWKGATKQRIQDQKVPYIFRDNQWVGFDDVESFKTKVSYL
KQKGLGGAMVWALDLDDFAGFSCNQGRYPLIQTLRQELSLP

Chimera 2
YKLVCYYTSWSQYREGDGSCFPDALDRFLCTHIIYSFANISNDHIDTWEWNDVTLYGMLNTLKN
RNPNLKTLLSVGGWNFGSQRFTDMVATANNRQTFVNSAIRFLRKYSFDGLDLDWEYPGSQGSPA
VDKERFTTLVQDLANAFQQEAQTSGKERLLLSAAVPAGQTYVDAGYEVDKIAQNLDFVNLMAYD
FHGSWEKVTGHNSPLYKRQEESGAAASLNVDAAVQQWLQKGTPASKLILGMPTYGRSFTLASSS
DTRVGAPATGSGTPGPFTKEGGMLAYYEVCSWKGATKQRIQDQKVPYIFRDNQWVGFDDVESFK
TKVSYLKQKGLGGAMVWALDLDDFAGFSCNQGRYPLIQTLRQELSLP

Chimera 3
AKLVCYFTNWAQYRQGEARFLPKDLDPSLCTHLIYAFAGMTNHQLSTTEWNDETLYQEFNGLKK
MNPKLKTLLAIGGWNFSTQKFTDMVATANNRQTFVNSAIRFLRKYSFDGLDLDWEYPGSQGSPA
VDKERFTTLVQDLANAFQQEAQTSGKERLLLSAAVPAGQTYVDAGYEVDKIAQNLDFVNLMAYD
FHGSWEKVTGHNSPLYKRQEESGAAASLNVDYAVGYMLRLGAPASKLVMGIPTFGRSFTLASSE
TGVGAPISGPGIPGRFTKEAGTLAYYEICDFLRGATVHRILGQQVPYATKGNQWVGYDDQESVK
SKVQYLKDRQLAGAMVWALDLDDFQGSFCGQDLRFPLTNAIKDALAAT

Chimera 4
AKLVCYFTNWAQYRQGEARFLPKDLDPSLCTHLIYAFAGMTNHQLSTTEWNDETLYQEFNGLKK
MNPKLKTLLAIGGWNFSTQKFSKIASNTQSRRTFIKSVPPFLRTHGFDGLDLDWEYPGRRDKQH
FTTLIKEMKAEFIKEAQPGKKQLLLSAALSAGKVTIDSSYDIAKISQHLDFISIMTYDFHGAWR
GTTGHHSPLFRGQEDASPDRFSNTDYAVGYMLRLGAPASKLVMGIPTFGRSFTLASSETGVGAP
ISGPGIPGRFTKEAGTLAYYEICDFLRGATVHRILGQQVPYATKGNQWVGYDDQESVKSKVQYL
KDRQLAGAMVWALDLDDFQGSFCGQDLRFPLTNAIKDALAAT

Chimera 5
YKLVCYYTSWSQYREGDGSCFPDALDRFLCTHIIYSFANISNDHIDTWEWNDVTLYGMLNTLKN
RNPNLKTLLSVGGWNFGSQRFTDMVATANNRQTFVNSAIRFLRKYSFDGLDLDWEYPGSQGSPA
VDKERFTTLVQDLANAFQQEAQTSGKERLLLSAAVPAGQTYVDAGYEVDKIAQNLDFVNLMAYD
FHGSWEKVTGHNSPLYKRQEESGAAASLNVDYAVGYMLRLGAPASKLVMGIPTFGRSFTLASSE
TGVGAPISGPGIPGRFTKEAGTLAYYEICDFLRGATVHRILGQQVPYATKGNQWVGYDDQESVK
SKVQYLKDRQLAGAMVWALDLDDFQGSFCGQDLRFPLTNAIKDALAAT

Chimera 6
AKLVCYFTNWAQYRQGEARFLPKDLDPSLCTHLIYAFAGMTNHQLSTTEWNDETLYQEFNGLKK
MNPKLKTLLAIGGWNFSTQKFSKIASNTQSRRTFIKSVPPFLRTHGFDGLDLDWEYPGRRDKQH
FTTLIKEMKAEFIKEAQPGKKQLLLSAALSAGKVTIDSSYDIAKISQHLDFISIMTYDFHGAWR
GTTGHHSPLFRGQEDASPDRFSNTDAAVQQWLQKGTPASKLILGMPTYGRSFTLASSSDTRVGA
PATGSGTPGPFTKEGGMLAYYEVCSWKGATKQRIQDQKVPYIFRDNQWVGFDDVESFKTKVSYL
KQKGLGGAMVWALDLDDFAGFSCNQGRYPLIQTLRQELSLP

Chimera 7
YKLVCYYTSWSQYREGDGSCFPDALDRFLCTHIIYSFANISNDHIDTWEWNDVTLYGMLNTLKN
RNPKLKTLLAIGGWNFSTQKFTDMVATANNRQTFVNSAIRFLRKYSFDGLDLDWEYPGSQGSPA
VDKERFTTLVQDLANAFQQEAQTSGKERLLLSAAVPAGQTYVDAGYEVDKIAQNLDFVNLMAYD
FHGSWEKVTGHNSPLYKRQEESGAAASLNVDAAVQQWLQKGTPASKLILGMPTYGRSFTLASSS
DTRVGAPATGSGTPGPFTKEGGMLAYYEVCSWKGATKQRIQDQKVPYIFRDNQWVGFDDVESFK
TKVSYLKQKGLGGAMVWALDLDDFAGFSCNQGRYPLIQTLRQELSLP

Chimera 8
AKLVCYFTNWAQYRQGEARFLPKDLDPSLCTHLIYAFAGMTNHQLSTTEWNDETLYQEFNGLKK
MNPNLKTLLSVGGWNFGSQRFTDMVATANNRQTFVNSAIRFLRKYSFDGLDLDWEYPGSQGSPA

VDKERFTTLVQDLANAFQQEAQTSGKERLLLSAAVPAGQTYVDAGYEVDKIAQNLDFVNLMAYD
FHGSWEKVTGHNSPLYKRQEESGAAASLNVDAAVQQWLQKGTPASKLILGMPTYGRSFTLASSS
DTRVGAPATGSGTPGPFTKEGGMLAYYEVCSWKGATKQRIQDQKVPYIFRDNQWVGFDDVESFK
TKVSYLKQKGLGGAMVWALDLDDFAGFSCNQGRYPLIQTLRQELSLP

Chimera 9

AKLVCYFTNWAQYRQGEARFLPKDLDPSLCTHLIYAFAGMTNHQLSTTEWNDETLYQEFNGLKK
MNPKLKTLLAIGGWNFSTQKFSKIASNTQSRRTFIKSVPPFLRTHGFDGLDLDWEYPGRRDKQH
FTTLIKDLANAFQQEAQTSGKERLLLSAAVPAGQTYVDAGYEVDKIAQNLDFVNLMAYDFHGSW
EKVTGHNSPLYKRQEESGAAASLNVDAAVQQWLQKGTPASKLILGMPTYGRSFTLASSSDTRVG
APATGSGTPGPFTKEGGMLAYYEVCSWKGATKQRIQDQKVPYIFRDNQWVGFDDVESFKTKVSY
LKQKGLGGAMVWALDLDDFAGFSCNQGRYPLIQTLRQELSLP

Chimera 10

AKLVCYFTNWAQYRQGEARFLPKDLDPSLCTHLIYAFAGMTNHQLSTTEWNDETLYQEFNGLKK
MNPKLKTLLAIGGWNFSTQKFTDMVATANNRQTFVNSAIRFLRKYSFDGLDLDWEYPGSQGSPA
VDKERFTTLVQEMKAEFIKEAQPGKKQLLLSAALSAGKVTIDSSYDIAKISQNLDFVNLMAYDF
HGSWEKVTGHNSPLYKRQEESGAAASLNVDAAVQQWLQKGTPASKLILGMPTYGRSFTLASSSD
TRVGAPATGSGTPGPFTKEGGMLAYYEVCSWKGATKQRIQDQKVPYIFRDNQWVGFDDVESFKT
KVSYLKQKGLGGAMVWALDLDDFAGFSCNQGRYPLIQTLRQELSLP

Chimera 11

AKLVCYFTNWAQYRQGEARFLPKDLDPSLCTHLIYAFAGMTNHQLSTTEWNDETLYQEFNGLKK
MNPKLKTLLAIGGWNFSTQKFTDMVATANNRQTFVNSAIRFLRKYSFDGLDLDWEYPGSQGSPA
VDKERFTTLVQDLANAFQQEAQTSGKERLLLSAAVPAGQTYVDAGYEVDKIAQHLDFISIMTYD
FHGAWRGTTGHHSPLFRGQEDASPDRFSNTDAAVQQWLQKGTPASKLILGMPTYGRSFTLASSS
DTRVGAPATGSGTPGPFTKEGGMLAYYEVCSWKGATKQRIQDQKVPYIFRDNQWVGFDDVESFK
TKVSYLKQKGLGGAMVWALDLDDFAGFSCNQGRYPLIQTLRQELSLP

Chimera 12

AKLVCYFTNWAQYRQGEARFLPKDLDPSLCTHLIYAFAGMTNHQLSTTEWNDETLYQEFNGLKK
MNPKLKTLLAIGGWNFSTQKFTDMVATANNRQTFVNSAIRFLRKYSFDGLDLDWEYPGSQGSPA
VDKERFTTLVQDLANAFQQEAQTSGKERLLLSAAVPAGQTYVDAGYEVDKIAQNLDFVNLMAYD
FHGSWEKVTGHNSPLYKRQEESGAAASLNVDYAVGYMLRLGAPASKLVMGIPTFGRSFTLASSE
TGVGAPISGPGIPGRFTKEAGTLAYYEVCSWKGATKQRIQDQKVPYIFRDNQWVGFDDVESFKT
KVSYLKQKGLGGAMVWALDLDDFAGFSCNQGRYPLIQTLRQELSLP

Chimera 13

AKLVCYFTNWAQYRQGEARFLPKDLDPSLCTHLIYAFAGMTNHQLSTTEWNDETLYQEFNGLKK
MNPKLKTLLAIGGWNFSTQKFTDMVATANNRQTFVNSAIRFLRKYSFDGLDLDWEYPGSQGSPA
VDKERFTTLVQDLANAFQQEAQTSGKERLLLSAAVPAGQTYVDAGYEVDKIAQNLDFVNLMAYD
FHGSWEKVTGHNSPLYKRQEESGAAASLNVDAAVQQWLQKGTPASKLILGMPTYGRSFTLASSS
DTRVGAPATGSGTPGPFTKEGGMLAYYEICDFLRGATVHRILGQQVPYATKGNQWVGYDDQESV
KSKVSYLKQKGLGGAMVWALDLDDFAGFSCNQGRYPLIQTLRQELSLP

Chimera 14

AKLVCYFTNWAQYRQGEARFLPKDLDPSLCTHLIYAFAGMTNHQLSTTEWNDETLYQEFNGLKK
MNPKLKTLLAIGGWNFSTQKFTDMVATANNRQTFVNSAIRFLRKYSFDGLDLDWEYPGSQGSPA
VDKERFTTLVQDLANAFQQEAQTSGKERLLLSAAVPAGQTYVDAGYEVDKIAQNLDFVNLMAYD
FHGSWEKVTGHNSPLYKRQEESGAAASLNVDAAVQQWLQKGTPASKLILGMPTYGRSFTLASSS
DTRVGAPATGSGTPGPFTKEGGMLAYYEVCSWKGATKQRIQDQKVPYIFRDNQWVGFDDVESFK
TKVQYLKDRQLAGAMVWALDLDDFQGSFCGQDLRFPLTNAIKDALAAT

Chimera 15

AKLVCYFTNWAQYRQGEARFLPKDLDPSLCTHLIYAFAGMTNHQLSTTEWNDETLYQEFNGLKK
MNPNLKTLLSVGGWNFGSQRFSKIASNTQSRRTFIKSVPPFLRTHGFDGLDLDWEYPGRRDKQH
FTTLIKDLANAFQQEAQTSGKERLLLSAAVPAGQTYVDAGYEVDKIAQHLDFISIMTYDFHGAW
RGTTGHHSPLFRGQEDASPDRFSNTDAAVQQWLQKGTPASKLILGMPTYGRSFTLASSSDTRVG

APATGSGTPGPFTKEGGMLAYYEICDFLRGATVHRILGQQVPYATKGNQWVGYDDQESVKSKVQ
YLKDRQLAGAMVWALDLDDFQGSFCGQDLRFPLTNAIKDALAAT

MT-YKL-40 C26S

YKLVSYYTSWSQYREGDGSCFPDALDRFLCTHIIYSFANISNDHIDTWEWNDVTLYGMLNTLKN
RNPNLKTLLSVGGWNFGSQRFSKIASNTQSRRTFIKSVPPFLRTHGFDGLDLDWEYPGRRDKQH
FTTLIKEMKAEFIKEAQPGKKQLLLSAALSAGKVTIDSSYDIAKISQHLDFISIMTYDFHGAWR
GTTGHHSPLFRGQEDASPDRFSNTDYAVGYMLRLGAPASKLVMGIPTFGRSFTLASSETGVGAP
ISGPGIPGRFTKEAGTLAYYEICDFLRGATVHRILGQQVPYATKGNQWVGYDDQESVKSKVQYL
KDRQLAGAMVWALDLDDFQGSFCGQDLRFPLTNAIKDALAAT

CHIT1 CatD C26S

AKLVSYFTNWAQYRQGEARFLPKDLDPSLCTHLIYAFAGMTNHQLSTTEWNDETLYQEFNGLKK
MNPKLKTLLAIGGWNFSTQKFTDMVATANNRQTFVNSAIRFLRKYSFDGLDLDWEYPGSQGSPA
VDKERFTTLVQDLANAFQQEAQTSGKERLLLSAAVPAGQTYVDAGYEVDKIAQNLDFVNLMAYD
FHGSWEKVTGHNSPLYKRQEESGAAASLNVDAAVQQWLQKGTPASKLILGMPTYGRSFTLASSS
DTRVGAPATGSGTPGPFTKEGGMLAYYEVCSWKGATKQRIQDQKVPYIFRDNQWVGFDDVESFK
TKVSYLKQKGLGGAMVWALDLDDFAGFSCNQGRYPLIQTLRQELSLP

C15 C26S

AKLVSYFTNWAQYRQGEARFLPKDLDPSLCTHLIYAFAGMTNHQLSTTEWNDETLYQEFNGLKK
MNPNLKTLLSVGGWNFGSQRFSKIASNTQSRRTFIKSVPPFLRTHGFDGLDLDWEYPGRRDKQH
FTTLIKDLANAFQQEAQTSGKERLLLSAAVPAGQTYVDAGYEVDKIAQHLDFISIMTYDFHGAW
RGTTGHHSPLFRGQEDASPDRFSNTDAAVQQWLQKGTPASKLILGMPTYGRSFTLASSSDTRVG
APATGSGTPGPFTKEGGMLAYYEICDFLRGATVHRILGQQVPYATKGNQWVGYDDQESVKSKVQ
YLKDRQLAGAMVWALDLDDFQGSFCGQDLRFPLTNAIKDALAAT

Chimera 16

AKLVCYFTNWAQYRQGEARFLPKDLDPSLCTHLIYAFAGMTNHQLSTTEWNDETLYQEFNGLKK
MNPNLKTLLSVGGWNFGSQRFSKIASNTQSRRTFIKSVPPFLRTHGFDGLDLDWEYPGRRDKQH
FTTLIKEMKAEFIKEAQPGKKQLLLSAALSAGKVTIDSSYDIAKISQHLDFISIMTYDFHGAWR
GTTGHHSPLFRGQEDASPDRFSNTDYAVGYMLRLGAPASKLVMGIPTFGRSFTLASSETGVGAP
ISGPGIPGRFTKEAGTLAYYEICDFLRGATVHRILGQQVPYATKGNQWVGYDDQESVKSKVQYL
KDRQLAGAMVWALDLDDFQGSFCGQDLRFPLTNAIKDALAAT

Chimera 17

YKLVCYYTSWSQYREGDGSCFPDALDRFLCTHIIYSFANISNDHIDTWEWNDVTLYGMLNTLKN
RNPKLKTLLAIGGWNFSTQKFSKIASNTQSRRTFIKSVPPFLRTHGFDGLDLDWEYPGRRDKQH
FTTLIKEMKAEFIKEAQPGKKQLLLSAALSAGKVTIDSSYDIAKISQHLDFISIMTYDFHGAWR
GTTGHHSPLFRGQEDASPDRFSNTDYAVGYMLRLGAPASKLVMGIPTFGRSFTLASSETGVGAP
ISGPGIPGRFTKEAGTLAYYEICDFLRGATVHRILGQQVPYATKGNQWVGYDDQESVKSKVQYL
KDRQLAGAMVWALDLDDFQGSFCGQDLRFPLTNAIKDALAAT

Chimera 18

YKLVCYYTSWSQYREGDGSCFPDALDRFLCTHIIYSFANISNDHIDTWEWNDVTLYGMLNTLKN
RNPNLKTLLSVGGWNFGSQRFTDMVATANNRQTFVNSAIRFLRKYSFDGLDLDWEYPGSQGSPA
VDKERFTTLVQEMKAEFIKEAQPGKKQLLLSAALSAGKVTIDSSYDIAKISQHLDFISIMTYDF
HGAWRGTTGHHSPLFRGQEDASPDRFSNTDYAVGYMLRLGAPASKLVMGIPTFGRSFTLASSET
GVGAPISGPGIPGRFTKEAGTLAYYEICDFLRGATVHRILGQQVPYATKGNQWVGYDDQESVKS
KVQYLKDRQLAGAMVWALDLDDFQGSFCGQDLRFPLTNAIKDALAAT

Chimera 19

YKLVCYYTSWSQYREGDGSCFPDALDRFLCTHIIYSFANISNDHIDTWEWNDVTLYGMLNTLKN
RNPNLKTLLSVGGWNFGSQRFSKIASNTQSRRTFIKSVPPFLRTHGFDGLDLDWEYPGRRDKQH
FTTLIKDLANAFQQEAQTSGKERLLLSAAVPAGQTYVDAGYEVDKIAQHLDFISIMTYDFHGAW
RGTTGHHSPLFRGQEDASPDRFSNTDYAVGYMLRLGAPASKLVMGIPTFGRSFTLASSETGVGA
PISGPGIPGRFTKEAGTLAYYEICDFLRGATVHRILGQQVPYATKGNQWVGYDDQESVKSKVQY
LKDRQLAGAMVWALDLDDFQGSFCGQDLRFPLTNAIKDALAAT

Chimera 20

YKLVCYYTSWSQYREGDGSCFPDALDRFLCTHIIYSFANISNDHIDTWEWNDVTLYGMLNTLKN
RNPNLKTLLSVGGWNFGSQRFSKIASNTQSRRTFIKSVPPFLRTHGFDGLDLDWEYPGRRDKQH
FTTLIKEMKAEFIKEAQPGKKQLLLSAALSAGKVTIDSSYDIAKISQNLDFVNLMAYDFHGSWE
KVTGHNSPLYKRQEESGAAASLNVDYAVGYMLRLGAPASKLVMGIPTFGRSFTLASSETGVGAP
ISGPGIPGRFTKEAGTLAYYEICDFLRGATVHRILGQQVPYATKGNQWVGYDDQESVKSKVQYL
KDRQLAGAMVWALDLDDFQGSFCGQDLRFPLTNAIKDALAAT

Chimera 21

YKLVCYYTSWSQYREGDGSCFPDALDRFLCTHIIYSFANISNDHIDTWEWNDVTLYGMLNTLKN
RNPNLKTLLSVGGWNFGSQRFSKIASNTQSRRTFIKSVPPFLRTHGFDGLDLDWEYPGRRDKQH
FTTLIKEMKAEFIKEAQPGKKQLLLSAALSAGKVTIDSSYDIAKISQHLDFISIMTYDFHGAWR
GTTGHHSPLFRGQEDASPDRFSNTDAAVQQWLQKGTPASKLILGMPTYGRSFTLASSSDTRVGA
PATGSGTPGPFTKEGGMLAYYEICDFLRGATVHRILGQQVPYATKGNQWVGYDDQESVKSKVQY
LKDRQLAGAMVWALDLDDFQGSFCGQDLRFPLTNAIKDALAAT

Chimera 22

YKLVCYYTSWSQYREGDGSCFPDALDRFLCTHIIYSFANISNDHIDTWEWNDVTLYGMLNTLKN
RNPNLKTLLSVGGWNFGSQRFSKIASNTQSRRTFIKSVPPFLRTHGFDGLDLDWEYPGRRDKQH
FTTLIKEMKAEFIKEAQPGKKQLLLSAALSAGKVTIDSSYDIAKISQHLDFISIMTYDFHGAWR
GTTGHHSPLFRGQEDASPDRFSNTDYAVGYMLRLGAPASKLVMGIPTFGRSFTLASSETGVGAP
ISGPGIPGRFTKEAGTLAYYEVCSWKGATKQRIQDQKVPYIFRDNQWVGFDDVESFKTKVQYLK
DRQLAGAMVWALDLDDFQGSFCGQDLRFPLTNAIKDALAAT

Chimera 23

YKLVCYYTSWSQYREGDGSCFPDALDRFLCTHIIYSFANISNDHIDTWEWNDVTLYGMLNTLKN
RNPNLKTLLSVGGWNFGSQRFSKIASNTQSRRTFIKSVPPFLRTHGFDGLDLDWEYPGRRDKQH
FTTLIKEMKAEFIKEAQPGKKQLLLSAALSAGKVTIDSSYDIAKISQHLDFISIMTYDFHGAWR
GTTGHHSPLFRGQEDASPDRFSNTDYAVGYMLRLGAPASKLVMGIPTFGRSFTLASSETGVGAP
ISGPGIPGRFTKEAGTLAYYEICDFLRGATVHRILGQQVPYATKGNQWVGYDDQESVKSKVSYL
KQKGLGGAMVWALDLDDFAGFSCNQGRYPLIQTLRQELSLP

CHIT CatD T69I

AKLVCYFTNWAQYRQGEARFLPKDLDPSLCTHLIYAFAGMTNHQLSTIEWNDETLYQEFNGLKK
MNPKLKTLLAIGGWNFSTQKFTDMVATANNRQTFVNSAIRFLRKYSFDGLDLDWEYPGSQGSPA
VDKERFTTLVQDLANAFQQEAQTSGKERLLLSAAVPAGQTYVDAGYEVDKIAQNLDFVNLMAYD
FHGSWEKVTGHNSPLYKRQEESGAAASLNVDAAVQQWLQKGTPASKLILGMPTYGRSFTLASSS
DTRVGAPATGSGTPGPFTKEGGMLAYYEVCSWKGATKQRIQDQKVPYIFRDNQWVGFDDVESFK
TKVSYLKQKGLGGAMVWALDLDDFAGFSCNQGRYPLIQTLRQELSLP

CHIT CatD T69V

AKLVCYFTNWAQYRQGEARFLPKDLDPSLCTHLIYAFAGMTNHQLSTVEWNDETLYQEFNGLKK
MNPKLKTLLAIGGWNFSTQKFTDMVATANNRQTFVNSAIRFLRKYSFDGLDLDWEYPGSQGSPA
VDKERFTTLVQDLANAFQQEAQTSGKERLLLSAAVPAGQTYVDAGYEVDKIAQNLDFVNLMAYD
FHGSWEKVTGHNSPLYKRQEESGAAASLNVDAAVQQWLQKGTPASKLILGMPTYGRSFTLASSS
DTRVGAPATGSGTPGPFTKEGGMLAYYEVCSWKGATKQRIQDQKVPYIFRDNQWVGFDDVESFK
TKVSYLKQKGLGGAMVWALDLDDFAGFSCNQGRYPLIQTLRQELSLP

CHIT CatD T69L

AKLVCYFTNWAQYRQGEARFLPKDLDPSLCTHLIYAFAGMTNHQLSTLEWNDETLYQEFNGLKK
MNPKLKTLLAIGGWNFSTQKFTDMVATANNRQTFVNSAIRFLRKYSFDGLDLDWEYPGSQGSPA
VDKERFTTLVQDLANAFQQEAQTSGKERLLLSAAVPAGQTYVDAGYEVDKIAQNLDFVNLMAYD
FHGSWEKVTGHNSPLYKRQEESGAAASLNVDAAVQQWLQKGTPASKLILGMPTYGRSFTLASSS
DTRVGAPATGSGTPGPFTKEGGMLAYYEVCSWKGATKQRIQDQKVPYIFRDNQWVGFDDVESFK
TKVSYLKQKGLGGAMVWALDLDDFAGFSCNQGRYPLIQTLRQELSLP

CHIT CatD M61I

AKLVCYFTNWAQYRQGEARFLPKDLDPSLCTHLIYAFAGITNHQLSTTEWNDETLYQEFNGLKK
MNPKLKTLLAIGGWNFSTQKFTDMVATANNRQTFVNSAIRFLRKYSFDGLDLDWEYPGSQGSPA
VDKERFTTLVQDLANAFQQEAQTSGKERLLLSAAVPAGQTYVDAGYEVDKIAQNLDFVNLMAYD
FHGSWEKVTGHNSPLYKRQEESGAAASLNVDAAVQQWLQKGTPASKLILGMPTYGRSFTLASSS
DTRVGAPATGSGTPGPFTKEGGMLAYYEVCSWKGATKQRIQDQKVPYIFRDNQWVGFDDVESFK
TKVSYLKQKGLGGAMVWALDLDDFAGFSCNQGRYPLIQTLRQELSLP

CHIT CatD T69W

AKLVCYFTNWAQYRQGEARFLPKDLDPSLCTHLIYAFAGMTNHQLSTWEWNDETLYQEFNGLKK
MNPKLKTLLAIGGWNFSTQKFTDMVATANNRQTFVNSAIRFLRKYSFDGLDLDWEYPGSQGSPA
VDKERFTTLVQDLANAFQQEAQTSGKERLLLSAAVPAGQTYVDAGYEVDKIAQNLDFVNLMAYD
FHGSWEKVTGHNSPLYKRQEESGAAASLNVDAAVQQWLQKGTPASKLILGMPTYGRSFTLASSS
DTRVGAPATGSGTPGPFTKEGGMLAYYEVCSWKGATKQRIQDQKVPYIFRDNQWVGFDDVESFK
TKVSYLKQKGLGGAMVWALDLDDFAGFSCNQGRYPLIQTLRQELSLP

CHIT CatD M61I, T69W

AKLVCYFTNWAQYRQGEARFLPKDLDPSLCTHLIYAFAGITNHQLSTWEWNDETLYQEFNGLKK
MNPKLKTLLAIGGWNFSTQKFTDMVATANNRQTFVNSAIRFLRKYSFDGLDLDWEYPGSQGSPA
VDKERFTTLVQDLANAFQQEAQTSGKERLLLSAAVPAGQTYVDAGYEVDKIAQNLDFVNLMAYD
FHGSWEKVTGHNSPLYKRQEESGAAASLNVDAAVQQWLQKGTPASKLILGMPTYGRSFTLASSS
DTRVGAPATGSGTPGPFTKEGGMLAYYEVCSWKGATKQRIQDQKVPYIFRDNQWVGFDDVESFK
TKVSYLKQKGLGGAMVWALDLDDFAGFSCNQGRYPLIQTLRQELSLP

MT-YKL-40 I61M

YKLVCYYTSWSQYREGDGSCFPDALDRFLCTHIIYSFANMSNDHIDTWEWNDVTLYGMLNTLKN
RNPNLKTLLSVGGWNFGSQRFSKIASNTQSRRTFIKSVPPFLRTHGFDGLDLDWEYPGRRDKQH
FTTLIKEMKAEFIKEAQPGKKQLLLSAALSAGKVTIDSSYDIAKISQHLDFISIMTYDFHGAWR
GTTGHHSPLFRGQEDASPDRFSNTDYAVGYMLRLGAPASKLVMGIPTFGRSFTLASSETGVGAP
ISGPGIPGRFTKEAGTLAYYEICDFLRGATVHRILGQQVPYATKGNQWVGYDDQESVKSKVQYL
KDRQLAGAMVWALDLDDFQGSFCGQDLRFPLTNAIKDALAAT

MT-YKL-40 W69T

YKLVCYYTSWSQYREGDGSCFPDALDRFLCTHIIYSFANISNDHIDTTEWNDVTLYGMLNTLKN
RNPNLKTLLSVGGWNFGSQRFSKIASNTQSRRTFIKSVPPFLRTHGFDGLDLDWEYPGRRDKQH
FTTLIKEMKAEFIKEAQPGKKQLLLSAALSAGKVTIDSSYDIAKISQHLDFISIMTYDFHGAWR
GTTGHHSPLFRGQEDASPDRFSNTDYAVGYMLRLGAPASKLVMGIPTFGRSFTLASSETGVGAP
ISGPGIPGRFTKEAGTLAYYEICDFLRGATVHRILGQQVPYATKGNQWVGYDDQESVKSKVQYL
KDRQLAGAMVWALDLDDFQGSFCGQDLRFPLTNAIKDALAAT

MT-YKL-40 I61M, W69T

YKLVCYYTSWSQYREGDGSCFPDALDRFLCTHIIYSFANMSNDHIDTTEWNDVTLYGMLNTLKN
RNPNLKTLLSVGGWNFGSQRFSKIASNTQSRRTFIKSVPPFLRTHGFDGLDLDWEYPGRRDKQH
FTTLIKEMKAEFIKEAQPGKKQLLLSAALSAGKVTIDSSYDIAKISQHLDFISIMTYDFHGAWR
GTTGHHSPLFRGQEDASPDRFSNTDYAVGYMLRLGAPASKLVMGIPTFGRSFTLASSETGVGAP
ISGPGIPGRFTKEAGTLAYYEICDFLRGATVHRILGQQVPYATKGNQWVGYDDQESVKSKVQYL
KDRQLAGAMVWALDLDDFQGSFCGQDLRFPLTNAIKDALAAT

Supplementary Fig. S3. Deduced amino acid sequences of the recombinant YKL-40 chimeras and mutant proteins expressed in *E. coli.* The amino acid sequences are color coded, consistent with Fig. 2A, 2C, 3A, 4A, 5A, 6A and 6D. Pink, CHIT1 CatD; Light blue, YKL-40; Light green, 26th Cys and 69th Thr mutated.

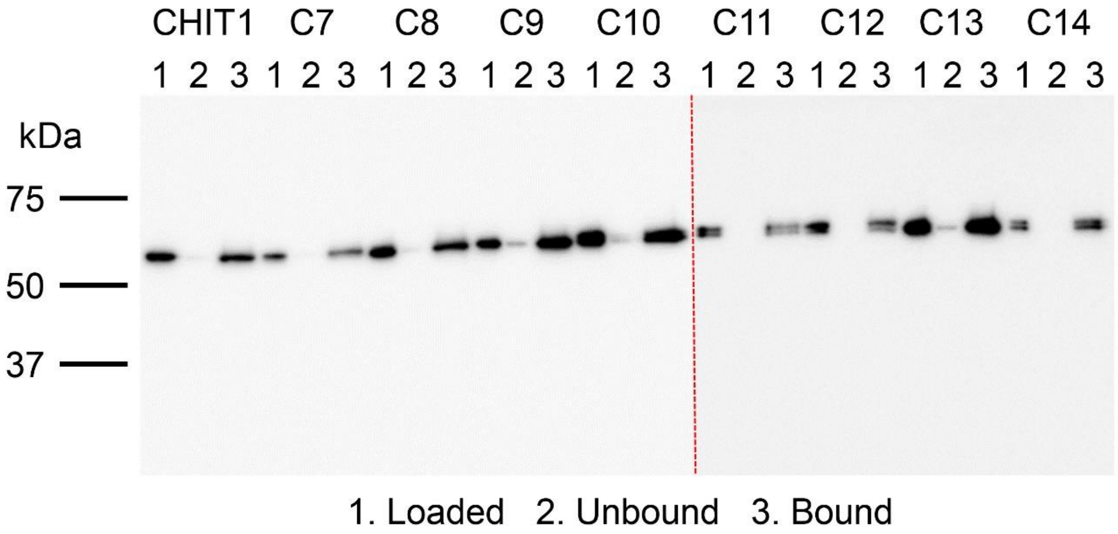

Supplementary Fig. S4. Result of binding experiments of chimeras C7-C14 to chitin substrates. Binding analysis of recombinant proteins to chitin beads. The results were obtained under pH 7.0 conditions. Western blot analyzed bound proteins. Lane 1, loaded sample; lane 2, unbound fraction; lane 3, bound fraction. The figure was assembled from two separate gels due to the high number of samples. The splice sites are indicated by red dashed lines.

Mus_musculus	VRFFPRDVDPNLCTHVIFAFAGMDNHQLSTVEHNDELLYQELNSLKTKNPKLKTLLAVGG	98
Sus_scrofa	ARFLPKDVDPNLCTHLIYAFAGMNNHQLSSTEWNDEALYKDFNGLKKMNPKLKTLLAIGG	98
Otolemur_garnettii	ARFLPKDVDPSLCTHLIYAFAGMNNHQLSTIEWNDEALYQEFNGLKKMNPKLKTLLALGG	98
Microcebus_murinus	ARFLPKDVDPNLCTHLVYAFAGMNNHQLSTLEWNDEAHYQQFNGLKKMNPKLKTLLAVGG	98
Lemur_catta	ARFLPKDVNPDLCTHLVYAFAGMNNLQLSTTEWSDEALYQQFNGLKKMNPRLKTLLAIGG	98
Propithecus_coquereli	ARFLPKDVNPNLCTHLVYAFAGMNNHQLSTIEWNDEALYQAFNGLKKMNPKLKTLLAIGG	98
Galeopterus_variegatus	ARFLPRDVDASLCTHLIYAFAGMSHHQLSSVEWNDEALYQEFNGLKQMNPKLKTLLAIGG	84
Carlito_syrichta	ARFLPKDVDPNLCTHLIYAFAGMNNHQLNTIEWNDEVLYQEFNGLKRTNPKLKTLLAIGG	98
Aotus_nancymaae	ARFLPKDVDPSLCTHLIYAFAGMTSHQLSTTELNDETLYQEFNGLKKMNPKLKTLLAIGG	96
Callithrix_jacchus	ARFLPKDVDPSLCTHLIYAFAGMTSHQLSTTEWNDETLYQEFNGLKKMNPKLKTLLAIGG	96
Saimiri_boliviensis	ARFLPKDVDPGLCTHLIYAFAGMTSHQLSTIEWNDETLYQEFNSLKKMNPNLKTLLAIGG	96
Sapajus_apella	ARFLPKDVDPSLCTHLIYAFAGMTNHQLSTTEWNDETLYQEFNSLKKMNPKLKTLLAIGG	96
Papio_anubis	ARFLPKDVDPSLCTHLIYAFAGMTNHQLSTIEWNDETLYQEFNGLKKMNPKLKTLLAIGG	113
Theropithecus_gelada	ARFLPKDVDPSLCTHLIYAFAGMTNHQLSTIEWNDETLYQEFNGLKKMNPKLKTLLAIGG	98
Chlorocebus_sabaeus	ARFLPKDVDPSLCTHLIYAFAGMTNHQLSTIEWNDETLYQEFNGLKKMNPKLKTLLAIGG	98
Mandrillus_leucophaeus	ARFMPKDVDPSLCTHLIYAFAGMTNHQLSTIEWNDETLYQEFNGLKKMNPKLKTLLAIGG	98
Cercocebus_atys	ARFLPKDVDPSLCTHLIYAFAGMTNHQLSTIEWNDETLYQEFNGRKKMNPKLKTLLAIGG	98
Macaca_mulatta	ARFLPKDVDPSLCTHLIYAFAGMTNHQLSTIEWNDETLYQEFNGRKKMNPKLKTLLAIGG	98
Macaca_fascicularis	ARFLPKDVDPSLCTHLIYAFAGMTNHQLSTIEWNDETLYQEFNGRKKMNPKLKTLLAIGG	98
Colobus_angolensis	ARFLPKDVDPSLCTHLIYAFAGMTNHQLSTIEWNDETLYQEFNGLKKMNPKLKTLLAIGG	98
Piliocolobus_tephrosceles	ARFLPKDVDPSLCTHLIYAFAGMTNHQLSTIEWNDETLYQEFNGLKKMNPKLKTLLAIGG	98
Rhinopithecus_roxellana	ARFLPKDVDPSLCTHLIYAFADMTNHQLSTIEWNDETLYQEFNGLKKMNPKLKTLLAIGG	98
Trachypithecus_francoisi	ARFLPKDVDPSLCTHLIYAFAGMTNHQLSTVEWNDETLYQEFNGLKKMNPKLKTLLAIGG	98
Pan_troglodytes	ARFLPKDVDPSLCTHLIYAFAGMTNHQLSTIEWNDETLYQEFNGLKKMNPKLKTLLAIGG	113
Homo_sapiens	ARFLPKDLDPSLCTHLIYAFAGMTNHQLSTTEWNDETLYQEFNGLKKMNPKLKTLLAIGG	98
Pan_paniscus	ARFLPKDVDPSLCTHLIYAFAGMTNHQLSTIEWNDETLYQEFNGLKKMNPKLKTLLAIGG	108
Gorilla_gorilla	ARFLPKDVDPSLCTHLIYAFAGMTNHQLSTIEWNDETLYQEFNGLKKMNPKLKTLLAIGG	108
Hylobates_moloch	ARFLPKDVDPSLCTHLIYAFAGMTNHQLSTIEWNDETLYQEFNGLKKMNPKLKTLLAIGG	108
Nomascus_leucogenys	ARFLPKDVDPSLCTHLIYAFAGMTNHQLSTIEWNDETLYQEFNGLKKMNPKLKTLLAIGG	108
Pongo_abelii	ARFLPKDVDPSLCTHLIYAFAGMTNHQLSTIEWNDETLYQEFNGLKKMNPKLKTLLAIGG	108

Supplementary Fig. S5. Comparison of the 69th amino acid in exon 3 of CHIT1 CatD among primates. In humans, this amino acid is threonine (T, pink), but in other primates, it is sometimes replaced with isoleucine (I, green), valine (V, blue), or leucine (L, yellow).

Supplementary Table S1. Signal levels of each recombinant protein on Western blots relative to CHIT1 CatD.

Sample	Signal level (%)
CHIT1 CatD	100
WT-YKL-40	118.8 ± 10.2
MT-YKL-40	89.8 ± 26.9
Chimera 1	88.9 ± 17.5
Chimera 2	80.9 ± 21.9
Chimera 3	98.1 ± 30.8
Chimera 4	86.3 ± 28.5
Chimera 5	85.8 ± 22.4
Chimera 6	125.8 ± 32.3
Chimera 7	137.1 ± 39.6
Chimera 8	121.2 ± 19.8
Chimera 9	98.7 ± 24.0
Chimera 10	103.6 ± 8.7
Chimera 11	88.2 ± 15.4
Chimera 12	97.1 ± 28.6
Chimera 13	103 ± 9.3
Chimera 14	87.2 ± 20.4
Chimera 15	80.7 ± 31.2
MT-YKL-40 C26S	92.2 ± 21.5
CHIT1 CatD C26S	85.4 ± 12.4
C15 C26S	120.7 ± 11.5
Chimera 16	121.3 ± 20.1
Chimera 17	131.1 ± 12.9
Chimera 18	95.2 ± 28.0
Chimera 19	111.3 ± 12.1
Chimera 20	102.7 ± 19.6
Chimera 21	89.3 ± 14.3
Chimera 22	117.4 ± 11.2
Chimera 23	81.5 ± 9.9
CHIT1 T69I	111.5 ± 17.3
CHIT1 T69V	94.2 ± 13.6

CHIT1 T69L	104.1 ± 19.3
CHIT1 M61I	106.3 ± 33.0
CHIT1 T69W	115.1 ± 11.2
CHIT1 M61I, T69W	121.6 ± 31.6
MT I61M	113.2 ± 26.9
MT W69T	92.5 ± 9.8
MT I61M, W69T	83.5 ± 17.4

Supplementary Table S2. Combination of primer and template to prepare each chimera by PCR.

Product name	Template DNA	Primer name	Sequence (5' - 3')
WT-YKL-40	YKL-40 cDNA	YKL-40_FW61	CCCTGCTCTGCTGCAGCCAGA ATGGG
		YKL-40_RV1257	CCGTGCTGTGTGCAGAACAGA GGG

MT-YKL-40 product 1	WT-YKL-40	pEZZ18_Fw2533	CCGTGCTGTGTGCAGAACAGA GGG
		YKL_Mut_Rv	GTCCAGGGTATTCCCAGTCAA GGTCCAGCCCATCAAAGCC
MT-YKL-40 product 2	WT-YKL-40	YKL_Mut_Fw	CTGGACCTTGACTGGGAATAC CCTGGACGGAGAGACAAAC
		Sal_BGH_Rv	AGGGGTCGACTAGAAGGCACA GTCGAGGCTGATCA
↓			
MT-YKL-40	MT-YKL-40 product 1+2	pEZZ18_Fw2533	CCGTGCTGTGTGCAGAACAGA GGG
		Sal_BGH_Rv	AGGGGTCGACTAGAAGGCACA GTCGAGGCTGATCA

Chimera 1 Product 1	MT-YKL-40	pEZZ18_Fw2533	CCGTGCTGTGTGCAGAACAGA GGG
		YKL_C1_Rv	CACTGTTGCACAGCAGCATCA GTGTTGCTGAATCTGTCAG
Chimera 1 Product 2	CHIT1 CatD	YKL_C1_Fw	CTGACAGATTCAGCAACACTG ATGCTGCTGTGCAACAGTG
		Sal_BGH_Rv	AGGGGTCGACTAGAAGGCACA GTCGAGGCTGATCA
↓			
Chimera 1	Chimera 1 Product 1+2	pEZZ18_Fw2533	CCGTGCTGTGTGCAGAACAGA GGG

		Sal_BGH_Rv	AGGGGTCGACTAGAAGGCACAGTCGAGGCTGATCA

Chimera 2 Product 1	MT-YKL-40	pEZZ18_Fw2533	CCGTGCTGTGTGCAGAACAGAGGG
		YKL_C2_Rv	GTGGCTACCATATCTGTGAATCTTTGAGACCCAAAGTTCC
Chimera 2 Product 2	CHIT1 CatD	YKL_C2_Fw	GGAACTTTGGGTCTCAAAGATTCACAGATATGGTAGCCAC
		Sal_BGH_Rv	AGGGGTCGACTAGAAGGCACAGTCGAGGCTGATCA
↓			
Chimera 2	Chimera 2 Product 1+2	pEZZ18_Fw2533	CCGTGCTGTGTGCAGAACAGAGGG
		Sal_BGH_Rv	AGGGGTCGACTAGAAGGCACAGTCGAGGCTGATCA

Chimera 3 Product 1	CHIT1 CatD	pEZZ18_Fw2533	CCGTGCTGTGTGCAGAACAGAGGG
		YKL_C3_Rv	ATGTACCCCACAGCATAGTCCACGTTGAGGCTGGCTGCTG
Chimera 3 Product 2	MT-YKL-40	YKL_C3_Fw	CAGCAGCCAGCCTCAACGTGGACTATGCTGTGGGGTACAT
		Sal_BGH_Rv	AGGGGTCGACTAGAAGGCACAGTCGAGGCTGATCA
↓			
Chimera 3	Chimera 3 Product 1+2	pEZZ18_Fw2533	CCGTGCTGTGTGCAGAACAGAGGG
		Sal_BGH_Rv	AGGGGTCGACTAGAAGGCACAGTCGAGGCTGATCA

Chimera 4 Product 1	CHIT1 CatD	pEZZ18_Fw2533	CCGTGCTGTGTGCAGAACAGAGGG
		YKL_C4_Rv	TTGGAGGCTATCTTGGAAAACTTCTGAGTGCTGAAATTCC

Chimera 4 Product 2	MT-YKL-40	YKL_C4_Fw	GGAATTTCAGCACTCAGAAGTTTTCCAAGATAGCCTCCAA
		Sal_BGH_Rv	AGGGGTCGACTAGAAGGCACAGTCGAGGCTGATCA
↓			
Chimera 4	Chimera 4 Product 1+2	pEZZ18_Fw2533	CCGTGCTGTGTGCAGAACAGAGGG
		Sal_BGH_Rv	AGGGGTCGACTAGAAGGCACAGTCGAGGCTGATCA

Chimera 5 Product 1	Chimera 2	pEZZ18_Fw2533	CCGTGCTGTGTGCAGAACAGAGGG
		YKL_C3_Rv	ATGTACCCCACAGCATAGTCCACGTTGAGGCTGGCTGCTG
Chimera 5 Product 2	MT-YKL-40	YKL_C3_Fw	CAGCAGCCAGCCTCAACGTGGACTATGCTGTGGGGTACAT
		Sal_BGH_Rv	AGGGGTCGACTAGAAGGCACAGTCGAGGCTGATCA
↓			
Chimera 5	Chimera 5 Product 1+2	pEZZ18_Fw2533	CCGTGCTGTGTGCAGAACAGAGGG
		Sal_BGH_Rv	AGGGGTCGACTAGAAGGCACAGTCGAGGCTGATCA

Chimera 6 Product 1	Chimera 4	pEZZ18_Fw2533	CCGTGCTGTGTGCAGAACAGAGGG
		YKL_C1_Rv	CACTGTTGCACAGCAGCATCAGTGTTGCTGAATCTGTCAG
Chimera 6 Product 2	CHIT1 CatD	YKL_C1_Fw	CTGACAGATTCAGCAACACTGATGCTGCTGTGCAACAGTG
		Sal_BGH_Rv	AGGGGTCGACTAGAAGGCACAGTCGAGGCTGATCA
↓			
Chimera 6	Chimera 6 Product 1+2	pEZZ18_Fw2533	CCGTGCTGTGTGCAGAACAGAGGG

		Sal_BGH_Rv	AGGGGTCGACTAGAAGGCACA GTCGAGGCTGATCA

Chimera 7 Product 1	MT-YKL-40	pEZZ18_Fw2533	CCGTGCTGTGTGCAGAACAGA GGG
		YKL_C7_Rv	AGGGTCTTCAGCTTGGGATTC CTGTTCTTGAGTGTGTTGA
Chimera 7 Product 2	CHIT1 CatD	YKL_C7_Fw	TCAACACACTCAAGAACAGGA ATCCCAAGCTGAAGACCCT
		Sal_BGH_Rv	AGGGGTCGACTAGAAGGCACA GTCGAGGCTGATCA
↓			
Chimera 7	Chimera 7 Product 1+2	pEZZ18_Fw2533	CCGTGCTGTGTGCAGAACAGA GGG
		Sal_BGH_Rv	AGGGGTCGACTAGAAGGCACA GTCGAGGCTGATCA

Chimera 8 Product 1	CHIT1 CatD	pEZZ18_Fw2533	CCGTGCTGTGTGCAGAACAGA GGG
		YKL_C8_Rv	AGAGTCTTCAGGTTGGGGTTC ATCTTCTTCAGGCCATTGA
Chimera 8 Product 2	Chimera 2	YKL_C8_Fw	TCAATGGCCTGAAGAAGATGA ACCCCAACCTGAAGACTCT
		Sal_BGH_Rv	AGGGGTCGACTAGAAGGCACA GTCGAGGCTGATCA
↓			
Chimera 8	Chimera 8 Product 1+2	pEZZ18_Fw2533	CCGTGCTGTGTGCAGAACAGA GGG
		Sal_BGH_Rv	AGGGGTCGACTAGAAGGCACA GTCGAGGCTGATCA

Chimera 9 Product 1	Chimera 4	pEZZ18_Fw2533	CCGTGCTGTGTGCAGAACAGA GGG
		YKL_C9_Rv	TGGAAGGCATTGGCCAAGTCC TTGATTAGGGTGGTAAAAT

Chimera 9 Product 2	CHIT1 CatD	YKL_C9_Fw	ATTTTACCACCCTAATCAAGGACTTGGCCAATGCCTTCCA
		Sal_BGH_Rv	AGGGGTCGACTAGAAGGCACAGTCGAGGCTGATCA
↓			
Chimera 9	Chimera 9 Product 1+2	pEZZ18_Fw2533	CCGTGCTGTGTGCAGAACAGAGGG
		Sal_BGH_Rv	AGGGGTCGACTAGAAGGCACAGTCGAGGCTGATCA

Chimera 10 Product 1	CHIT1 CatD	pEZZ18_Fw2533	CCGTGCTGTGTGCAGAACAGAGGG
		YKL_C10_Rv1	ATAAATTCGGCCTTCATTTCCTGTACCAGGGTTGTGAAGC
Chimera 10 Product 2	MT-YKL-40	YKL_C10_Fw1	GCTTCACAACCCTGGTACAGGAAATGAAGGCCGAATTTAT
		YKL_C10_Rv2	AGGTTGACAAAATCCAGGTTTTGGGATATCTTGGCAATGT
Chimera 10 Product 3	CHIT1 CatD	YKL_C10_Fw2	ACATTGCCAAGATATCCCAAAACCTGGATTTTGTCAACCT
		Sal_BGH_Rv	AGGGGTCGACTAGAAGGCACAGTCGAGGCTGATCA
↓			
Chimera 10	Chimera 10 Product 1+2+3	pEZZ18_Fw2533	CCGTGCTGTGTGCAGAACAGAGGG
		Sal_BGH_Rv	AGGGGTCGACTAGAAGGCACAGTCGAGGCTGATCA

Chimera 11 Product 1	CHIT1 CatD	pEZZ18_Fw2533	CCGTGCTGTGTGCAGAACAGAGGG
		YKL_C11_Rv	ATGCTAATGAAATCCAGGTGCTGGGCGATTTTGTCCACCT
Chimera 11 Product 2	Chimera 1	YKL_C11_Fw	AGGTGGACAAAATCGCCCAGCACCTGGATTTCATTAGCAT

		Sal_BGH_Rv	AGGGGTCGACTAGAAGGCACA GTCGAGGCTGATCA
↓			
Chimera 11	Chimera 11 Product 1+2	pEZZ18_Fw2533	CCGTGCTGTGTGCAGAACAGA GGG
		Sal_BGH_Rv	AGGGGTCGACTAGAAGGCACA GTCGAGGCTGATCA

Chimera 12 Product 1	Chimera 3	pEZZ18_Fw2533	CCGTGCTGTGTGCAGAACAGA GGG
		YKL_C12_Rv	GCCCCCTTCCAGGAGCAGACC TCATAGTAGGCAAGGGTCC
Chimera 12 Product 2	CHIT1 CatD	YKL_C12_Fw	GGACCCTTGCCTACTATGAGG TCTGCTCCTGGAAGGGGGC
		Sal_BGH_Rv	AGGGGTCGACTAGAAGGCACA GTCGAGGCTGATCA
↓			
Chimera 12	Chimera 12 Product 1+2	pEZZ18_Fw2533	CCGTGCTGTGTGCAGAACAGA GGG
		Sal_BGH_Rv	AGGGGTCGACTAGAAGGCACA GTCGAGGCTGATCA

Chimera 13 Product 1	CHIT1 CatD	pEZZ18_Fw2533	CCGTGCTGTGTGCAGAACAGA GGG
		YKL_C13_Rv1	CCGCGGAGGAAGTCACAGATT TCATAGTAGGCCAGCATCC
Chimera 13 Product 2	MT-YKL-40	YKL_C13_Fw1	GGATGCTGGCCTACTATGAAA TCTGTGACTTCCTCCGCGG
		YKL_C13_Rv2	TTCTGCTTCAGATAGCTGACCT TGCTTTTGACGCTTTCCT
Chimera 13 Product 3	CHIT1 CatD	YKL_C13_Fw2	AGGAAAGCGTCAAAAGCAAGG TCAGCTATCTGAAGCAGAA
		Sal_BGH_Rv	AGGGGTCGACTAGAAGGCACA GTCGAGGCTGATCA
↓			

Chimera 13	Chimera 13 Product 1+2+3	pEZZ18_Fw2533	CCGTGCTGTGTGCAGAACAGAGGG
		Sal_BGH_Rv	AGGGGTCGACTAGAAGGCACAGTCGAGGCTGATCA

Chimera 14 Product 1	CHIT1 CatD	pEZZ18_Fw2533	CCGTGCTGTGTGCAGAACAGAGGG
		YKL_C14_Rv	CTGTCCTTCAGGTACTGCACCTTGGTTTTGAAGCTCTCCA
Chimera 14 Product 2	MT-YKL-40	YKL_C14_Fw	TGGAGAGCTTCAAAACCAAGGTGCAGTACCTGAAGGACAG
		Sal_BGH_Rv	AGGGGTCGACTAGAAGGCACAGTCGAGGCTGATCA
↓			
Chimera 14	Chimera 14 Product 1+2	pEZZ18_Fw2533	CCGTGCTGTGTGCAGAACAGAGGG
		Sal_BGH_Rv	AGGGGTCGACTAGAAGGCACAGTCGAGGCTGATCA

Chimera 15 Product 1	CHIT1 CatD	pEZZ18_Fw2533	CCGTGCTGTGTGCAGAACAGAGGG
		YKL_C8_Rv	AGAGTCTTCAGGTTGGGGTTCATCTTCTTCAGGCCATTGA
Chimera 15 Product 2	MT-YKL-40	YKL_C8_Fw	TCAATGGCCTGAAGAAGATGAACCCCAACCTGAAGACTCT
		YKL_C9_Rv	TGGAAGGCATTGGCCAAGTCCTTGATTAGGGTGGTAAAAT
Chimera 15 Product 3	Chimera 11	YKL_C9_Fw	ATTTTACCACCCTAATCAAGGACTTGGCCAATGCCTTCCA
		YKL_C13_Rv2	TTCTGCTTCAGATAGCTGACCTTGCTTTTGACGCTTTCCT
Chimera 15 Product 4	MT-YKL-40	YKL_C13_Fw2	AGGAAAGCGTCAAAAGCAAGGTCAGCTATCTGAAGCAGAA
		Sal_BGH_Rv	AGGGGTCGACTAGAAGGCACAGTCGAGGCTGATCA

↓			
Chimera 15	Chimera 15 Product 1+2+3+4	pEZZ18_Fw2533	CCGTGCTGTGTGCAGAACAGAGGG
		Sal_BGH_Rv	AGGGGTCGACTAGAAGGCACAGTCGAGGCTGATCA

MT-YKL-40 C26S product 1	MT-YKL-40	pEZZ18_Fw2533	CCGTGCTGTGTGCAGAACAGAGGG
		YKL_C26S_Rv	TGGTGTAGTAGCTGACCAGTTTGTAAGGATCCCCGGGTAC
MT-YKL-40 C26S product 2	MT-YKL-40	YKL_C26S_Fw	CAAACTGGTCAGCTACTACACCAGCTGGTCCCAGTACCGG
		Sal_BGH_Rv	AGGGGTCGACTAGAAGGCACAGTCGAGGCTGATCA
↓			
MT-YKL-40 C26S	MT-YKL-40 C26S product 1+2	pEZZ18_Fw2533	CCGTGCTGTGTGCAGAACAGAGGG
		Sal_BGH_Rv	AGGGGTCGACTAGAAGGCACAGTCGAGGCTGATCA

CHIT1 CatD C26S product 1	CHIT1 CatD	pEZZ18_Fw2533	CCGTGCTGTGTGCAGAACAGAGGG
		CHIT1_C26S_Rv	TGGTGAAGTAGCTGACCAGTTTTGCAGGATCCCCGGGTAC
CHIT1 CatD C26S product 2	CHIT1 CatD	CHIT1_C26S_Fw	AAAACTGGTCAGCTACTTCACCAACTGGGCCCAGTACAGA
		Sal_BGH_Rv	AGGGGTCGACTAGAAGGCACAGTCGAGGCTGATCA
↓			
CHIT1 CatD C26S	CHIT1 CatD C26S product 1+2	pEZZ18_Fw2533	CCGTGCTGTGTGCAGAACAGAGGG
		Sal_BGH_Rv	AGGGGTCGACTAGAAGGCACAGTCGAGGCTGATCA

C15 C26S product 1	C15	pEZZ18_Fw2533	CCGTGCTGTGTGCAGAACAGAGGG
		CHIT1_C26S_Rv	TGGTGAAGTAGCTGACCAGTTTTGCAGGATCCCCGGGTAC
C15 C26S product 2	C15	CHIT1_C26S_Fw	AAAACTGGTCAGCTACTTCACCAACTGGGCCCAGTACAGA
		Sal_BGH_Rv	AGGGGTCGACTAGAAGGCACAGTCGAGGCTGATCA
↓			
C15 C26S	C15 C26S product 1+2	pEZZ18_Fw2533	CCGTGCTGTGTGCAGAACAGAGGG
		Sal_BGH_Rv	AGGGGTCGACTAGAAGGCACAGTCGAGGCTGATCA

Chimera 16 Product 1	CHIT1 CatD	pEZZ18_Fw2533	CCGTGCTGTGTGCAGAACAGAGGG
		YKL_C8_Rv	AGAGTCTTCAGGTTGGGGTTCATCTTCTTCAGGCCATTGA
Chimera 16 Product 2	MT-YKL-40	YKL_C8_Fw	TCAATGGCCTGAAGAAGATGAACCCCAACCTGAAGACTCT
		Sal_BGH_Rv	AGGGGTCGACTAGAAGGCACAGTCGAGGCTGATCA
↓			
Chimera 16	Chimera 16 Product 1+2	pEZZ18_Fw2533	CCGTGCTGTGTGCAGAACAGAGGG
		Sal_BGH_Rv	AGGGGTCGACTAGAAGGCACAGTCGAGGCTGATCA

Chimera 17 Product 1	MT-YKL-40	pEZZ18_Fw2533	CCGTGCTGTGTGCAGAACAGAGGG
		YKL_C7_Rv	AGGGTCTTCAGCTTGGGATTCCTGTTCTTGAGTGTGTTGA
Chimera 17 Product 2	Chimera 4	YKL_C7_Fw	TCAACACACTCAAGAACAGGAATCCCAAGCTGAAGACCCT

		Sal_BGH_Rv	AGGGGTCGACTAGAAGGCACA GTCGAGGCTGATCA
↓			
Chimera 17	Chimera 17 Product 1+2	pEZZ18_Fw2533	CCGTGCTGTGTGCAGAACAGA GGG
		Sal_BGH_Rv	AGGGGTCGACTAGAAGGCACA GTCGAGGCTGATCA

Chimera 18 Product 1	Chimera 2	pEZZ18_Fw2533	CCGTGCTGTGTGCAGAACAGA GGG
		YKL_C10_Rv1	ATAAATTCGGCCTTCATTTCCT GTACCAGGGTTGTGAAGC
Chimera 18 Product 2	MT-YKL-40	YKL_C10_Fw1	GCTTCACAACCCTGGTACAGG AAATGAAGGCCGAATTTAT
		Sal_BGH_Rv	AGGGGTCGACTAGAAGGCACA GTCGAGGCTGATCA
↓			
Chimera 18	Chimera 18 Product 1+2	pEZZ18_Fw2533	CCGTGCTGTGTGCAGAACAGA GGG
		Sal_BGH_Rv	AGGGGTCGACTAGAAGGCACA GTCGAGGCTGATCA

Chimera 19 Product 1	MT-YKL-40	pEZZ18_Fw2533	CCGTGCTGTGTGCAGAACAGA GGG
		YKL_C9_Rv	TGGAAGGCATTGGCCAAGTCC TTGATTAGGGTGGTAAAAT
Chimera 19 Product 2	CHIT1 CatD	YKL_C9_Fw	ATTTTACCACCCTAATCAAGGA CTTGGCCAATGCCTTCCA
		YKL_C11_Rv	ATGCTAATGAAATCCAGGTGCT GGGCGATTTTGTCCACCT
Chimera 19 Product 3	MT-YKL-40	YKL_C11_Fw	AGGTGGACAAAATCGCCCAGC ACCTGGATTTCATTAGCAT
		Sal_BGH_Rv	AGGGGTCGACTAGAAGGCACA GTCGAGGCTGATCA
↓			

Chimera 19	Chimera 19 Product 1+2+3	pEZZ18_Fw2533	CCGTGCTGTGTGCAGAACAGAGGG
		Sal_BGH_Rv	AGGGGTCGACTAGAAGGCACAGTCGAGGCTGATCA

Chimera 20 Product 1	MT-YKL-40	pEZZ18_Fw2533	CCGTGCTGTGTGCAGAACAGAGGG
		YKL_C10_Rv2	AGGTTGACAAAATCCAGGTTTTGGGATATCTTGGCAATGT
Chimera 20 Product 2	Chimera 3	YKL_C10_Fw2	ACATTGCCAAGATATCCCAAAACCTGGATTTTGTCAACCT
		Sal_BGH_Rv	AGGGGTCGACTAGAAGGCACAGTCGAGGCTGATCA
↓			
Chimera 20	Chimera 20 Product 1+2	pEZZ18_Fw2533	CCGTGCTGTGTGCAGAACAGAGGG
		Sal_BGH_Rv	AGGGGTCGACTAGAAGGCACAGTCGAGGCTGATCA

Chimera 21 Product 1	Chimera 1	pEZZ18_Fw2533	CCGTGCTGTGTGCAGAACAGAGGG
		YKL_C13_Rv1	CCGCGGAGGAAGTCACAGATTTCATAGTAGGCCAGCATCC
Chimera 21 Product 2	MT-YKL-40	YKL_C13_Fw1	GGATGCTGGCCTACTATGAAATCTGTGACTTCCTCCGCGG
		Sal_BGH_Rv	AGGGGTCGACTAGAAGGCACAGTCGAGGCTGATCA
↓			
Chimera 21	Chimera 21 Product 1+2	pEZZ18_Fw2533	CCGTGCTGTGTGCAGAACAGAGGG
		Sal_BGH_Rv	AGGGGTCGACTAGAAGGCACAGTCGAGGCTGATCA

Chimera 22 Product 1	MT-YKL-40	pEZZ18_Fw2533	CCGTGCTGTGTGCAGAACAGAGGG

		YKL_C12_Rv	GCCCCCTTCCAGGAGCAGACC TCATAGTAGGCAAGGGTCC
Chimera 22 Product 2	CHIT1 CatD	YKL_C12_Fw	GGACCCTTGCCTACTATGAGG TCTGCTCCTGGAAGGGGGC
		YKL_C14_Rv	CTGTCCTTCAGGTACTGCACCT TGGTTTTGAAGCTCTCCA
Chimera 22 Product 3	MT-YKL-40	YKL_C14_Fw	TGGAGAGCTTCAAAACCAAGG TGCAGTACCTGAAGGACAG
		Sal_BGH_Rv	AGGGGTCGACTAGAAGGCACA GTCGAGGCTGATCA
↓			
Chimera 22	Chimera 22 Product 1+2+3	pEZZ18_Fw2533	CCGTGCTGTGTGCAGAACAGA GGG
		Sal_BGH_Rv	AGGGGTCGACTAGAAGGCACA GTCGAGGCTGATCA

Chimera 23 Product 1	MT-YKL-40	pEZZ18_Fw2533	CCGTGCTGTGTGCAGAACAGA GGG
		YKL_C13_Rv2	TTCTGCTTCAGATAGCTGACCT TGCTTTTGACGCTTTCCT
Chimera 23 Product 2	CHIT1 CatD	YKL_C13_Fw2	AGGAAAGCGTCAAAAGCAAGG TCAGCTATCTGAAGCAGAA
		Sal_BGH_Rv	AGGGGTCGACTAGAAGGCACA GTCGAGGCTGATCA
↓			
Chimera 23	Chimera 23 Product 1+2	pEZZ18_Fw2533	CCGTGCTGTGTGCAGAACAGA GGG
		Sal_BGH_Rv	AGGGGTCGACTAGAAGGCACA GTCGAGGCTGATCA

CHIT1 CatD T69I Product 1	CHIT1 CatD	pEZZ18_Fw2533	CCGTGCTGTGTGCAGAACAGA GGG
		CHIT1_T69I_Rv	CATTCCACTCAATGGTGCTCAG CTGGTGGTTGGTCA

CHIT1 CatD T69I Product 2	CHIT1 CatD	CHIT1_T69I_Fw	GCTGAGCACCATTGAGTGGAA TGACGAGACTCTCTA
		Sal_BGH_Rv	AGGGGTCGACTAGAAGGCACA GTCGAGGCTGATCA
↓			
CHIT1 CatD T69I	CHIT1 CatD T69I Product 1+2	pEZZ18_Fw2533	CCGTGCTGTGTGCAGAACAGA GGG
		Sal_BGH_Rv	AGGGGTCGACTAGAAGGCACA GTCGAGGCTGATCA

CHIT1 CatD T69V Product 1	CHIT1 CatD	pEZZ18_Fw2533	CCGTGCTGTGTGCAGAACAGA GGG
		CHIT1_T69V_Rv	CATTCCACTCAACGGTGCTCA GCTGGTGGTTGGTCA
CHIT1 CatD T69V Product 2	CHIT1 CatD	CHIT1_T69V_Fw	GCTGAGCACCGTTGAGTGGAA TGACGAGACTCTCTA
		Sal_BGH_Rv	AGGGGTCGACTAGAAGGCACA GTCGAGGCTGATCA
↓			
CHIT1 CatD T69V	CHIT1 CatD T69V Product 1+2	pEZZ18_Fw2533	CCGTGCTGTGTGCAGAACAGA GGG
		Sal_BGH_Rv	AGGGGTCGACTAGAAGGCACA GTCGAGGCTGATCA

CHIT1 CatD T69L Product 1	CHIT1 CatD	pEZZ18_Fw2533	CCGTGCTGTGTGCAGAACAGA GGG
		CHIT1_T69L_Rv	CATTCCACTCCAGGGTGCTCA GCTGGTGGTTGGTCA
CHIT1 CatD T69L Product 2	CHIT1 CatD	CHIT1_T69L_Fw	GCTGAGCACCCTGGAGTGGAA TGACGAGACTCTCTA
		Sal_BGH_Rv	AGGGGTCGACTAGAAGGCACA GTCGAGGCTGATCA
↓			
CHIT1 CatD T69L	CHIT1 CatD	pEZZ18_Fw2533	CCGTGCTGTGTGCAGAACAGA GGG

	T69L Product 1+2	Sal_BGH_Rv	AGGGGTCGACTAGAAGGCACAGTCGAGGCTGATCA

CHIT1 CatD M61I product 1	CHIT1 CatD	pEZZ18_Fw2533	CCGTGCTGTGTGCAGAACAGAGGG
		CHIT1_M61I_Rv	GGTGGTTGGTTATGCCAGCGAAGGCGTAGATGAGGTGGGT
CHIT1 CatD M61I product 2	CHIT1 CatD	CHIT1_M61I_Fw	CTTCGCTGGCATAACCAACCACCAGCTGAGCACCACTGAG
		Sal_BGH_Rv	AGGGGTCGACTAGAAGGCACAGTCGAGGCTGATCA
↓			
CHIT1 CatD M61I	CHIT1 CatD M61I product 1+2	pEZZ18_Fw2533	CCGTGCTGTGTGCAGAACAGAGGG
		Sal_BGH_Rv	AGGGGTCGACTAGAAGGCACAGTCGAGGCTGATCA

CHIT1 CatD T69W product 1	CHIT1 CatD	pEZZ18_Fw2533	CCGTGCTGTGTGCAGAACAGAGGG
		CHIT1_T69W_Rv	CATTCCACTCCCAGGTGCTCAGCTGGTGGTTGGTCATGCC
CHIT1 CatD T69W product 2	CHIT1 CatD	CHIT1_T69W_Fw	GCTGAGCACCTGGGAGTGGAATGACGAGACTCTCTACCAG
		Sal_BGH_Rv	AGGGGTCGACTAGAAGGCACAGTCGAGGCTGATCA
↓			
CHIT1 CatD T69W	CHIT1 CatD T69W product 1+2	pEZZ18_Fw2533	CCGTGCTGTGTGCAGAACAGAGGG
		Sal_BGH_Rv	AGGGGTCGACTAGAAGGCACAGTCGAGGCTGATCA

CHIT1 CatD M61I, T69W product 1	CHIT1 CatD	pEZZ18_Fw2533	CCGTGCTGTGTGCAGAACAGAGGG
		CHIT1_T69W_Rv	CATTCCACTCCCAGGTGCTCAGCTGGTGGTTGGTCATGCC

CHIT1 CatD M61I, T69W product 2	CHIT1 CatD	CHIT1_M61I, T69W_Fw	TCGCTGGCATAACCAACCACCAGCTGAGCACCTGGGAGTGGAATGACGAG
		Sal_BGH_Rv	AGGGGTCGACTAGAAGGCACAGTCGAGGCTGATCA
↓			
CHIT1 CatD M61I, T69W	CHIT1 CatD M61I, T69W product 1+2	pEZZ18_Fw2533	CCGTGCTGTGTGCAGAACAGAGGG
		Sal_BGH_Rv	AGGGGTCGACTAGAAGGCACAGTCGAGGCTGATCA

MT-YKL-40 I61M product 1	MT-YKL-40	pEZZ18_Fw2533	CCGTGCTGTGTGCAGAACAGAGGG
		YKL_I61M_Rv	GATCGTTGCTCATATTGGCAAAGCTGTAGATGATGTGGGT
MT-YKL-40 I61M product 2	MT-YKL-40	YKL_I61M_Fw	CTTTGCCAATATGAGCAACGATCACATCGACACCTGGGAG
		Sal_BGH_Rv	AGGGGTCGACTAGAAGGCACAGTCGAGGCTGATCA
↓			
MT-YKL-40 I61M	MT-YKL-40 I61M product 1+2	pEZZ18_Fw2533	CCGTGCTGTGTGCAGAACAGAGGG
		Sal_BGH_Rv	AGGGGTCGACTAGAAGGCACAGTCGAGGCTGATCA

MT-YKL-40 W69T product 1	MT-YKL-40	pEZZ18_Fw2533	CCGTGCTGTGTGCAGAACAGAGGG
		YKL_W69T_Rv	CATTCCACTCAGTGGTGTCGATGTGATCGTTGCTTATATT
MT-YKL-40 W69T product 2	MT-YKL-40	YKL_W69T_Fw	CATCGACACCACTGAGTGGAATGATGTGACGCTCTACGGC
		Sal_BGH_Rv	AGGGGTCGACTAGAAGGCACAGTCGAGGCTGATCA
↓			

MT-YKL-40 W69T	MT-YKL-40 W69T product 1+2	pEZZ18_Fw2533	CCGTGCTGTGTGCAGAACAGA GGG
		Sal_BGH_Rv	AGGGGTCGACTAGAAGGCACA GTCGAGGCTGATCA

MT-YKL-40 I61M, W69T product 1	MT-YKL-40	pEZZ18_Fw2533	CCGTGCTGTGTGCAGAACAGA GGG
		YKL_I61M_Rv	GATCGTTGCTCATATTGGCAAA GCTGTAGATGATGTGGGT
MT-YKL-40 I61M, W69T product 2	MT-YKL-40	YKL_I61M, W69T_Fw	TTGCCAATATGAGCAACGATCA CATCGACACCACTGAGTGGAA TGATGTG
		Sal_BGH_Rv	AGGGGTCGACTAGAAGGCACA GTCGAGGCTGATCA
↓			
MT-YKL-40 I61M, W69T	MT-YKL-40 I61M, W69T product 1+2	pEZZ18_Fw2533	CCGTGCTGTGTGCAGAACAGA GGG
		Sal_BGH_Rv	AGGGGTCGACTAGAAGGCACA GTCGAGGCTGATCA

Supplementary Table S3. NCBI GenBank Accession Numbers for YKL-40 (Chi3l1) and CHIT1.

Species	YKL-40 (Chi3l1)	CHIT1
Homo sapiens	NM_001276.4	NM_003465.3
Lemur catta	XM_045536282.1	XM_045536283.1
Propithecus coquereli	XM_012655925.1	XM_012655947.1
Microcebus murinus	XM_012758724.2	XM_012758734.2
Otolemur garnettii	XM_003792339.2	XM_003792338.2
Aotus nancymaae	XM_021677238.1	XM_021677312.1
Hylobates moloch	XM_032136549.1	XM_032136547.1
Papio anubis	XM_003893272.3	XM_021928432.2
Macaca mulatta	NM_001265920.1	XM_001103012.3
Theropithecus gelada	XM_025364778.1	XM_025356179.1
Macaca fascicularis	XM_005540483.3	XM_005540484.2
Chlorocebus sabaeus	XM_007988888.2	XM_007988887.2
Cercocebus atys	XM_012038091.1	XM_012038104.1
Rhinopithecus roxellana	XM_010378814.2	XM_030930511.1
Colobus angolensis	XM_011931797.1	XM_011931798.1
Carlito syrichta	XM_008060462.1	XM_008060460.1
Callithrix jacchus	XM_002760663.5	XM_002760664.5
Trachypithecus francoisi	XM_033199508.1	XM_033196987.1
Mandrillus leucophaeus	XM_011990491.1	XM_011990488.1
Pan paniscus	XM_008976607.2	XM_034943973.1
Nomascus leucogenys	XM_030818617.1	XM_003264579.2
Gorilla gorilla	XM_004028186.2	XM_004028187.3
Pan troglodytes	XM_001153636.3	XM_001155147.2
Saimiri boliviensis	XM_003938296.2	XM_003938301.3
Piliocolobus tephrosceles	XM_023186859.2	XM_023186968.2
Sapajus apella	XM_032288800.1	XM_032288808.1
Pongo abelii	XM_009238991.2	XM_024234097.1
Galeopterus variegatus	XM_008577641.1	XM_008577637.1
Mus musculus	NM_007695.4	NM_001284525.1
Rattus norvegicus	NM_001309820.1	NM_001079689.2
Sus scrofa	NM_001001540.1	XM_003130296.4

Supplementary data set 1 for evolutionary analysis in YKL-40 among primates.

>Homo_sapiens
ATGTACAAACTGGTCTGCTACTACACCAGCTGGTCCCAGTACCGGGAAGGCGATGGGAGCTGCTTCCCAG
ATGCCCTTGACCGCTTCCTCTGTACCCACATCATCTACAGCTTTGCCAATATAAGCAACGATCACATCGA
CACCTGGGAGTGGAATGATGTGACGCTCTACGGCATGCTCAACACACTCAAGAACAGGAACCCCAACCTG
AAGACTCTCTTGTCTGTCGGAGGATGGAACTTTGGGTCTCAAAGATTTTCCAAGATAGCCTCCAACACCC
AGAGTCGCCGGACTTTCATCAAGTCAGTACCGCCATTTCTGCGCACCCATGGCTTTGATGGGCTGGACCT
TGCCTGGCTCTACCCTGGACGGAGAGACAAACAGCATTTTACCACCCTAATCAAGGAAATGAAGGCCGAA
TTTATAAAGGAAGCCCAGCCAGGGAAAAAGCAGCTCCTGCTCAGCGCAGCACTGTCTGCGGGGAAGGTCA
CCATTGACAGCAGCTATGACATTGCCAAGATATCCCAACACCTGGATTTCATTAGCATCATGACCTACGA
TTTTCATGGAGCCTGGCGTGGGACCACAGGCCATCACAGTCCCCTGTTCCGAGGTCAGGAGGATGCAAGT
CCTGACAGATTCAGCAACACTGACTATGCTGTGGGGTACATGTTGAGGCTGGGGGCTCCTGCCAGTAAGC
TGGTGATGGGCATCCCCACCTTCGGGAGGAGCTTCACTCTGGCTTCTTCTGAGACTGGTGTTGGAGCCCC
AATCTCAGGACCGGGAATTCCAGGCCGGTTCACCAAGGAGGCAGGGACCCTTGCCTACTATGAGATCTGT
GACTTCCTCCGCGGAGCCACAGTCCATAGAATCCTCGGCCAGCAGGTCCCCTATGCCACCAAGGGCAACC
AGTGGGTAGGATACGACGACCAGGAAAGCGTCAAAAGCAAGGTGCAGTACCTGAAGGACAGGCAGCTGGC
GGGCGCCATGGTATGGGCCCTGGACCTGGATGACTTCCAGGGCTCCTTCTGTGGCCAGGATCTGCGCTTC
CCTCTCACCAATGCCATCAAGGATGCACTCGCTGCAACG
>Lemur_catta
ATGTACAAACTGGTCTGCTACTACACCAGCTGGTCCCAGTACCGGGAAGGTGATGGGAGCTGCTTCCCGG
ATGCCATCGACCATTCCCTCTGCACCCACGTCATCTACAGTTTTGCCAACATAAGCAACAATCAGATCGA
CACCTGGGAGTGGAATGATGTGACACTCTATGACACGCTGAACGCACTCAAGAACAGGAACCCCAACCTG
AAGACCCTCCTGTCTGTTGGGGGATGGAACTTTGGCTCTCAAAGATTTTCCCAAATAGCCTCCAACACGC
AGAGTCGCAGGACATTCGTCAGGTCGGTGCCACCGTTTCTGCGGGCGCACGGCTTTGATGGGCTGGACCT
GGCCTGGCTCTACCCCGGACGGAGAGACAAGCGGTACTTCACCACCCTGGTCAAGGAAATGAAGGCCGAA
TTTGCAAAGGAAGCTCAGCCAGGGAAAAAGCAGCTCCTGCTCAGCGCAGCCCTGTCGCCGGGGAAGGTCA
CCGTTGACAGTGGCTACGACATCCCCCAGATATCGCAACACCTGGATTTCATCAGCATCCTGACCTACGA
CTTTCATGGAGTCTGGCGCCGGACCACGGGACATCACAGCCCTCTGTTCCGAGGCCAGCAGGACGCGAGT
CCTGACAGATTCAGCAACACTGACTACGCTGTGGGGTACGTGTTGAGGCTGGGGGCTCCTGCCAGCAAGC
TGGTGATGGGCATCCCCACCTTCGGGAAGAGCTTCACGCTGGCCTCTTCTGAGACGGGTGTCGGAGCCCC
AATCTCGGGGCCAGGAATCCCAGGCCGGTACACCAAGGAGGCGGGGACTCTCGCCTACTACGAGATCTGT
GACTTCCTCCACGGAGCCACGGTCCGCAGACTCCTCGGCCAGCAGGTCCCCTATGCCACCAAGGGCAACC
AGTGGGTGGGATATGACGACCAGGAGAGCGTCAAAAGCAAGGTGCGGTACCTGAAGGACAGGCAGCTGGC
GGGCGCCATGGTGTGGGCCCTGGACCTGGATGACTTCCGCGGCTCCTTCTGCGGCCAGAATCTGCGCTTC
CCTCTTACCAACGCCATCAAGGACGCGCTTGCCGCGACC
>Propithecus_coquereli
ATGTACAAACTGGTCTGCTACTACACCAGCTGGTCCCAGTACCGGGAAGGTGATGGGAGCTGCTTCCCAG
ATGCCATCGACCATTCCCTCTGCACCCACGTCATCTACAGCTTTGCCAACATAAGCAACAATCAGATCGA
CACCTGGGAGTGGAATGATGTGACTCTCTATGACACGTTGAACGCACTCAAGAACAGAAACCCCAACCTG
AAGACCCTCCTGTCTGTTGGGGGATGGAACTTTGGCTCTCAAAGATTTTCCAAAATAGCCTCCAACACCC
AGAGTCGCAGAACTTTCATCAAGTCGGTGCCACCATTCCTGCGGGCTCATGGCTTTGATGGGCTGGACTT
AGCCTGGCTCTACCCTGGACGGAGAGACAAGCGGTATTTCACCGCCCTGATCAAGGAAATGAAGGCCGAA
TTTGCAAAGGAAGCTCAGCCAGGGAAAAAGCAGCTCCTGCTCAGCGCAGCAGTGTCCTCGGGGAAGGTCA
CCATTGACAGTGGCTACGACATCCCCCAGATATCGCAACACCTGGATTTCATCAGCCTCCTGACCTATGA
TTTTCATGGAGTCTGGCGCCGGACCACGGGCCATCACAGCCCTCTGTTCCGAGGCCAGCAGGATGCCAGT
CCTGACAGATTCAGCAATGCTGACTATGCTGTGGGGTACCTGTTGAGGCTGGGGGCTCCTGCCAGTAAGC
TAGTAATGGGCATCCCCACCTTCGGGAAGAGCTTCACTCTGGCCTCTTCTGAGACGGGTGTCGGAGCCCC
AATCTCGGGGCCGGGAATACCAGGCCAATACACCAAGGAGGCGGGGACCCTCGCCTACTATGAGATCTGT
GACTTCCTCCACGGAGCCACGGTCCGTAGAATCCTCGGCCAGCAGGTCCCGTATGCCACCAAGGGCAACC
AGTGGGTGGGATATGATGACCAGGAGAGCGTCAAAAGCAAGGTGCGGTACCTGAAGGACAGGCAGCTGGC
GGGCGCCATGGTGTGGGCCCTGGACCTGGATGACTTCCGCGGCTCCTTCTGCGGCCAGAATCTGCGCTTC
CCTCTTACCAATGCCATCAAGGATGCGCTCGCTGCCACT
>Microcebus_murinus
ATGTACAAGCTGGTCTGCTACTACACCAGCTGGTCCCAGTACCGGGAAGGCGATGGGAGCTGCTTCCCGG
ACGCCATCGGCCCTTCCCTCTGCACCCACATCATCTACAGTTTCGCCAACATAAGCGACGACCAGATCGA
CACCTGGGAGTGGAATGATGTGACGCTCTATGACATGCTGAACACACTCAAGAACAGGAACCCCAACCTG
AAGACCCTCCTGTCTGTTGGGGGATGGAGCTTCGGCTCTCAAAGATTTTCCAAAATAGCCTCCAACACCC
AGAGTCGCAGGACCTTCATCAAATCGGTGCCACCATTTCTGCGGGCTCATGGCTTTGATGGGCTGGACCT
CGCCTGGCTCTACCCCGAACGGAGAGACAAGCGGCATTTCACCGCCCTGATCAAGGAAATGAAGGCCGAA
TTTGCAAAGGAAGCTCAGCCAGGGAAACAGCAGCTCCTGCTCAGCGCGGCGGTGTCTTCGGGGAAGGTCA
CCATCGACAGTGGCTACGACATCCCCCAGATATCGCAACACCTGGATTTCATCAGCATCCTGACCTACGA
TTTTCATGGAGTCTGGCACAGGACCACTGGCCACCACAGCCCCCTGTTCCGAGGCCAGCAGGACGCCAGT
CCCGACAGGTTCAGCAATGCTGACTACGCTGTGGGCTACGTGCTGCGGCTGGGGGCTCCCGCCAGCAAGC
TGGTGATGGGCATCCCCACCTTCGGGAAGAGCTTCACTCTGGCCTCTTCGGAGACGGGGGTCGGCGCCCC

AATCTCGGGGCCGGGACTACCAGGCCGATACACCAAGGAGGCGGGGACCCTCGCCTACTATGAGATCTGT
GACTTCCTCCAAGGAGCTACGGTCCGCAGACTCCTGGGCCAGCAGGTCCCCTACGCCACCAAGGGCAACC
AGTGGGTGGGATATGACGACCAGGAGAGCGTCAAAAGCAAGGTGCGGTACCTGAAGGACAGGCAGCTGGC
GGGCGCCATGGTGTGGGCCCTGGACCTGGATGACTTCCGCGGCTCCTTCTGTGGCCAGAACCTGCGCTTC
CCTCTGACCAATGCCATCAAGGATGCGCTGGCTGCGACG
>Otolemur_garnettii
ATGTACAAACTAGTCTGCTACTACACCAGCTGGTCCCAGTACCGGGAAGGTGATGGGAGCTGCTTCCCAG
ATGCCATCGACCATTTCCTCTGTACCCACATCATCTACAGTTTTGCCAACATAAGCAACAATCAGATCGA
CACCTGGGAGTGGAATGATGTGACGCTCTATGACACGTTGAACTCACTCAAGAACAGGAACCCCAACCTG
AAGACCCTCCTGTCCGTTGGGGGATGGAACTTCGGCTCTGAAAGATTTTCCAAAATAGCCTCCAACACCC
AGAGACGCAGGACTTTCATCAAGTCGGTGGCACCATTTCTGCGGACGCATGGCTTTGATGGGCTGGACCT
AGCCTGGCTTTACCCTAGTCGGAGAGACAAGCGGCATTTCACCACCCTGATCAAGGAAATGAAGGCTGAA
TTTGCAAAAGAAGCTCAGCCAGGAAAAGAGCAGCTCCTACTCAGCGCAGCAGTGTCTTCGGGGAAAGTCA
CCATTGACAGCGGCTATGACATTGCCCAGATATCGCAACACCTGGATTTCCTTAGCCTCCTGACCTACGA
TTTTCATGGAGCCTGGCGCCAGACCACAGGACACCACAGCCCTCTGTTCCGAGGCCAGCAGGATGCGAGT
CCTGATAGATTCAGCAATGTTGACTATGCTGTGGGGTACATGTTGAGGCTGGGGGCTCCTGCCAATAAAC
TAGTGATGGGCATCCCCACCTTCGGGAAGAGCTTCACTCTGGCCTCTTCTGAGACGGGTGTCAGAGCCCC
AATCTCGGGGCCGGGAATACCAGGCCGGTTCACCAAGGAAGCAGGGACCCTCGCCTACTATGAGATCTGT
GACTTCCTCCACGGAGCCACGGTTCACAGACTCCTCGGCCAACAGGTCCCCTATGCCACCAAGGGCAACC
AATGGGTGGGATACGATGACCAGGAGAGCGTCAAAACCAAGGTGCAGTACCTGAAGAACAGGCAGCTGGC
AGGCGCCATGGTGTGGGCCCTGGACCTAGATGACTTCCGGGGCTCTTTCTGTGGCCAGAATCTGCGCTTC
CCTCTCACCAATGCCATCAAGGATGCACTTGCTGCGACT
>Aotus_nancymaae
ATGTACAAACTGGTCTGCTACTACACCAGCTGGTCCCAGTACCGGGAAGGCGATGGGAGCTGCTTCCCAG
ATGCCATTGACCGCTCCCTCTGTACCCACATCATCTACAGCTTTGCCAATATAAGCAACGATCACATCGA
CACCTGGGAGTGGAATGATGTGACGCTTTACGACACGCTCAACACACTCAAGAACAGGAACCCCAACCTG
AAGACCCTCCTGTCAGTTGGAGGATGGAACTTTGGCTCTCAAAGATTTTCCAAGATCGCCTCCAACACCC
AGAGTCGCCGGACTTTCATCCAGTCGGTGCCGCCATTTCTGCGCACCCATGGCTTTGATGGGCTGGACCT
CGCCTGGCTCTACCCTGGACGGAGAGACAAGCAGCATTTTACTACCCTAATCAAGGAAATGAAGGCCGAA
TTTGCAAAGGAAGCCCAGCAAGGGAAAGAGCAGCTCCTGCTTAGCGCAGCAGTGTCTGCGGGGAAGGTCA
CCATTGACAGCAGCTACGACATTGCCCGGATATCCCAACACCTGGATTTCATCAGCATCATGACCTATGA
CTTTCACGGAGCCTGGCGTGGGACCACAGGCCATCACAGTCCCCTGTTCCGAGGCCAGGAGGATGCAAGT
CCTGACAGATTCAGCAACGCTGACTACGCTGTGGGGTACGTGCTGAGGCTGGGGGCTCCTGCCAGTAAGC
TGGTGATGGGCATCCCCACCTTCGGGAAGAGCTTCACTCTGGCTTCTTCTGAAACTGGTGTCGGAGCCCC
AGTCTCGGGACCAGGAATTCCAGGCCGGTTCACCAAGGAGGCAGGGACCCTTGCCTACTATGAGATCTGT
GACTTCCTCCGCGGAGCCACAGTCCACAGAATCCTCGGCCAGCAGGTCCCCTATGCCACCAAGGGCAACC
AGTGGGTAGGATACGACGACCAGGAAAGTGTCAAAAGCAAGGTGCAGTACCTGAAGGACAGGCAGCTGGC
AGGCGCCATGGTGTGGGCCCTGGACCTGGATGACTTCCAGGGCTCCTTCTGCGGCCAGGATCTGCGCTTC
CCTCTCATCAATGCCATCAAGGACGCACTCGCTGCAACT
>Hylobates_moloch
ATGTACAAACTGGTCTGCTACTACACCAGCTGGTCCCAGTACCGGGAAGGCGATGGGAGCTGCTTCCCAG
ATGCCATTGACCGCTTCCTCTGTACCCACATCATCTACAGCTTTGCCAATATAAGCAACGATCACATCGA
CACCTGGGAGTGGAATGATGTGACACTCTACGGCATGCTCAACACACTCAAGAACAGGAACCCCAACCTG
AAGACTCTCCTGTCTGTCGGAGGATGGAACTTTGGCTCTCAAAGATTTTCCAAGATAGCCTCCAACACCC
AGAGTCGCCGGACTTTCATCAAGTCAGTACCGCCATTTCTGCGCACCCATGGCTTTGATGGGCTGGACCT
TGCCTGGCTTTACCCTGGACGGAGAGACAAGCAACATTTTACCACCCTGATCAAGGAAATGAAGGCCGAA
TTTGCAAAGGAAGCCCAGCCAGGGAAAAAGCAGCTCCTGCTCAGCGCAGCAGTGTCTGCGGGGAAGGTCA
CCATTGACAGCAGCTATGACATTGCCAAGATATCCCAACACCTGGATTTCATTAGCATCATGACCTACGA
TTTTCATGGAGCCTGGCGTGGGACCACAGGCCATCACAGTCCCCTGTTCCGAGGCCAGGAGGATGCAAGT
CCTGACAGATTCAGCAACACTGACTATGCTGTGGGGTACATGTTGAGGCTGGGGGCTCCTGCCAGTAAGC
TGGTGATGGGCATCCCCACCTTCGGGAAGAGCTTCACTCTGGCTTCTTCTGAGGCTGGTGTTGGAGCCCC
AGTCTCAGGACCAGGAATTCCAGGCCGGTTCACCAAGGAGGCAGGGACCCTTGCCTACTATGAGATCTGT
GACTTCCTGCGCGGAGCCACAGTCCATAGAATCCTCGGCCAGCAGGTCCCCTATGCCACCAAGGGCAACC
AGTGGGTAGGATACGACGACCGGGAAAGCATCAAAAGCAAGGTGCAGTACCTGAAGGACAGGCAGCTGGC
GGGCGCCATGGTATGGGCCCTGGACCTGGATGACTTCCAGGGCTCCTTCTGCGGCCAGGATCTGCGCTTC
CCTCTCACCAATGCCATCAAGGATGCACTCGCTGCAACT
>Papio_anubis
ATGTACAAACTGGTCTGCTACTACACCAGCTGGTCCCAGTACCGGGAAGGCGATGGGAGCTGCTTCCCAG
ATGCCATTGACCGCTTCCTCTGTACCCACGTCATCTACAGCTTTGCCAATATAAGCAACGATCACATCGA
CACCTGGGAGTGGAATGATGTGACACTCTACGGCATGCTCAACACACTCAAGAACAGGAACCCCAATCTG
AAGACGCTCCTGTCTGTCGGAGGATGGAACTTTGGCTCTCAAAGATTTTCCAAGATGGCCTCCAACACCC
AGAGTCGCCAGACTTTCATCAAGTCAGTACCGCCATTTCTGCGCACCCACGGCTTTGATGGGTTGGACCT
TGCCTGGCTCTACCCTGGACGGAGAGACAAGCAGCATTTTACCACCCTAATCAAGGAAATGAAGGCCGAA
TTTGCAAAGGAAGCCCAGCCAGGGAAAAAGCAGCTCCTGCTCAGTGCAGCAGTGTCTGCGGGGAAGGTCA
CCATTGACAGCAGCTATGACATTGCCCAGATATCCGAACACCTGGATTTCATTAGCATCATGACATACGA

TTTTCATGGAGCCTGGCGTGGGTCCACAGGCCATCATAGTCCCCTGTTCCGAGGCCAGGAGGATGCGAGT
CCTGACAGATTCAGCAACACTGACTATGCTGTGGGGTACATGTTGAGGTTGGGGGCTCCTGCCAGCAAGC
TGGTGATGGGCATCCCCACCTTCGGGAAAAGCTTCACTCTGGCCTCTTCTGAGACTGGTGTTGGAGCCCC
AATCTCGGGACCAGGAATTCCAGGCCGGTTCACCAAGGAGGCAGGGACCCTTGCCTACTATGAGATCTGT
GACTTCCTCCGCGGAGCCACAGTCCATAGAATCCTCGGCCAGCAGGTCCCCTATGCCACCAAAGGCAACC
AGTGGGTAGGATACGACGACCAGGAAAGCGTCAAAAGCAAGGTGCAGTACCTGAAGGACAGGCAGCTGGC
AGGCGCCATGGTATGGGCCCTGGACCTGGATGACTTCCAGGGCTCCTTCTGTGGCCAGGATCTGCGCTTC
CCTCTCACCAATGCCATCAAGGATGCACTCGCCGCAACT
>Macaca_mulatta
ATGTACAAACTGGTCTGCTACTACACCAGCTGGTCCCAGTACCGGGAAGGCGATGGGAGCTGCTTCCCAG
ATGCCATTGACCGCTTCCTCTGTACCCACATCATCTACAGCTTTGCCAATATAAGCAACGATCACATCGA
CACCTGGGAGTGGAATGATGTGACACTCTACGGCATGCTCAACACACTCAAGAACAGGAACCCCAATCTG
AAGACGCTCCTGTCTGTCGGAGGATGGAACTTTGGCTCTCAAAGATTTTCCAAGATGGCCTCCAACACCC
AGAGTCGCCAGACTTTCATCAAGTCAGTACCGCCATTTCTGCGCACCCACGGCTTTGATGGGTTGGACCT
TGCCTGGCTCTACCCTGGACGGAGAGACAAGCAGCATTTTACCACCCTAATCAAGGAAATGAAGGCCGAA
TTTGCAAAGGAAGCCCGGCCAGGGAAAAAGCAGCTCCTGCTCAGTGCAGCAGTGTCTGCGGGGAAGGTCA
CCATTGACAGCGGCTATGACATTGCCCAGATATCCGAACACCTGGATTTCATTAGCATCATGACATACGA
TTTTCATGGCGCCTGGCGTGGGACCACAGGCCATCATAGTCCCCTGTTCCGAGGCCAGGAGGATGCGAGT
CCTGACAGATTCAGCAACACTGACTATGCTGTGGGGTACATGTTGAGGTTGGGGGCTCCTGCCAGCAAGC
TGGTGATGGGCATCCCCACCTTCGGGAAAAGCTTCACTCTGGCCTCTTCTGAGACTGGTGTTGGAGCCCC
AATCTCGGGACCAGGAATTCCAGGCCGGTTCACCAAGGAGGCAGGGACCCTTGCCTACTATGAGATCTGT
GACTTCCTCCGCGGAGCCACAGTCCATAGAATCCTCGGCCAGCAGGTCCCCTATGCCACCAAAGGCAACC
AGTGGGTAGGATACGACGACCAGGAAAGCGTCAAAAGCAAGGTGCAGTACCTGAAGGACAGGCAGCTGGC
AGGCGCCATGGTATGGGCCCTGGACCTGGATGACTTCCAGGGCTCCTTCTGTGGCCAGGATCTGCGCTTC
CCTCTTACCAATGCCATCAAGGATGCCCTCGCCGCAACT
>Theropithecus_gelada
ATGTACAAACTGGTCTGCTACTACACCAGCTGGTCCCAGTACCGGGAAGGCGATGGGAGCTGCTTCCCAG
ATGCCATTGACCGCTTCCTCTGTACCCACGTCATCTACAGCTTTGCCAATATAAGCAACGATCACATCGA
CACCTGGGAGTGGAATGATGTGACACTCTACGGCATGCTCAACACACTCAAGAACAGGAACCCCAATCTG
AAGACGCTCCTGTCTGTCGGAGGATGGAACTTTGGCTCTCAAAGATTTTCCAAGATGGCCTCCAACACCC
AGAGTCGCCAGACTTTCATCAAGTCAGTACCGCCATTTCTGCGCACCCACGGCTTTGATGGGTTGGACCT
TGCCTGGCTCTACCCTGGACGGAGAGACAAGCAGCATTTTACCACCCTAATCAAGGAAATGAAGGCCGAA
TTTGCAAAGGAAGCCCAGCCAGGGAAAAAGCAGCTCCTGCTCAGTGCAGCAGTGTCTGCGGGGAAGGTCA
CCATTGACAGCAGCTATGACATTGCCCAGATATCCGAACACCTGGATTTCATTAGCATTATGACATACGA
TTTTCATGGAGCCTGGCGTGGGACCACAGGCCATCATAGTCCCCTGTTCCGAGGCCAGGAGGATGCGAGT
CCTGACAGATTCAGCAACACTGACTATGCTGTGGGGTACATGTTGAGGTTGGGGGCTCCTGCCAGCAAGC
TGGTGATGGGCATCCCCACCTTCGGGAAAAGCTTCACTCTGGCCTCTTCTGAGACTGGTGTTGGAGCCCC
AATCTCGGGACCAGGAATTCCAGGCCGGTTCACCAAGGAGGCAGGGACCCTTGCCTACTATGAGATCTGT
GACTTCCTCCGCGGAGCCACAGTCCATAGAATCCTCGGCCAGCAGGTCCCCTATGCCACCAAAGGCAACC
AGTGGGTAGGATACGACGACCAGGAAAGCGTCAAAAGCAAGGTGCAGTACCTGAAGGACAGGCAGCTGGC
AGGTGCCATGGTATGGGCCCTGGACCTGGATGACTTCCAGGGCTCCTTCTGTGGCCAGGATCTGCGCTTC
CCTCTCACCAATGCCATCAAGGATGCACTCGCCGCAACT
>Macaca_fascicularis
ATGTACAAACTGGTCTGCTACTACACCAGCTGGTCCCAGTACCGGGAAGGCGATGGGAGCTGCTTCCCAG
ATGCCATTGACCGCTTCCTCTGTACCCACATCATCTACAGCTTTGCCAATATAAGCAACGATCACATCGA
CACCTGGGAGTGGAATGATGTGACACTCTACGGCATGCTCAACACACTCAAGAACAGGAACCCCAATCTG
AAGACGCTCCTGTCTGTCGGAGGATGGAACTTTGGCTCTCAAAGATTTTCCAAGATGGCCTCGAACACCC
AGAGTCGCCAGACTTTCATCAAGTCAGTACCGCCATTTCTGCGCACCCACGGCTTTGATGGGTTGGACCT
TGCCTGGCTCTACCCTGGACGGAGAGACAAGCAGCATTTTACCACCCTAATCAAGGAAATGAAGGCCGAA
TTTGCAAAGGAAGCCGGGCCAGGGAAAAAGCAGCTCCTGCTCAGTGCAGCAGTGTCTGCGGGGAAGGTCA
CCATTGACAGCGGCTATGACATTGCCCAGATATCCGAACACCTGGATTTCATTAGCATCATGACATACGA
TTTTCATGGAGCCTGGCGTGGGACCACAGGCCATCATAGTCCCCTGTTCCGAGGCCAGGAGGATGCGAGT
CCTGACAGATTCAGCAACACTGACTATGCTGTGGGGTACATGTTGAGGTTGGGGGCTCCTGCCAGCAAGC
TGGTGATGGGCATCCCCACCTTCGGGAAAAGCTTCACTCTGGCCTCTTCTGAGACTGGTGTTGGAGCCCC
AATCTCGGGACCAGGAATTCCAGGCCGGTTCACCAAGGAGGCAGGGACCCTTGCCTACTATGAGATCTGT
GACTTCCTCCGCGGAGCCACAGTCCATAGAATCCTCGGCCAGCAGGTCCCCTATGCCACCAAAGGCAACC
AGTGGGTAGGATACGACGACCAGGAAAGCGTCAAAAGCAAGGTGCAGTACCTGAAGGACAGGCAGCTGGC
AGGCGCCATGGTATGGGCCCTGGACCTGGATGACTTCCAGGGCTCCTTCTGTGGCCAGGATCTGCGCTTC
CCTCTTACCAATGCCATCAAGGATGCCCTCGCCGCAACT
>Chlorocebus_sabaeus
ATGTACAAACTGGTCTGCTACTACACCAGCTGGTCCCAGTACCGGGAAGGCGATGGGAGCTGCTTCCCAG
ATGCCATTGACCGCTTCCTCTGTACCCACATCATCTACAGCTTTGCCAATATAAGCAACGATCACATCGA
CACCTGGGAGTGGAATGATGTGACGCTCTACGGCATGCTCAACACACTCAAGAACAGGAACCCCAATCTG
AAGACGCTCCTGTCTGTCGGAGGATGGAACTTTGGCTCTCAAAGATTTTCCAAGATGGCCTCCAACACCC
AGAGTCGCCAGACTTTCATCAAGTCAGTACCGCCATTTCTGCGCACCCACGGCTTTGATGGGTTGGACCT

TGCCTGGCTCTACCCTGGACGGAGAGACAAGCAGCATTTTACCACCCTAATCAAGGAAATGAAGGCCGAA
TTTGCAAAGGAAGCCCAGCCAGGGAAAAAGCAGCTCCTGCTCAGTGCAGCAGTGTCTGCGGGGAAGGTCA
CCATTGACAGCAGCTATGACATTGCCCAGATATCCGAACACCTGGATTTCATTAGCATCATGACGTACGA
TTTTCATGGAGCCTGGCGTGGGACCACAGGCCATCATAGTCCCCTGTTCCGAGGCCAGGAGGATGCGAGT
CCTGACAGATTCAGCAACACTGACTATGCTGTGGGGTACATGTTGAGGTTGGGGGCTCCTGCCAGCAAGC
TGGTGATGGGCATCCCCACCTTCGGGAAAAGCTTCACTCTGGCCTCTTCTGAGACTGGTGTTGGAGCCCC
AATCTCGGGACCAGGAATTCCAGGCCGGTTCACCAAGGAGGCAGGGACCCTTGCCTACTATGAGATCTGT
GACTTCCTCCACGGAGCCACAGTCCATAGAATCCTCGGCCAGCAGGTCCCCTATGCCACCAAGGGCAACC
AGTGGGTAGGATACGACGACCAGGAAAGCGTCAAAAGCAAGGTGCAGTACCTGAAGGACAGGCAGCTGGC
AGGCGCCATGGTATGGGCCCTGGACCTGGACGACTTCCAGGGCTCCTTCTGTGGCCAGGATCTGCGCTTC
CCTCTCACCAATGCCATCAAGGATGCACTCGCCGCAACT
>Cercocebus_atys
ATGTACAAACTGGTCTGCTACTACACCAGCTGGTCCCAGTACCGGGAAGGCGATGGGAGCTGCTTCCCAG
ATGCCATTGACCGCTTCCTCTGTACCCACATCATCTACAGCTTTGCCAATATAAGCAACGATCACATCGA
CACCTGGGAGTGGAATGATGTAACACTCTACGGCATGCTCAACACACTCAAGAACAGGAACTCCAATCTG
AAGACGCTCCTGTCTGTCGGAGGATGGAACTTTGGCTCTCAAAGATTTTCCAAGATGGCCTCCAACACCC
AGAGTCGCCAGACTTTCATCAAGTCAGTACCGCCATTTCTGCGCACCCACGGCTTTGATGGGTTGGACCT
TGCCTGGCTCTACCCTGGACGGAGAGATAAGCAGCATTTTACCACCCTAATCAAGGAAATGAAGGCCGAA
TTTGCAAAGGAAGCCCAGCCAGGGAAAAAGCAGCTCCTGCTCAGTGCAGCAGTGTCTGCGGGGAAGGTCA
CCATTGACAGCAGCTATGACATTGCCCAGATATCCGAACACCTGGATTTCATTAGCATCATGACATACGA
TTTTCATGGAGCCTGGCGTGGGACCACAGGCCATCATAGTCCCCTGTTCCGAGGCCAGGAGGATGCGAGT
CCTGACAGATTCAGCAACACTGACTATGCTGTGGGGTACATGTTGAGGTTGGGGGCTCCTGCCAGCAAGC
TGGTGATGGGCATCCCCACCTTCGGGAAAAGCTTCACTCTGGCCTCTTCTGAGACTGGTGTTGGAGCCCC
AATCTCGGGACCAGGAATTCCAGGCCGGTTCACCAAGGAGGCAGGGACCCTTGCCTACTATGAGATCTGT
GACTTCCTCCGCGGAGCCACAGTCCATAGAATCCTCGGCCAGCAGGTCCCCTATGCCACCAAAGGCAACC
AGTGGGTAGGATACGACGACCAGGAAAGCGTCAAAAGCAAGGTGCAGTACCTGAAGGACAGGCAGCTGGC
AGGCGCCATGGTATGGGCCCTGGACCTGGATGACTTCCAGGGCTCCTTCTGTGGCCAGGATCTGCGCTTC
CCTCTCACCAATGCCATCAAGGATGCACTTGCCGCAACT
>Rhinopithecus_roxellana
ATGTACAAACTGGTCTGCTACTACACCAGCTGGTCCCAGTACCGGGAAGGCGACGGGAGCTGCTTCCCAG
ATGCCATCGACCGTTCCCTCTGTACCCACATCATCTACAGCTTTGCCAATATAAGCAACGATCACATTGA
CACCTGGGAGTGGAATGATGTGACGCTTTACGGCATGCTCAACACACTCAAGAACAGGAACCCCAATCTG
AAGACGCTCCTGTCTGTCGGAGGATGGAACTTTGGCTCTCAAAGATTTTCCAAGATGGCCTCCAACACCC
AGAGTCGCCAGACTTTCATCAAGTCAGTACCGCCATTTCTGCGCACCCACGGCTTTGATGGGCTGGACCT
TGCCTGGCTCTACCCTGGACGGAGAGACAAGCAGCATTTTACCACCCTAATCAAGGAAATGAAGGCTGAA
TTTGCAAAGGAAGCCCAGCCAGGGAAAAAGCAGCTCCTGCTCAGTGCAGCAGTGTCTGCGGGGAAGGTCA
CCATTGACAGCAGCTATGACATTGCCCAGATATCGAACACCTGGATTTCATTAGCATCATGACGTACGA
TTTTCATGGAGCCTGGCGTGGGACCACAGGCCATCATAGTCCCCTGTTCCGAGGCCAGGAGGATGCGAGT
CCTGACAGATTTAGCAACACTGACTATGCTGTGGGGTACATGTTGAGGTTGGGGGCTCCTGCCGGCAAGC
TGGTGATGGGCATCCCCACCTTCGGGAAAAGCTTCACTCTGGCCTCTTCTGAGACTGGTGTTGGAGCCCC
AATCTCGGGACCAGGAATTCCAGGCCGGTTCACCAAGGAGGCAGGGACTCTTGCCTACTATGAGATCTGT
GACTTCCTCCGCGGAGCCACAGTCCATAGAATCCTCGGCCAGCAGGTCCCCTATGCCACCAAAGGCAACC
AGTGGGTAGGATACGACGACCAGGAAAGCGTCAAAAGCAAGGTGCAGTACCTGAAGGACAGGCAGCTGGC
AGGTGCCATGGTATGGGCCCTGGACCTGGATGACTTCCAGGGCTCCTTCTGCGGCCAGGATCTGCGCTTC
CCTCTCACCAATGCCATCAAGGATGCACTCGCCGCAACT
>Colobus_angolensis
ATGTACAAACTGGTCTGCTACTACACCAGCTGGTCCCAGTACCGGGAAGGCGACGGGAGCTGCTTCCCAG
ATGCCATTGACCGCTTCCTCTGTACCCACATCATCTACAGCTTTGCCAATATAAGCAACGATCACATCGA
CACCTGGGAGTGGAATGATGTGACGCTCTATGGCACGCTCAACACACTCAAGAACAGGAACCCCAATCTG
AAGACGCTCCTGTCTGTCGGAGGATGGAACTTTGGCTCTCAAAGATTTTCCAAGATGGCCTCCAACACCC
AGAGTCGCCAGACTTTCATCAAGTCAGTACCGCCATTTCTGCGCACCCACGGCTTTGATGGGCTGGACCT
TGCCTGGCTCTACCCTGGACGGAGAGACAAGCAGCATTTTACCACCCTAATCAAGGAAATGAAGGCCGAA
TTTGCAAAGGAAGCCCAGCCAGGGAAAAAGCAGCTCCTGCTCAGTGCAGCAGTGTCTGCGGGGAAGGTCA
CCATTGACAGCAGCTATGACATTGCCCAGATATCCGAACACCTGGATTTCATTAGCATCATGACGTACGA
TTTTCATGGAGCCTGGCGTGGGACCACAGGCCATCATAGTCCCCTGTTCCGAGGCCAGGAGGATGCGAGT
CCTGACAGATTTAGCAACACTGACTATGCTGTGGGGTACATGTTGAGGTTGGGGGCTCCTGCCGGCAAGC
TGGTGATGGGCATCCCCACCTTCGGGAAAAGCTTCACCCTGGCCTCTTCTGAGACTGGTGTTGGAGCCCC
AATCTCCGGACCAGGAATTCCAGGCCGGTTCACCAAGGAGGCAGGGACCCTTGCCTACTATGAGATCTGT
GACTTCTTCCGCGGAGCCACAGTCCATAGAATCCTCGGCCAGCAGGTCCCCTATGCCACCAAAGGCAACC
AGTGGGTAGGATACGACGACCAGGAAAGCGTCAAAAGCAAGGTGCAGTACCTGAAGGACAGGCAGCTGGC
AGGCGCCATGGTATGGGCCCTGGACCTGGATGACTTCCAGGGCTCCTTCTGCGGCCAGGATCTGCGCTTC
CCTCTCACCAATGCCATCAAGGATGCACTCGCCGCAACT
>Carlito_syrichta
ATGTACAAACTGGTCTGCTACTATACCAGCTGGTCCCAGTACCGAGAGGGCGATGGAAGCTGCTTCCCTG
ATGCCATTGACCACTCCCTCTGCACTCACATCATCTACAGCTTTGCCAACATAAGCAACAACCAGATTGA

CACCTGGGAGTGGAATGATGTGACGCTCTATGACACGCTGAACACACTCAAGAACAGGAATCCCAACCTG
AAGACCCTCCTGTCTGTCGGAGGATGGAACTTTGGATCTCAGAGATTTTCCAAAATAGCCTCCAATGCCC
AGAGTCGCAGGACTTTCATCGAGTCAGTGCCACCATTTCTACGGACCCATGGCTTTGATGGGCTGGACCT
AGCCTGGCTCTACCCAGAACGGAGAGACAAGCAGCATTTCACCACTCTAATCAAGGAAATGAAGACTGAA
TTTGCGAAGGAAGCCCAGTCAGGAAAAGAGCAGCTCCTGCTCAGTGCCGCAGTGTCTGCGGGGAAGGTCA
CCATTGACAGGGGCTATGACGTCGCCCAGATATCCCAACACCTGGATTTTATGAGCATCATGACCTACGA
TTTTCACGGAGTCTGGCGCCAGACTACAGGTCATCACAGTCCCCTGTTTCGCGGCCAGTCGGATACAAGT
CCTGACAGATTCAGCAATGTTGACTATGCTGTGGGGTACATGTTGAGACTGGGGGCTCCTGCCAACAAGC
TGGTGATGGGCATCCCCACCTTCGGGAAGAGCTTCACTCTGGCCTCTTCTGAGACGGGTGTCGGAGCCCC
AATCTTGGGGCCAGGAACACCAGGCCGGTTCACCAAGGAGGCGGGGACTCTCGCCTACTATGAGATCTGT
GACTTCCTCCACGAGGCCACAGTCCATAGACTCCTGGGCCAGCAGGTCCCCTATGCCACCAAGGGCAACC
AGTGGGTGGGATATGATGACCAGGAGAGCATCAAACGCAAGGTGCAGTACCTGAAGAACAGGCAGCTGGC
GGGCGCCATGGTGTGGACCCTGGACCTGGATGACTTCCAGGGCTCCTTCTGCAGCCAGAATCTGCGCTTC
CCTCTCACCAGTGCCATCAAGGATGCACTCACTTCGACT
>Callithrix_jacchus
ATGTACAAACTGGTCTGCTACTACACCAGCTGGTCCCAGTACCGGGAAGGCGATGGGAGCTGCTTCCCAG
ATGCCATTGACCGCTCCCTCTGTACCCACATCATCTACAGCTTTGCCAATATAAGCAACGATCACATCGA
CACCTGGGAGTGGAATGATGTGACACTCTACGACACGCTCAACACACTCAAGAACAGGAACCCCAACCTG
AAGACCCTCCTGTCAGTTGGAGGATGGAACTTTGGCTCTCAAAGATTTTCCAAGATCGCCTCCAACACCC
AGAGTCGCCGGACTTTCATCAAGTCGGTGCCGCCATTTCTACGCACCCATGGCTTTGATGGGCTGGACCT
CGCCTGGCTCTACCCTGGACGGAGAGACAAGCAGCATTTTACTACCCTAATCAAGGAAATGAAGGCCGAA
TTTGCAAAGGAAGCGCAGCAAGGGAAAGAGCAGCTCCTGCTCAGCGCAGCAGTGTCTGCGGGGAAGGTCA
CCATTGACAGCAGCTATGACATTGCCCGGATATCCCAACACCTGGATTTCATCAGCATCATGACCTATGA
CTTTCACGGAGCCTGGCGTGGGACCACAGGCCATCACAGTCCCCTGTTCCGAGGCCAGGAGGATGCAAGT
CCTGACAGATTCAGCAACGCTGACTATGCTGTGGGGTATATGCTGAGGCTGGGGGCTCCTGCCAGTAAGC
TGCTGATGGGCATCCCCACCTTCGGGAAGAGCTTCACTCTGGCCTCTTCTGAAACTGGTGTTGGAGCCCC
AGTCTCAGGACCAGGAATTCCAGGCCGGTTCACCAAGGAGGCGGGGACCCTTGCCTACTATGAGATCTGT
GACTTCCTCCACGGAGCCACAGTCCATAGAATCCTCGGCCAGCAGGTCCCCTATGCCACCAAGGGCAACC
AGTGGGTAGGATATGATGACCAGGAAAGCGTCAAAAGCAAGGTGCAGTACCTGAAGGACAGGCAGCTGGC
AGGTGCCATGGTATGGGCCCTGGACCTGGATGATTTCCAGGGCTCCTTCTGCGGCCGGGATCTGCGCTTC
CCTCTCATCAATGCCGTCAAGGACGCACTCGCTGCAACT
>Trachypithecus_francoisi
ATGTACAAACTGGTCTGCTACTACACCAGCTGGTCCCAGTACCGGGAAGGCGACGGGAGCTGCTTCCCAG
ATGCCATCGACCGCTCCCTCTGTACCCACATCATCTACAGCTTTGCCAATATAAGCAACGATCACATCGA
CACCTGGGAGTGGAATGATGTGACGCTTTACGGCATGCTCAACACACTCAAGAACAGGAACCCCAATCTG
AAGACGCTCCTGTCTGTCGGAGGATGGAACTTTGGCTCTCAAAGATTTTCCAAGATGGCCTCCAACACCC
AGAGTCTCCAGACTTTCATCAAGTCAGTACCGCCATTTCTGCGCACCCACGGCTTTGATGGGCTGGACCT
TGCCTGGCTCTACCCTGGACGGAGAGACAAGCAGCATTTTACCACCCTAATCAAGGAAATGAAGGCTGAA
TTTGCAAAGGAAGCCCAGCCAGGGAAAAAGCAGCTCCTGCTCAGTGCAGCAGTGTCTGCGGGGAAGGTCA
CCATTGACAGCAGCTATGACATTGCCCAGATATCCGAACACCTGGATTTCATTAGCATCATGACGTACGA
TTTTCATGGAGCCTGGCGTGGGACCACAGGCCATCATAGTCCCCTGTTCCGAGGCCAGGAGGATGCGAGT
CCTGACAGATTTAGTAACACTGACTATGCTGTGGGGTACATGTTGAGGTTGGGGGCTCCTGCCGGCAAGC
TGGTGATGGGCATCCCCACCTTCGGGAAAAGCTTCACTCTGGCCTCTTCTGAGACTGGTGTTGGAGCCCC
AATCTCGGGACCAGGAATTCCAGGCCGGTTCACCAAGGAGGCAGGGACTCTTGCCTACTATGAGATCTGT
GACTTCCTCCGCGGAGCCACAGTCCATAGAATCCTCGGCCAGCAGGTCCCCTATGCCACCAAAGGCAACC
AGTGGGTAGGATACGACGACCAGGAAAGCGTCAAAAGCAAGGTGCAGTACCTGAAGGACAGGCAGCTGGC
AGGCGCCATGGTATGGGCCCTGGACCTGGATGACTTCCAGGGCTCCTTCTGCGGCCAGGATCTGCGCTTC
CCTCTCACCAATGCCATCAAGGATGCACTGGCTGCAACT
>Mandrillus_leucophaeus
ATGTACAAACTGGTCTGCTACTACACCAGCTGGTCCCAGTACCGGGAAGGCGATGGGAGCTGCTTCCCAG
ATGCCATTGACCGCTTCCTGTGTACCCACATCATCTACAGCTTTGCCAATATAAGCAACGATCACATCGA
CACCTGGGAGTGGAATGATGTGACACTCTACGGCATGCTCAACACACTCAAGAACAGGAACCCCAATCTG
AAGACGCTCCTGTCTGTCGGAGGATGGAACTTTGGCTCTCAAAGATTTTCCAAGATGGCCTCCAACGCCC
AGAGTCGCCAGACTTTCATCAAGTCAGTACCGCCATTTCTGCGCACCCATGGCTTTGATGGGTTGGACCT
TGCCTGGCTCTACCCTGGACGGAGAGACAAGCAGCATTTTACCACCCTAATCAAGGAAATGAAGGCTGAA
TTTGCAAAGGAAGCCCAGCCAGGGAAAAAGCAGCTCCTGCTCAGTGCAGCAGTGTCTGCGGGGAAGGTCA
CCATTGACAGCAGCTATGACATTGCCCAGATATCCGAACACCTGGATTTCATTAGCATCATGACATACGA
TTTTCATGGAGCCTGGCGTGGGACCACAGGCCATCATAGTCCCCTGTTCCGAGGCCAGGAGGATGCGAGT
CCTGACAGATTCAGCAACACTGACTATGCTGTGGGGTACATGTTGAGGTTGGGGGCTCCTGCCAGCAAGC
TGGTGATGGGCATCCCCACCTTTGGGAAAAGCTTCACTCTGGCCTCTTCTGAGACTGGTGTTGGAGCCCC
AATCTCGGGACCAGGAATTCCAGGCCGGTTCACCAAGGAGGCAGGGACCCTTGCCTACTATGAGATCTGT
GACTTCCTCCGCGGAGCCACAGTCCATAGAATCCTCGGCCAGCAGGTCCCCTATGCCACCAAAGGCAACC
AGTGGGTAGGATACGACGACCAGGAAAGCGTCAAAAGCAAGGTGCAGTACCTGAAGGACAGGCAGCTGGC
AGGCGCCATGGTATGGGCCCTGGACCTGGATGACTTCCAGGGCTCCTTCTGTGGCCAGGATCTGCGCTTC
CCTCTCACCAATGCCATCAAGGATGCACTTGCCGCAACT

>Pan_paniscus
ATGTACAAACTGGTCTGCTACTACACCAGCTGGTCCCAGTACCGGGAAGGCGATGGGAGCTGCTTCCCAG
ATGCCATTGACCGCTTCCTCTGTACCCACATCATCTACAGCTTTGCCAATATAAGCAACGATCACATCGA
CACCTGGGAGTGGAATGATGTGACGCTCTACGGCATGCTCAACACACTCAAGAACAGGAACCCCAACCTG
AAGACTCTCTTGTCTGTCGGAGGATGGAACTTTGGGTCTCAAAGATTTTCCAAGATAGCCTCCAACACCC
AGAGTCGCCGGACTTTCATCAAGTCAGTACCGCCATTTCTGCGCACCCATGGCTTTGATGGACTGGACCT
TGCCTGGCTCTACCCTGGACGGAGAGACAAACAGCATTTTACCACCCTAATCAAGGAAATGAAGGCCGAA
TTTATAAAGGAAGCCCAGCCAGGGAAAAAGCAGCTCCTGCTCAGTGCAGCACTGTCTGCGGGGAAGGTCA
CCATTGACAGCAGCTATGACATTGCCAAGATATCCCAACACCTGGATTTCATTAGCATCATGACCTACGA
TTTTCATGGAGCCTGGCGTGGGACCACAGGCCATCACAGTCCCCTGTTCCGAGGTCAGGAGGATGCAAGT
CCTGACAGATTCAGCAACACTGACTATGCTGTGGGGTACATGTTGAGGCTGGGGGCTCCTGCCAGTAAGC
TGGTGATGGGCATCCCCACCTTCGGGAGGAGCTTCACTCTGGCTTCTTCTGAGACTGGTGTTGGAGCCCC
AATCTCAGGACCGGGAATTCCAGGCCGGTTCACCAAGGAGGCAGGGACCCTTGCCTACTATGAGATCTGT
GACTTCCTCCGCGGAGCCACAGTCCATAGAATCCTCGGTCAGCAGGTCCCCTATGCCACCAAGGGCAACC
AGTGGGTAGGATACGACGACCAGGAAAGCGTCAAAAGCAAGGTGCAGTACCTGAAGGACAGGCAGCTGGC
GGGCGCCATGGTATGGGCCCTGGACCTGGATGACTTCCAGGGCTCCTTCTGCGGCCAGGATCTGCGCTTC
CCTCTCACCAATGCCATCAAGGATGCACTCGCTGCAACG
>Nomascus_leucogenys
ATGTACAAACTGGTCTGCTACTACACCAGCTGGTCCCAGTACCGGGAAGGCGATGGGAGCTGCTTCCCAG
ATGCCATTGACCGCTTCCTCTGTACCCACATCATCTACAGCTTTGCCAATATAAGCAACGATCACATCGA
CACCTGGGAGTGGAATGATGTGACACTCTACGGCATGCTCAACACACTCAACAACAGGAACCCCAACCTG
AAGACTCTCCTGTCTGTCGGAGGATGGAACTTTGGCTCTCAAAGATTTTCCAAGATAGCCTCCAACACCC
AGAGTCGCCGCACTTTCATCAAATCAGTACCGCCATTTCTGCGCACCCATGGCTTTGATGGGCTGGACCT
TGCCTGGCTTTACCCTGGACGGAGAGACAAGCAACATTTTACCACCCTGATCAAGGAAATGAAGGCCGAA
TTTGCAAAGGAAGCCCAGCCAGGGAAAAAGCAGCTCCTGCTCAGCGCAGCAGTGTCTGCGGGGAAGGTCA
CCATTGACAGCAGCTATGACATTGCCAAGATATCCCAACACCTGGATTTCATTAGCATCATGACCTACGA
TTTTCATGGTGCCTGGCGTGGGACCACAGGCCATCACAGTCCCCTGTTCCGAGGCCAGGAGGATGCAAGT
CCTGACAGATTCAGCAACACTGACTATGCTGTGGGGTACATGTTGAGGCTGGGGGCTCCTGCCAGTAAGC
TGGTGATGGGCATCCCCACCTTCGGGAAGAGCTTCACTCTGGCTTCTTCTGAGGCTGGTGTTGGAGCCCC
AATCTCAGGACCAGGAATTCCAGGCCGGTTCACCAAGGAGGCAGGGACCCTTGCCTACTATGAGATCTGT
GACTTCCTGCGCGGAGCCACAGTCCATAGAATCCTCGGCCAGCAGGTCCCCTATGCCACCAAGGGCAACC
AGTGGGTAGGATACGACGACCAGGAAAGCATCAAAAGCAAGGTGCAGTACCTGAAGGACAGGCAGCTGGC
GGGCGCCATGGTATGGGCCCTGGATCTGGATGACTTCCAGGGCTCCTTCTGCGGCCAGGATCTGCGCTTC
CCTCTCATCAATGCCATCAAGGATGCACTCGCTGCAACT
>Gorilla_gorilla
ATGTACAAACTGGTCTGCTACTACACCAGCTGGTCCCAGTACCGGGAAGGCGATGGGAGCTGCTTCCCAG
ATGCCATTGACCGCTTCCTCTGTACCCACATCATCTACAGCTTTGCCAATATAAGCAACGATCACATCGA
CACCTGGGAGTGGAATGATGTGACGCTCTACGGCATGCTCAACACACTCAAGAACAGGAACCCCAACCTG
AAGACTCTCTTGTCTGTCGGAGGATGGAACTTTGGGTCTCAAAGATTTTCCAAGATAGCCTCCAACACCC
AGAGTCGCCGGACTTTCGTCAAGTCAGTACCGCCATTTCTGCGTACCCATGGCTTTGATGGGCTGGACCT
TGCCTGGCTCTACCCTGGACGGAGAGACAAACAGCATTTTACCACCCTAATCAAGGAAATGAAGGCCGAA
TTTATAAAGGAAGCCCAGCCAGGGAAAAAGCAGCTCCTGCTCAGCGCAGCACTGTCTGCGGGGAAGGTCA
CCATTGACAGCAGCTATGACATTGCCAAGATATCCCAACACCTGGATTTCATTAGCATCATGACCTACGA
TTTTCATGGAGCCTGGCGTGGGACCACAGGCCATCACAGTCCCCTGTTCTGAGGTCAGGAGGATGCAAGT
CCTGACAGATTCAGCAACACTGACTATGCTGTGGGGTACATGTTGAGGCTGGGGGCTCCTGCCAGTAAGC
TGGTGATGGGCATCCCCACCTTCGGGAGGAGCTTCACTCTGGCTTCTTCTGAGACTGGTGTTGGAGCCCC
AAGCTCAGGACCGGGAATTCCAGGCCGGTTCACCAAGGAGGCAGGGACCCTTGCCTACTATGAGATCTGT
GACTTCCTCGGCGGAGCCACAGTCCATAGAATCCTCGGCCAGCAGGTCCCCTATGCCACCAAGGGCAACC
AGTGGGTAGGATACGACGACCAGGAAAGCGTCAAAAACAAGGTGCAGTACCTGAAGGACAGGCAGCTGGC
GGGCGCCATGGTATGGGCCCTGGACCTGGATGACTTCCAGGGCTCCTTCTGCGGCCAGGATCTGCGCTTC
CCTCTCACCAATGCCATCAAGGATGCACTCGCTGCAACA
>Pan_troglodytes
ATGTACAAACTGGTCTGCTACTACACCAGCTGGTCCCAGTACCGGGAAGGCGATGGGAGCTGCTTCCCAG
ATGCCATTGACCGCTTCCTCTGTACCCACATCATCTACAGCTTTGCCAATATAAGCAACGATCACATCGA
CACCTGGGAGTGGAATGATGTGACGCTCTACGGCATGCTCAACACACTCAAGAACAGGAACCCCAACCTG
AAGACTCTCTTGTCTGTCGGAGGATGGAACTTTGGGTCTCAAAGATTTTCCAAGATAGCCTCCAACACCC
AGAGTCGCCGGACTTTCATCAAGTCAGTACCGCCATTTCTGCGCACCCATGGCTTTGATGGACTGGACCT
TGCCTGGCTCTACCCTGGACGGAGAGACAAACAGCATTTTACCACCCTAATCAAGGAAATGAAGGCCGAA
TTTATAAAGGAAGCCCAGCCAGGGAAAAAGCAGCTCCTGCTCAGTGCAGCACTGTCTGCGGGGAAGGTCA
CCATTGACAGCAGCTATGACATTGCCAAGATATCCCAACACCTGGATTTCATTAGCATCATGACCTACGA
TTTTCATGGAGCCTGGCGTGGGACCACAGGCCATCACAGTCCCCTGTTCCGAGGTCAGGAGGATGCAAGT
CCTGACAGATTCAGCAACACTGACTATGCTGTGGGGTACATGTTGAGGCTGGGGGCTCCTGCCAGTAAGC
TGGTGATGGGCATCCCCACCTTCGGGAGGAGCTTCACTCTGGCTTCTTCTGAGACTGGTGTTGGAGCCCC
AATCTCAGGACCGGGAATTCCAGGCCGGTTCACCAAGGAGGCAGGGACCCTTGCCTACTATGAGATCTGT
GACTTCCTCCGCGGAGCCACAGTCCATAGAATCCTCGGCCAGCAGGTCCCCTATGCCACCAAGGGCAACC

AGTGGGTAGGATACGACGACCAGGAAAGCGTCAAAAGCAAGGTGCAGTACCTGAAGGACAGGCAGCTGGC
GGGCGCCATGGTATGGGCCCTGGACCTGGATGACTTCCAGGGCTCCTTCTGCGGCCAGGATCTGCGCTTC
CCTCTCACCAATGCCATCAAGGATGCACTCGCTGCAACG
>Saimiri_boliviensis
ATGTACAAACTGGTCTGCTACTACACCAGCTGGTCCCAGTACCGGGAAGGCGATGGGAGCTGCTTCCCAG
ATGCCATTGACCGCTCCCTCTGTACTCACGTCATCTACAGCTTTGCCAACATAAGCAACGATCACATCGA
CACCTGGGAGTGGAACGATGTCACGCTCTACGACATGCTCAACACTCTCAAGAACAGGAACCCCAACCTG
AAGACCCTCCTGTCAGTTGGAGGATGGAACTTTGGCTCTCAAAGATTTTCCAAGATCGCCTCCAACACCC
AGAGTCGCCGGACTTTCATCAAGTCGGTGCCGCCATTTCTGCGCTCCCATGGATTTGATGGGCTGGACCT
TGCCTGGCTCTACCCTGGACGGAGAGACAAGCAGCATTTTACTACCCTAATCAAGGAAATGAAGGCCGAA
TTTGCAAAGGAAGCCCAGCAAGGGAAAGAGCAGCTCCTGCTTAGTGCAGCACTGTCTGCGGGGAAGGTCA
CCATTGACAGCAGCTATGACATTGCCAGGATATCCCAACACCTGGATTTCATCAGCATCATGACCTATGA
CTTTCACGGAGCCTGGCGTGGGACCACAGGCCATCACAGTCCCCTTTTCCGAGGCCAGGTGGATGCAAGT
CCTGACAGATTCAGCAACACTGACTATGCTGTGGGGTACATGCTGAGGCTGGGGGCTCCTGCCAGTAAGC
TGGTGATGGGCATCCCCACCTTCGGGAAGAGCTTCACTCTGGCCTCTTCTGAAACCGGTATTGGAGCCCC
AGTCTCGGGACCAGGACTTCCAGGCCATTTCACCAAGGAGGCAGGGACCCTTGCCTACTACGAGATCTGT
GACTTCCTCCGCGGAGCCACAGTCCATAGAATCCTCGGCCAGCAGGTGCCCTATGCCACCAAGGGCAACC
AGTGGGTAGGATATGACGACCAGGAAAGCGTCAAAAGCAAGGTGCAGTACCTGAAGGACAGGCAGCTGGC
GGGCGCCATGGTGTGGGCCCTGGACCTGGATGACTTCCAGGGCTCCTTCTGCGGCCAGGATCTGCGCTTC
CCTCTCATCAATGCCATCAAGGACGCCCTCGCTGCAACT
>Piliocolobus_tephrosceles
ATGTACAAACTGGTCTGCTACTACACCAGCTGGTCCCAGTACCGGGAAGGCGACGGGAGCTGCTTCCCAG
ATGCCATTGACCGCTTCCTCTGTACCCACATCATCTACAGCTTTGCCAATATAAGCAACGATCACATCGA
CACCTGGGAGTGGAATGATGTGACGCTCTACGGCATGCTCAACACACTCAAGAACAGGAACCCCAATCTG
AAGACGCTCTTGTCTGTCGGAGGATGGAACTTTGGCTCTCAAAGATTTTCCAAGATGGCCTCCAACACCC
AGAGTCGCCAGACTTTCATCAAGTCAGTACCGCCATTTCTGCGCACCCATGGCTTTGATGGGCTGGACCT
TGCCTGGCTCTACCCTGGATGGAGAGACAAGCAGCATTTTACCACCCTAATCAAGGAAATGAAGGCCGAA
TTTGCAAAGGAAGCCCAGCCAGGGAAAAAGCAGCTCCTGCTCAGTGCAGCAGTGTCTGCGGGGAAGGTCA
CCATTGACAGCAGCTATGACATTGCCCAGATATCTGAACACCTGGATTTCATTAGCATCATGACGTACGA
TTTTCATGGAGCCTGGCGTGGGACCACAGGCCATCATAGTCCCCTGTTCCGAGGCCAGGAAGATGCGAGT
CCTGACAGATATAGCAACACTGACTATGCTGTGGGGTACATGTTGAGGCTGGGGGCTCCTGCCGGCAAGC
TGGTGATGGGCATCCCCGCCTTCGGGAAAAGCTTCACTCTGGCCTCTTCTGAGACTGGTGTTGGAGCCCC
AATCTCCGGACCAGGAATTCCAGGCCGGTTCACCAAGGAGGCAGGGACCCTTGCCTACTATGAGATCTGT
GACTTCCTCCGCGGAGCCACAGTCCATAGAATCCTCGGCCAGCAGGTCCCCTATGCCACCAAAGGCAACC
AGTGGGTAGGATACGACGACCAGGAAAGCGTCAAAAGCAAGGTGCAGTACCTGAAGGACAGGCAGCTGGC
AGGCGCCATGGTATGGGCCCTGGACCTGGATGACTTCCAGGGCTCCTTCTGTGGCCAGGATCTGCGCTTC
CCTCTCACCAATGCCATCAAGGATGCACTCGCCACAACT
>Sapajus_apella
ATGTACAAGCTGGTCTGCTACTACACCAACTGGTCCCAGTACCGGGAAGGCGATGGGAGCTGCTTCCCAG
ATGTCATTGACCGCTCCCTCTGTACCCACATCATCTACAGCTTTGCCAATATAAGCAACGATCACATCGA
CACCTGGGAGTGGAATGATGTGACGCTGTACGACATGCTCAACGCACTCAAGAACAGGAACCCAAACCTG
AAGACCCTCCTGTCAGTTGGAGGATGGAACTTTGGCTCTCAAAGATTTTCCAAGATCGCCTCCAACACCC
AGAGTCGCCGGACTTTCATCAAGTCGGTGCCGCCATTTCTGCGCAGCCATGGTTTTGATGGGCTGGACCT
TGCCTGGCTCTACCCTGGACGGAGAGACAAGCAGCATTTTACTACCCTAATCAAGGAAATGAAGGCTGAA
TTTGCAAAGGAAGCCCAGCAAGGGAAAGAGCAGCTCCTGCTTAGCGCAGCAGTGTCTGCGGGGAAGGTCA
CCATTGACAGCAGCTATGACATTGCCCGGATATCCCAACACCTGGACTTCATCAGCATCATGACCTATGA
CTTTCACGGAGCCTGGCGTGGGACCACAGGCCATCACAGTCCCCTGTTCCGAGGCCAGGCGGATGCAAGT
CCTGACAGATTCAGCAACGCTGACTATGCTGTGGGGTACATGCTGAGGCTGGGAGCTCCTGCCAGTAAGC
TGGTGATGGGCATCCCCACCTTCGGGAAGAGCTTCACTCTGGCCTCTTCTGAAACTGGTGTTGGAGCCCC
AGTCTCGGGACCAGGAATTCCAGGCCGGTTCACCAAGGAGGCAGGGACCCTTGCCTACTATGAGATCTGT
GACTTCCTCCAGGGAGCCACAGTCCATAGAATCCTCGGCCAGCAGGTGCCCTATGCCACCAAGGGCAACC
AGTGGGTAGGATATGACGACCAGGAAAGCGTCAAAAGCAAGGTGCAGTACCTGAAGGACAGGCAGCTGGC
AGGTGCCATGGTATGGGCCCTGGACCTGGATGATTTCCAGGGCTCCTTCTGCGGCCAGGATCTGCGCTTC
CCTCTCATCAATGCCATCAAGGACGCACTCACTGCAACT
>Pongo_abelii
ATGTACAAACTGGTCTGCTACTACACCAGCTGGTCCCAGTACCGGGAAGGCGATGGGAGCTGCTTCCCAG
ATGCCATTGACCGCTTCTTGTGTACCCACATCATCTACAGCTTTGCCAATATAAGCAACGATCACATCGA
CACCTGGGAGTGGAATGATGTGACGCTCTACGGCATGCTCAACACACTCAAGAACAGGAACCCCAACCTG
AAGACTCTCCTGTCTGTGGGAGGATGGAACTTTGGGTCTCAAAGATTTTCCAACATAGCCTCCAACACCC
AGAGTCGTCGGACTTTCATCAAGTCAGTACCGCCATTTCTGCGCACCCATGGCTTTGATGGGCTGGACCT
TGCCTGGCTCTACCCTGGACAGAGAGATAAGCAGCATTTTACCACCCTAATCAAGGAAATGAGGGCCGAA
TTTATAAAGGAAGCCCAGCCAGGGAAAAAGCAGCTCCTGCTCAGCGCCGCAGTGTCTGCGGGGAAGGTCA
CCATTGACAGCAGCTATGACATTGCCAAGATATCCCAACACCTGGATTTCATTAGCATCATGACCTACGA
TTTTCATGGAGCCTGGCGAGGGACCACAGGCCATCACAGTCCCCTGTTCCGAGGCCAGGAGGATGCAAGT
CCTGACAGATTCAGCAACACTGACTATGCTGTGGGGTACATGTTGAGGCTGGAGGCTCCTGCCAGTAAGC

TGGTGATGGGCATCCCCACCTTCGGGAGGAGCTTCACTCTGGCTTCTTCTGAGACTGGTGTTGGAGCCCC
AATCTCAGGACCAGGAATTCCAGGCCGGTTCACCAAGGAGGCAGGGACCCTTGCCTACTATGAGATCTGT
GACTTCCTCCGCGGAGCCACAGTCCATAGAATCCTCGGCCAGCAGGTCCCCTATGCAACCAAGGGCAACC
AGTGGGTAGGATACGACGACCAGGAAAGCGTCAAAAGCAAGGTGCAGTACCTGAAGGAAAGGCAGCTGGC
GGGCGCCATGGTATGGGCCCTGGACCTGGATGACTTCCAGGGTTCCTTCTGCGGCCAGGATCTGCGATTC
CCTCTCACCAATGCCATCAAGGATGCACTCGCTGCAACT
>Galeopterus_variegatus
ATGTACAAACTGGTCTGCTACTACACCAGCTGGTCCCAGTACCGGGAAGGTGATGGGAGCTGCTTTCCAG
ATGCCATCGACCCTTTCCTCTGCACCCACATCGTTTACAGCTTTGCCAACATAAGCAACAATGAGATCGA
CACCTGGGAGTGGAATGACGTGACACTCTATGACACACTGAATGCACTCAAGAACAGGAACCCCAACCTG
AAGACCCTCCTGTCTGTTGGAGGATGGAGTTTTGGCTCTCAAAGATTTTCCAAGATAGCCTCCAACACCC
AGAGTCGCATGACTTTTATCAAGTCAGTGCCACCGTTTCTGCGGACCCATGGCTTTGATGGTCTGGACCT
AGCCTGGCTCTACCCTGGACGGAGAGACAAGCGGCATTTCACCACCCTGATCAAGGAAATGAAGGCTGAA
TTTGCAAAGGAAGCCAAGGCAGGAACAAAGCAGCTTCTGCTCAGTGCAGCGCTGTCTGCAGGGAAGGTCC
AGATTGACAGAAGCTATGACATCGCCCAGATATCCCAACACCTGGATTTCATTAGCATCATGGCCTATGA
TTTTCATGGAAACTGGCACCAGGCCACAGCACATCACAGCCCCCTGTTCCGAGGCCAGGTGGATGCAAGT
CCTGACAGATTCAGCAATGCTGACTATGCTGTGGGGTACATGCTGAGGCTGGGGGCCCCGGCCAATAAGC
TGGTGATGGGCATCCCCACCTTCGGGAAGAGCTTCACTCTGGCCTCTTCTAAAACGGGTGTCGGAGCTCC
AGTCTTGGGGCAGGGAATACCAGGCCAGTTCACCAAGGAGAAAGGGATCCTCGCCTACTATGAGATCTGT
GACTTCCTCCACGGAGCCACGGTCCACAGACTCCTCGGGCAGCAGGTCCCCTATGCCACTAAGGGCAACC
AGTGGGTGGGGTACGACGACCAGGACAGCGTCAAAATCAAGGTGCGGTACCTGAGAAGCAGGCAGCTGGC
AGGAGCCATGGTGTGGGCCCTGGACTTGGATGACTTCCAGGGCTCCTTCTGTGGCCAGAATCTGCGCTTC
CCTCTCACCAGTGCCATCAAGGATGCGCTTGCTGCGGCT
>Mus_musculus
ATGTACAAGCTGGTCTGCTACTTCACCAGCTGGTCCCAGTACCGGGAAGGCGTTGGAAGCTTCTTACCAG
ACGCCATCCAACCTTTCCTGTGCACCCACATCATCTACAGCTTTGCCAACATCAGCAGCGACAACATGCT
TAGCACATGGGAGTGGAATGACGAGTCGAACTATGACAAGCTGAATAAACTGAAGACCAGAAACACCAAC
CTGAAGACCCTCCTGTCTGTTGGAGGGTGGAAATTTGGCGAAAAAAGATTTTCCGAGATTGCCTCCAACA
CTGAGAGACGCACTGCTTTCGTCCGGTCGGTAGCCCCGTTCCTGCGTTCTTATGGCTTTGATGGGCTGGA
TCTCGCCTGGCTCTACCCTCGCTTAAGAGACAAGCAGTATTTCTCCACCCTGATCAAGGAACTGAATGCG
GAATTCACAAAGGAGGTCCAGCCAGGCAGAGAGAAACTCCTGCTCAGCGCAGCTTTGTCAGCAGGAAAGG
TGGCCATTGACACTGGCTATGACATCGCCCAGATAGCCCAACACCTGGATTTTATCAATCTCATGACCTA
CGATTTCCATGGAGTCTGGCGCCAAATCACAGGCCATCACAGCCCCCTCTTCCAAGGCCAGAAGGACACT
AGGTTTGACAGATACAGCAATGTGAACTATGCCGTGCAGTACATGATACGTCTGGGAGCCCAGGCCAGCA
AGCTACTGATGGGCATCCCCACCTTTGGGAAGAGCTTCACTCTGGCATCTTCTGAAAATCAGTTGGGAGC
TCCAATCTCAGGGGAAGGATTACCAGGCCGGTTCACCAAGGAGGCAGGGACCCTGGCCTACTACGAGATA
TGCGACTTCCTCAAAGGAGCTGAAGTACATCGACTCTCCAACGAGAAGGTTCCCTTCGCTACCAAGGGCA
ACCAGTGGGTGGGGTATGAGGACAAGGAGAGTGTCAAAAACAAGGTTGGGTTCCTGAAGGAGAAGAAGCT
GGCAGGAGCCATGGTGTGGGCACTGGATTTGGATGATTTCCAGGGCACCTGTCAGCCGAAGGAATTCTTC
CCGCTCACCAACGCCATCAAGGATGCCCTGGCT
>Rattus_norvegicus
ATGTACAAACTGGTCTGCTACTACACCAACTGGTCCCAGTACCGGGAAGGCAATGGGAGCTGCTTCCCAG
ATGCCCTCGACCATTCCCTGTGCACCCATATCATCTACAGCTTTGCCAACATCAGCAACAACAAGCTCAG
CACATCGGAGTGGAATGACGTAACCCTGTATGGCATGCTGAATACTCTCAAGACCAGAAACCCCAGACTG
AAGACACTGCTGTCTGTTGGAGGATGGAGCTTTGGCTCAGAAAGATTTTCCAGGATTGTCTCCAACGCTA
AGAGTCGCAAGACTTTCGTCCAGTCGGTAGCTCCCTTCCTGCGGACCTATGGCTTTGATGGACTGGATCT
CGCCTGGCTCTACCCGGGCCCGAAAGACAAGCAACATTTTACCACACTGATCAAGGAACTGAAGGCGGAA
TTCACAAAGGAAGTCCAGCCAGGCACAGAGAAACTCCTGCTCAGTGCTGCCGTGTCAGCAGGAAAGGTGA
CCCTTGACAGTGGCTATGATGTTGCCCAGATAGCCCAACACCTAGATTTCATTAATCTCATGACCTATGA
TTTCCATGGAACCTGGCGCCACACCACAGGACATCACAGCCCCCTCTTCCGAGGCCAGCAGGACACTGGG
CCTGACAGATTCAGCAATGTGGACTATGGTGTGGGGTACATGCTAAGGCTGGGAGCCCCCACCAACAAGC
TAGTGATGGGTATCCCCACCTTTGGAAAGAGCTTCACTCTGGCATCTTCTGAGAATCAAGTGGGAGCTCC
AATCACAGGGTCAGGATTACCAGGCCGCTACACCAAGGAGAAAGGGACCCTCGCCTACTACGAGATATGC
GACTTCCTCAGAGGAGCTGAAGTACATAGAATTCTTGGCCAGCAGGTTCCCTTTGCTACCAAGGGCAACC
AGTGGGTGGGGTATGATGACCCGGAGAGCGTCAAAAACAAGGTGAAGTACCTGAAGAACAAGCAGCTGGC
AGGAGCCATGGTGTGGGCAGTGGATTTGGATGATTTCCGGGGCTCCTTCTGTGGGCATAACGTACACTTC
CCGCTCACCAACGCCATCAAGGAGGCCCTGGCTGTGGCT
>Sus_scrofa
ATGTACAAGCTGGTTTGCTACTATACCAGCTGGTCTCAGTACCGGGAGGGTGATGGGAGCTGCTTCCCAG
ATGCCATCGACCCCTTCCTCTGCACCCACATCATCTACAGCTTTGCCAACATAAGCAACAATGAGATTGA
CACCTTGGAGTGGAATGATGTGACGCTCTATGACACACTGAACACACTCAAGAACAGGAACCCCAACCTG
AAGACCCTCCTGTCTGTTGGAGGATGGAACTTTGGTTCTCAAAGATTTTCCAAAATAGCCTCCAACACTC
AGAGTCGCAGGACTTTCATCAAGTCGGTGCCACCATTCCTGCGGACCCATGGCTTTGATGGACTGGACCT
AGCCTGGATCTCCCCTGGGCGGAGAGACAAGCGGCATCTCACCACTCTAGTCAAGGAGATGAAGGCTGAG
TTTGTAAGGGAAGCCCTGCCTGGAACAGAGCGGCTCCTGCTCAGCGGAGCGGTGTCTGCAGGGAAGGTCG

CCATTGACAGAGGCTATGACATCGCCCAGATATCTCAACACCTGGACTTCATCAGCCTTTTGACCTATGA
CTTCCACGGTGCCTGGCGCCAAACTACAGGACACCACAGTCCCCTGTTCCGAGGCCAGGGGGATGCAAGT
TCCGACAGATTCAGCAATGCAGACTATGCTGTGAGCTATGTGCTGAGGCTGGGGGCCCCAGCCAATAAGC
TGGTGATGGGTATCCCCACTTTTGGGAGGAGCTTCACTTTGGCCTCTTCCAAGACAGACGTGGGAGCCCC
GGCATCAGGGCCAGGAATACCAGGCCGGTTCACCAAGGAGAAAGGGATCCTTGCCTACTATGAGATCTGT
GACTTCCTCCAAGGAGCCACAGTCCGTAGACCCCTTGGCCAGCAGGTCCCCTATGCCACCAAGGGCAACC
AGTGGGTGGGGTATGATGACCAGGAGAGCGTCAAAAACAAGGCAAAGTACCTGAAGAGCAGGCAGCTGGC
TGGTGCTATGGTGTGGACCCTGGACTTGGATGACTTTCGGGGCAACTTCTGTGGGCAGAACCTACGCTTT
CCTCTCACCAGTGCCATCAAGGATGTGCTTGCTGCGGCG

Supplementary data set 2 for evolutionary analysis in CHIT1 CatD among primates.

```
>Homo_sapiens
ATGGCAAAACTGGTCTGCTACTTCACCAACTGGGCCCAGTACAGACAGGGGGAGGCTCGCTTCCTGCCCA
AGGACTTGGACCCCAGCCTTTGCACCCACCTCATCTACGCCTTCGCTGGCATGACCAACCACCAGCTGAG
CACCACTGAGTGGAATGACGAGACTCTCTACCAGGAGTTCAATGGCCTGAAGAAGATGAATCCCAAGCTG
AAGACCCTGTTAGCCATCGGAGGCTGGAATTTCGGCACTCAGAAGTTCACAGATATGGTAGCCACGGCCA
ACAACCGTCAGACCTTTGTCAACTCGGCCATCAGGTTTCTGCGCAAATACAGCTTTGACGGCCTTGACCT
TGACTGGGAGTACCCAGGAAGCCAGGGGAGCCCTGCCGTAGACAAGGAGCGCTTCACAACCCTGGTACAG
GACTTGGCCAATGCCTTCCAGCAGGAAGCCCAGACCTCAGGGAAGGAACGCCTTCTTCTGAGTGCAGCGG
TTCCAGCTGGGCAGACCTATGTGGATGCTGGATACGAGGTGGACAAAATCGCCCAGAACCTGGATTTTGT
CAACCTTATGGCCTACGACTTCCATGGCTCTTGGGAGAAGGTCACGGGACATAACAGCCCCCTCTACAAG
AGGCAAGAAGAGAGTGGTGCAGCAGCCAGCCTCAACGTGGATGCTGCTGTGCAACAGTGGCTGCAGAAGG
GGACCCCTGCCAGCAAGCTGATCCTTGGCATGCCTACCTACGGACGCTCCTTCACACTGGCCTCCTCATC
AGACACCAGAGTGGGGGCCCCAGCCACAGGGTCTGGCACTCCAGGCCCCTTCACCAAGGAAGGAGGGATG
CTGGCCTACTATGAAGTCTGCTCCTGGAAGGGGGCCACCAAACAGAGAATCCAGGATCAGAAGGTGCCCT
ACATCTTCCGGGACAACCAGTGGGTGGGCTTTGATGATGTGGAGAGCTTCAAAACCAAGGTCAGCTATCT
GAAGCAGAAGGGACTGGGCGGGGCCATGGTCTGGGCACTGGACTTAGATGACTTTGCCGGCTTCTCCTGC
AAC
>Lemur_catta
ATGGCAAAACTGGTCTGCTACTTCACCAACTGGTCCCAGTACAGAGAGGGGGCAGCTCGCTTCTTGCCCA
AGGACGTGAACCCCGACCTGTGCACCCACCTTGTCTACGCCTTTGCCGGCATGAACAACCTCCAGCTCAG
CACCACCGAGTGGAGTGACGAGGCTCTCTACCAGCAGTTCAACGGGCTGAAGAAGATGAATCCCAGGCTG
AAGACTCTGTTAGCCATCGGGGGCTGGAACTTTGGCACTCAGAAGTTCACAGAGATGGTGGCCACAGCCA
GCAACCGCCAGACTTTCGTCAGCTCAGCCATTGCGTTTCTGCGCAAATACGGCTTTGATGGCCTTGACCT
TGACTGGGAGTTCCCAGGAAGCCGGGGGAGCCCTGCCGTGGACAAGGAGCGCTTCACGGCCCTGGTGCAG
GACTTGGCCGACGCCTTCCAGAAAGAAGCCCAGACCTCGAGGAAGGAACGACTCCTCCTGAGTGCAGCCG
TCCCAGCCGGGCAAGCCGTTGTGGAGGCCGGATATGAGGTGGACAAAATTGCCCAGAACCTGGATTTCAT
CAGCCTTATGGCCTATGACCTCCACGGCTCTTGGGAGAAGGTCACAGGACATAACAGCCCCCTCTACAAG
AGGCAGGGAGAGAGTGGGGCGGCGGCCCGGCTCAACGTGGATGCTGCCGTACAGCTGTGGCTGAAGAAGG
GGACCACCGCCAACAAACTGATCCTCGGCATGCCCACCTACGGACGATCCTTCACTCTGGCCTCCTCCTC
AGACACGGGGGTGGGGGCCCCAGCCACAGGGCCTGGCAGCCCCGGCCGCTTCACCAAGGAAGCCGGGGTG
CTGGCTTACTATGAGGTCTGCTCCTGGCAGGGGGCCACCGTGCACAGGATCCAGGACCAGAAGGTGCCCT
ACATTGTGCAGAACAACCAGTGGGTGGGCTTTGATGACGTGGACAGCTTCAAAGCCAAGGTCGCCTATCT
GAAGCAGAAGGGGCTGGGTGGGGCCATGGTCTGGACACTGGCCCTGGACGACTTTGCTGGCTCCTTCTGC
AAC
>Propithecus_coquereli
ATGGCAAAACTGGTCTGCTACTTCACCAACTGGTCCCAGTACAGAGAGGGGGCAGCTCGCTTCTTGCCCA
AGGACGTGAACCCCAACCTGTGTACCCACCTTGTCTACGCCTTTGCCGGCATGAACAACCACCAGCTCAG
CACCATCGAGTGGAATGACGAGGCTCTCTACCAGGCGTTCAACGGCCTGAAGAAAATGAATCCCAAGCTG
AAGACTCTGTTAGCCATTGGGGGCTGGAGCTTCGGCACTCAGAAGTTCACAGAGATGGTGGCCACAGCCA
ACAACCGCCAGACTTTCATCAGCTCAGCCATCGAGTTTCTGCGCAAATACGGCTTTGATGGCCTTGATCT
TGACTGGGAGTTCCCAGGAAGCCGGGGGAGCCCTGCCGTGGACAAGGAGCGCTTCACGGCCCTGGTGCAG
GACTTGGCCAGCACATTCCAGAAAGAAGCCCAGACCTCAAGGAAGGAGCGACTCCTCCTGAGTGCAGCCG
TCCCAGCCGGGCCAGCCCTTGTGGATGCTGGATATGAGGTGGACAAAATTGCCCAGAACCTGGATTTCAT
CAGCCTTATGGCCTATGACCTCCACGGCTCCTGGGAGAAGGTCACAGGACATAACAGCCCCCTCTACAAG
AGGCAGGGGGAGAGTGGCGCGGCGGCCCAACTCAATGTGGATGCTGCCGTACAGCTGTGGCTGAAGAAGG
GGACCCCCGCCAACAAACTGATACTTGGCATGCCCACCTACGGACGATCCTTCACTCTGGCCTCCTCATC
AGACACCAGGGTGGGGGCCCCAGCCACAGGGCCTGGCAGCCCTGGCCGCTTCACCAAGGAAGCAGGGGTG
CTGGCTTACTATGAGGTCTGCTCCTGGAAGGGGGCCACTGTGCACAGAACCCAGGACCAGAAGGTGCCCT
ACATTGTCCAGAACAACCAGTGGGTGGGCTTTGATGACGTGGAGAGCTTCAAAGCCAAGGTCGCCTATCT
GAAGCAGAAGGGCCTGGGTGGGGCCATGGTCTGGGCACTGGCCTTGGACGACTTTGCTGGCTTCTTCTGC
AAC
>Microcebus_murinus
ATGGCAAAACTGGTCTGCTACTTCACCAACTGGTCCCAGTACAGAGAGGGGGCAGCTCGCTTCCTGCCCA
AGGATGTGGACCCCAACCTGTGCACGCACCTCGTCTACGCCTTTGCTGGCATGAACAACCACCAGCTCAG
CACCCTGGAGTGGAACGATGAGGCTCACTACCAGCAGTTCAACGGCCTGAAGAAGATGAATCCCAAGCTG
AAGACCCTGTTGGCCGTTGGGGGCTGGAGCTTCGGCACTCAGAAGTTCACAGAGATGGTGGCCACAGCCG
ACACCCGCCAGACTTTTACCCGCTCAGCCGTCGAGTTTCTGCGCAAATACGGCTTTGACGGACTTGACCT
CGACTGGGAGTTCCCAGGAGGCCGGGGGAGCCCTGCCGTGGACAAGCAGCGCTTCACGGCCCTGGTGCAG
GACTTGGCCAGCGCCTTCCAGAAAGAAGCCCAGACCTCCAGGAAGGAGCGACTCCTCCTGAGTTCAGCCG
TCTCTGCCGGGCCAGCCATCGTGGACGCTGGATATGAGGTGGACAAAATTGCCCAGAACCTGGACTTCAT
CAGCCTTATGGCCTATGACCTCCACGGCTCCTGGGAGAAGGTCACAGGGCACAACAGCCCCCTCTACAAG
AGGCAGGGGGAGAGCGGTGCGGCGGCCCAGCACAACGTGGACGCTGCCGTACAGCTGTGGCTGAACAAGG
GGACCCCCGCCAACAAACTGATCCTCGGCATGCCCACCTACGGACGATCCTTCACTCTGGCCTCCTCCTC
```

GGACACCGGGGTGGGGGCCCCAGCCACAGGGCCTGGCAGCCCTGGCAGCTTCACCAAGGAAGCAGGGGTG
CTGGCTTACTATGAGGTGTGCTCCTGGCAGGGGGCCACTGTGCACAGAATCCAGGACCAGAAGGTGCCCT
ACATTGTCCAGAACAACCAGTGGGTGGGCTTTGATGACGTGGAGAGCTTCAAAGCCAAGGTCGCCTATCT
GAAGCAGAAGGGCCTGGGTGGGGCCATGGTCTGGGCACTGGCCTTGGACGACTTCGCTGGCTTCTTCTGC
AAC
>Otolemur_garnettii
ATGGCAAAATTGATCTGCTACTTCACCAACTGGGCCCAATACAGAGAGGGGGCAGCTCGCTTCTTGCCCA
AGGATGTGGACCCCAGCCTGTGCACCCACCTCATCTATGCCTTTGCCGGCATGAACAATCACCAGCTCAG
CACCATTGAATGGAATGACGAGGCCCTCTACCAGGAGTTCAACGGCCTGAAGAAGATGAATCCTAAGCTG
AAGACCCTGTTAGCCTTAGGGGGCTGGAACTTTGGCACTCAGAAGTTCACAGATATGGTGGCCACTCCCA
ACAACCGCCAGATTTTCATCAGCTCAGCCATCAAGTTTCTGCGCAAATATGGTTTTGATGGCCTTGACCT
TGACTGGGAATACCCAGGAAGCCGGGGTAGCCCTGCTGTGGACAAAGAGCGCTTCACAGCCCTGGTGCAG
GAGTTGGCCAACGCCTTCCAGCAGGAAGCCCAGACCTCAAACAAGGATCGACTCCTCCTGAGTGCAGCTG
TCCCGGCCGGGCAAGCCTATGTGGATGCTGGATATGAAGTGGACAAAATTGCCCAGAACTTGGATTTCAT
CAGCCTTATGGCCTATGACCTCCATGGCTCCTGGGAGCAGGTCACAGGACATAACAGCCCCCTCTATAAG
AGGCAGGGAGAGAGTGGTGCCGCAGCCAAACTCAATGTGGACGCTGCCGTACAATACTGGCTGAAGAGGG
GGACTCCTGCCGATAAACTGATCCTGGGCATGCCTACCTACGGACGATCCTTCACTCTGGCTTCCTCATC
AGACACCAGAGTGGGGGCCCCAGCCACAGGGCCTGGCACCCCTGGCCCCTTCACCAAGGAAGGAGGGTTT
CTGGCTTACTATGAGGTCTGCTCCTGGAAGGGGGCCACTGAACGCAGAATCCAGGACCAGAAGGTGCCCT
ACGTTTTCCAGAACAACCAGTGGGTAGGCTTTGATGATGTAGACAGCTTCAAAGCCAAGGCTGCCTATCT
GAAGCAGAAGGGACTGGCTGGGGCCATGGTCTGGGCACTGGACTTGGACGACTTTGCTGGCTCCTTCTGC
AGC
>Aotus_nancymaae
ATGGCAAAACTGGTCTGCTACTTCACCAACTGGGCCCAGTACAGACAGGGGTCAGCTCGCTTCCTGCCCA
AGGACGTGGACCCCAGCCTGTGCACCCATCTTATCTACGCCTTCGCCGGCATGACCAGTCACCAGCTGAG
CACCACTGAGTTGAATGACGAGACTCTCTACCAGGAGTTCAACGGCCTGAAGAAGATGAATCCCAAGCTG
AAGACCCTGTTGGCCATCGGAGGCTGGAATTTCGGCACTCAGAAGTTCACAGATATGGTAGCCACGACCA
ACAACCGTCAGACCTTTGTCAACTCAGCCATCAGGTTTCTGCGCAAATATGGCTTTGATGGCCTTGACCT
TGACTGGGAGTACCCAGGGAGCCGGGGGAGCCCTGCCATAGACAAAGAGCGCTTCACAGCTCTGGTGCAG
GACTTGGCCAACGCCTTCCAGAAGGAAGCTCAGACCTCAGAGAAGGAACGCCTCCTTCTGAGTGCAGCGG
TTCCAGCTGGGCGAACCTATGTGGATGCTGGATACGAGGTGGACAAAATTGCCCAGAACCTGGACTTTAT
CAACCTTATGGCCTATGACTTCCATGGCTCTTGGGAGAAGATCACGGGACACAACAGCCCCCTCTACAAG
AGGCAAGGGGAGAGCGGTGCAGCGGCCAGCCTCAATGTGGATGCTGCCGTGCAACTGTGGCTGCAGAAGG
GGACCCCTGCCAGCAAGCTGATCCTTGGCATGCCTACCTATGGACGTTCCTTCACCCTGGCCTCCTCGTC
AGACACCAGAGTGGGGGCCCCAGTCACAGGGTCTGGTACCCCTGGCCCCTTCACCAAGGAAGGAGGGATG
CTGGCCTACTATGAGGTCTGCTCCTGGAAGGGGGCCACCAAGCAGAGAATCCAGGACCAGAAGGTGCCCT
ACATCTTCCGGGACAACCAGTGGGTGGGCTTTGATGATGCGGAGAGCTTCAAAGCCAAGGTCAGCTATCT
GAAGCAGAAAGGACTGGGCGGGGCCATGGTCTGGGCACTGGACTTAGATGACTTTGCCGGCTTCTCCTGC
AAC
>Hylobates_moloch
ATGGCAAAACTGGTCTGCTACTTCACCAACTGGGCCCAGTACAGACAGGGGGAGGCTCGCTTCCTGCCCA
AGGACGTGGACCCCAGCCTTTGCACCCACCTCATCTACGCCTTCGCTGGCATGACCAACCACCAGCTGAG
CACCATTGAGTGGAATGATGAGACTCTGTACCAGGAGTTCAATGGCCTGAAGAAGATGAATCCCAAGCTG
AAGACCCTGTTAGCCATCGGAGGCTGGAATTTCGGCACTCAGAAGTTCACAGATATGGTAGCCACGGCCA
ACAACCGTCAGACCTTTGTCAACTCAGCCATCAGGTTTCTGCGCAAATACAGCTTTGACGGCCTTGACCT
TGACTGGGAGTACCCAGGAAGCCGGGGGAGCCCTGCCGTAGACAAGGAGCGCTTCACAGCCCTGGTACAG
GACTTGGCCAATGCCTTCCAGCAGGAAGCCCAGACCTCAGGGAAGGAACGCCTTCTTCTGAGTGCAGCGG
TTCCAGCTGGGCGGACCTATGTGGATGCTGGATACGAAGTGGACAAAATCGCCCAGAACCTGGATTTTGT
CAACCTTATGGCCTACGACTTCCACGGCTCTTGGGAGAAGGTCACGGGACATAACAGCCCCCTCTACAAG
AGGCAAGAGGACAGTGGTGCAGCAGCCAGCCTCAACGTGGATGCTGCTGTGCAACTGTGGCTGCAGAAGG
GGACCCCTGCCAGCAAGCTGATCCTTGGCATGCCTACCTACGGACGCTCCTTCACCCTGGCCTCCTCATC
AGACACCAGAGTGGGGGCCCCAGCCACAGGGTCTGGCACTCCCGGCCCCTTCACCAAGGAAGGAGGCATG
CTGGCCTACTATGAGGTCTGCTCCTGGAAGGGGGCCACCAAACAGAGAATCAAGGACCAGGAGGTGCCCT
ACATCTTCCGGGACAACCAGTGGGTGGGCTTTGATGATGTGGAGAGCTTCAAAACCAAGGTCAGCTATCT
GAAGCAGAAGGGACTGGGCGGGGCCATGGTCTGGGCACTGGACTTAGATGACTTTGCCAGCTTCTCCTGC
AAC
>Papio_anubis
ATGGCAAAACTGGTCTGCTACTTCACCAACTGGGCCCAGTACAGACAGGGGGAGGCTCGCTTCCTGCCCA
AGGATGTGGACCCCAGCCTTTGCACCCACCTCATCTACGCCTTCGCTGGCATGACCAACCACCAGCTGAG
CACCATTGAGTGGAATGACGAGACTCTCTACCAGGAGTTCAATGGCCTGAAGAAGATGAATCCCAAGCTG
AAGACCCTGTTAGCCATCGGAGGCTGGAATTTCGGCACTCAGAGGTTCACAGATATGGTAGCCACGGCCA
ACAACCGTCAGACCTTCGTCAACTCAGCCATCAGATTTCTGCGCAAATACGGCTTTGATGGCCTTGACCT
TGACTGGGAGTACCCAGGAAGCCGGGGGAGCCCTGCCATAGACAAGGAACGCTTCACAGCCCTGGTGCAG
GATTTGGCCAACACCTTCCAGCAGGAAGCCCAGACCTCAGGGAAGGAACGCCTCCTTCTGAGTGCAGCAG
TTCCAGCTGGGCGGACCTATGTGGATGCTGGATACGAGGTGGACAAAATCGCCAGGAACCTGGATTTTGT

CAACCTTATGGCCTACGACTTCCATGGCTCTTGGGAGAAGGTCACGGGACATAATAGCCCCCTCTACAAG
AGGCAAGAGGAGAGTGGTGCAGCGGCCAGCCTCAACGTGGATGCTGCTGTGCAAATGTGGCTGCAGAAGG
GGACCCCTGCCAGCAAGCTGATCCTTGGCATGCCTACCTACGGACGCTCCTTCACCCTGGCCTCCCCGTC
AGACACCAGAGTGGGGGCCCCAGCCACAGGGTCTGGCACTCCTGGCCCCTTCACCAAGGAAGGAGGGATA
TTGGCCTACTATGAGGTCTGCTCCTGGAAGGGGGCCACCAAACAGAGAATCCAGGACCAGGAGGTGCCCT
ACATCTTCCGGGACAACCAGTGGGTGGGCTTTGATGATGTGGAGAGCTTCAAAACCAAGGTCAGCTATCT
GAAGCAGAAGGGACTGGGCGGGGCGATGGTCTGGGCACTGGATTTAGATGACTTTGCCGGCTTCTCCTGC
GAC
>Macaca_mulatta
ATGGCAAAACTGGTCTGCTACTTCACCAACTGGGCCCAGTACAGACAGGGGGAGGCTCGCTTCCTGCCCA
AGGACGTGGACCCCAGCCTTTGCACCCACCTCATCTACGCCTTCGCTGGCATGACCAACCACCAGCTGAG
CACCATTGAGTGGAATGACGAGACTCTCTACCAGGAGTTCAATGGCCGGAAGAAGATGAATCCCAAGCTG
AAGACCCTGTTAGCCATCGGAGGCTGGAATTTCGGCACTCAGAGGTTCACAGATATGGTAGCCACGGCCA
ACAACCGTCAGACCTTCGTCAACTCAGCCATCAGATTTCTGCGCAAATACGGCTTTGATGGCCTTGACCT
TGACTGGGAGTACCCAGGAAGCCGGGGGAGCCCTGCCATAGACAAGGAACGCTTCACAGCCCTGGTGCAG
GACTTGGCCAACGCCTTCCAGCAGGAAGCCCAGACCTCAGGGAAGGAACGCCTCCTTCTGAGTGCAGCGG
TTCCAGCTGGGCGGACCTATGTGGATGCTGGATACGAGGTGGACAAAATCGCCAGGAACCTGGATTTTGT
CAACCTTATGGCCTACGACTTCCATGGCTCTTGGGAGAAGGTCACGGGACATAATAGTCCCCTCTACAAG
AGGCAAGAGGAGAGTGGTGCAGCGGCCAGCCTCAACGTGGATGCTGCTGTGCAAATGTGGCTGCAGAAGG
GGACCCCTGCCAGCAAGCTGATCCTTGGCATGCCTACCTACGGACGCTCCTTCACCCTGGCCTCCCCCTC
AGACACCAGAGTGGGGGCCCCAGCCACAGGGTCTGGCACTCCTGGCCCCTTCACCAAGGAAGGAGGGATG
TTGGCCTACTATGAGGTCTGCTCCTGGAAGGGGGCCACCAAACAGAGAATCCAGGACCAGGAGGTGCCCT
ACATCTTCCGGGACAACCAGTGGGTGGGCTTTGATGATGTGGAGAGCTTCAAAACCAAGGTCAGCTATCT
GAAGCAGAAGGGACTGGGCGGGGCGATGGTCTGGGCACTGGATTTAGATGACTTTGCCGGCTTCTCCTGC
GAC
>Theropithecus_gelada
ATGGCAAAACTGGTCTGCTACTTCACCAACTGGGCCCAGTACAGACAGGGGGAGGCTCGCTTCCTGCCCA
AGGACGTGGACCCCAGCCTTTGCACCCACCTCATCTACGCCTTCGCTGGCATGACCAACCACCAGCTGAG
CACCATTGAGTGGAATGACGAGACTCTCTACCAGGAGTTCAATGGCCTGAAGAAGATGAATCCCAAGCTG
AAGACCCTGTTAGCCATCGGAGGCTGGAATTTCGGCACTCAGAGGTTCACAGATATGGCAGCCACGGCCA
ACAACCGTCAGACCTTCGTCAACTCAGCCATCAGGTTTCTGCGTAAATACGGCTTTGATGGCCTTGACCT
TGACTGGGAGTACCCAGGAAGCCGGGGGAGCCCTGCCATAGACAAGGAACGCTTCACAGCCCTGGTGCAG
GACTTGGCCAACGCCTTCCAGCAGGAAGCCCAGACCTCAGGGAAGGAACGCCTCCTTCTGAGTGCAGCGG
TTCCAGCTGGGCAGACCTATGTGGATGCTGGATACGAGGTGGACAAAATCGCCAGGAACCTGGATTTTGT
CAACCTTATGGCCTACGACTTCCATGGCTCTTGGGAGAAGGTCACAGGACATAATAGCCCCCTCTACAAG
AGGCAAGAGGAGAGTGGTGCAGCGGCCAGCCTCAACGTGGATGCTGCTGTGCAAATGTGGCTGCAGAAGG
GGACCCCTGCCAGCAAGCTGATCCTTGGCATGCCTACCTACGGACGCTCCTTCACCCTGGCCTCCCCGTC
AGACACCAGAGTGGGGGCCCCAGCCACAGGGTCTGGCACTCCTGGCCCCTTCACCAAGGAAGGAGGGATA
TTGGCCTACTATGAGGTCTGCTCCTGGAAGGGGGCCACCAAACAGAGAATCCAGGACCAGGAGGTGCCCT
ACATCTTCCGGGACAACCAGTGGGTGGGCTTTGATGATGTGGAGAGCTTCAAAACCAAGGTCAGCTATCT
GAAGCAGAAGGGACTGGGCGGGGCGATGGTCTGGGCACTGGATTTAGATGACTTTGCCGGCTTCTCCTGC
GAC
>Macaca_fascicularis
ATGGCAAAACTGGTCTGCTACTTCACCAACTGGGCCCAGTACAGACAGGGGGAGGCTCGCTTCCTGCCCA
AGGATGTGGACCCCAGCCTTTGCACCCACCTCATCTACGCCTTTGCTGGCATGACCAACCACCAGCTGAG
CACCATTGAGTGGAATGACGAGACTCTCTACCAGGAGTTCAATGGCCGGAAGAAGATGAATCCCAAGCTG
AAGACCCTGTTAGCCATCGGAGGCTGGAATTTCGGCACTCAGAGGTTCACAGATATGGTAGCCACGGCCA
ACAACCGTCAGACCTTCGTCAACTCAGCCATCAGATTTCTGCGCAAATACGGCTTTGATGGCCTTGACCT
TGACTGGGAGTACCCAGGAAGCCGGGGGAGCCCTGCCATAGACAAGGAACGCTTCACAGCCCTGGTGCAG
GACTTGGCCAACGCCTTCCAGCAGGAAGCCCAGACCTCAGGGAAGGAACGCCTCCTTCTGAGTGCAGCGG
TTCCAGCTGGGCGGACCTATGTGGATGCTGGATACGAGGTGGACAAAATCGCCAGGAACCTGGATTTTGT
CAACCTTATGGCCTACGACTTCCATGGCTCTTGGGAGAAGGTCACGGGACATAATAGTCCCCTCTACAAG
AGGCAAGAGGAGAGTGGTGCAGCGGCCAGCCTCAACGTGGATGCTGCTGTGCAAATGTGGCTGCAGAAGG
GGACCCCTGCCAGCAAGCTGATCCTTGGCATGCCTACCTACGGACGCTCCTTCACCCTGGCCTCCCCCTC
AGACACCAGAGTGGGGGCCCCAGCCACAGGGTCTGGCACTCCTGGCCCCTTCACCAAGGAAGGAGGGATG
TTGGCCTACTATGAGGTCTGCTCCTGGAAGGGGGCCACCAAACAGAGAATCCAGGACCAGGAGGTGCCCT
ACATCTTCCGGGACAACCAGTGGGTGGGCTTTGATGATGTGGAGAGCTTCAAAACCAAGGTCAGCTATCT
GAAGCAGAAGGGACTGGGCGGGGCGATGGTCTGGGCACTGGATTTAGATGACTTTGCCGGCTTCTCCTGC
GAC
>Chlorocebus_sabaeus
ATGGCAAAACTGGTCTGCTACTTCACCAACTGGGCCCAGTACAGACAGGGGGAGGCTCGCTTCCTGCCCA
AGGACGTGGACCCCAGCCTTTGCACCCACCTCATCTATGCCTTTGCTGGCATGACCAACCACCAGCTGAG
CACCATTGAGTGGAATGACGAGACTCTCTACCAGGAGTTCAATGGCCTGAAGAAGATGAATCCCAAGCTG
AAGACCCTGTTAGCCATCGGAGGCTGGAATTTCGGCACTCAGAGGTTCACAGATATGGTAGCCACGGCCA
ACAACCGTCAGACCTTCGTCAACTCAGCCATCAGATTTCTGCGCAAATACGGCTTTGATGGCCTTGACCT

TGACTGGGAATACCCAGGAAGCCGGGGGAGCCCTGCCATAGACAAGGAACGCTTCACAGCCCTGGTGCAG
GACTTGGCCAACGCCTTCCAGCAGGAAGCCCAGACCTCAGGGAAGGAACGCCTCCTTCTGAGTGCAGCAG
TTCCAGCTGGGCAGACCTATGTGGATGCTGGATACGAGGTGGACAAAATCGCCAGGAACCTGGATTTTGT
CAACCTTATGGCCTACGACTTCCATGGCTCTTGGGAGAAGGTCACGGGACATAATAGCCCCCTCTACAAG
AGGCAAGAGGAGAGTGGTGCAGCGGCCAGCCTCAACGTGGATGCTGCTGTGCAAATGTGGCTGCAGAAGG
GGACCCCTGCCAGCAAGCTGATCCTTGGCATGCCTACCTACGGACGCTCCTTCACCCTGGCCTCCCCGTC
AGACACCAGAGTGGGGGCCCCAGCCACAGGGTCTGGCACTCCTGGCCCCTTCACCAAGGAAGGAGGGATA
TTGGCCTACTATGAGGTCTGCTCCTGGAAGGGGGCCACCAAACAGAGAATCCAGGACCAGGAGGTGCCCT
ACATCTTCCGGGACAACCAGTGGGTGGGCTTTGATGATGTGGAGAGCTTCAAAACCAAGGTCAGCTATCT
GAAGCAGAAGGGACTGGGCGGGGCGATGGTCTGGGCATTGGATTTAGATGACTTTGCCGGCTTCTCCTGC
GAC
>Cercocebus_atys
ATGGCAAAACTGGTCTGCTACTTCACCAACTGGGCCCAGTACAGACAGGGGGAGGCTCGCTTCCTGCCCA
AGGATGTGGACCCCAGCCTTTGCACCCACCTCATCTACGCCTTCGCTGGCATGACCAACCACCAGCTGAG
CACCATTGAGTGGAATGACGAGACTCTCTACCAGGAGTTCAATGGCCGGAAGAAGATGAATCCCAAGCTG
AAGACCCTGTTAGCCATCGGAGGCTGGAATTTCGGCACTCAGAGGTTCACAGATATGGTAGCCACGGCCA
ACAACCGTCAGACCTTCGTCAACTCAGCCATCAGGTTTCTGCGCAAATACGGCTTTGATGGCCTTGACCT
TGACTGGGAGTACCCAGGAAGCCGGGGGAGCCCTGCCATAGACAAGGAACGCTTCACAGCCCTGGTGCAG
GACTTGGCCAACGCCTTCCAGCAGGAAGCCCAGACCTCAGGGAAGGAACGCCTCCTTCTGAGTGCAGCGG
TTCCAGCTGGGCGGACCTATGTGGATGCTGGATACGAGGTGGACAAAATCGCCAGGAACCTGGATTTTGT
CAACCTTATGGCCTACGACTTCCATGGCTCTTGGGAGAAGGTCACGGGACATAATAGCCCCCTCTACAAG
AGGCAAGAGGAGAGTGGTGCAGCGGCCAGCCTCAACGTGGATGCTGCTGTGCAAATGTGGCTGCAGAAGG
GGACCCCTGCCAGCAAGCTGATCCTTGGCATGCCTACCTACGGACGCTCCTTCACCCTGGCCTCCCCGTC
AGACACCAGAGTGGGGGCCCCAGCCACAGGGTCTGGCACTCCTGGCCCCTTCACCAAGGAAGGAGGGATA
TTGGCCCACTATGAGGTCTGCTCCTGGAAGGGGGCCACCAAACAGAGAATCCAGGACCAGGAGGTGCCCT
ACATCTTCCGGGACAACCAGTGGGTGGGCTTTGATGATGTGGAGAGCTTCAAAACCAAGGTCAGCTATCT
GAAGCAGAAGGGACTGGGCGGGGCGATGGTCTGGGCACTGGATTTAGATGACTTTGCCGGCTTCTCCTGC
GAC
>Rhinopithecus_roxellana
ATGGCAAAACTGGTCTGCTACTTCACCAACTGGGCCCAGTACAGACAGGGGGAGGCTCGCTTCCTGCCCA
AGGACGTGGACCCCAGCCTTTGCACCCACCTCATCTATGCCTTTGCTGACATGACCAACCACCAGCTGAG
CACCATTGAGTGGAATGACGAGACTCTCTATCAGGAGTTCAATGGCCTGAAGAAGATGAATCCCAAGCTG
AAGACCCTGTTAGCCATCGGAGGCTGGAATTTCGGCACTCAGAGGTTCACAGATATGGTAGCCACGGCCA
ACAACCGTCAGACCTTCGTCAACTCAGCCATCAGGTTTCTGCGCAAATACGGCTTTGATGGCCTTGACCT
TGACTGGGAGTACCCAGGAAGCCGGGGAAGCCCTGCCATAGACAAGGAACGCTTCACAGCCCTGGTGCAG
GACTTGGCCAATGCCTTCCAGCAGGAAGCCCAGACCTCAGGGAAGGAATGCCTCCTTCTGAGTGCAGCGG
TTCCAGCTGGGCAGACCTATGTGGATGCTGGATATGAGGTGGACAAAATCGCCAGGAACCTGGATTTTGT
CAACCTTATGGCCTATGACTTCCATGGCTCTTGGGAGAAGGTCACGGGACATAACAGCCCCCTCTACAAG
AGGCAAGAGGAGAGTGGTGCAGCGGCCAGCCTCAACGTGGATGCTGCTGTGCAACAGTGGCTGCAGAAGG
GGACCCCTGCCAGCAAGCTGATCCTTGGCATGCCTACCTATGGACGCTCCTTCACCCTGGCCTCCTCGTC
AGACACCAGAGTGGGGGCCCCAGCCACAGGGTCTGGCACTCCTGGCCCCTTCACCAAGGAAGGAGGGATG
TTGGCCTACTATGAGGTCTGCTCCTGGAAGGGGGCCACCAAACAGAGAATCCAGGACCAGGAGGTGCCCT
ACATCTTTCGGGACAACCAGTGGGTGGGCTTTGATGATGTGGAGAGCTTCAAAACCAAGGTCAGCTATCT
GAAGCAGAAGGGACTGGGCGGGGCGATGGTCTGGGCACTGGATTTAGATGACTTTGCCGGCTTCTCCTGC
GAC
>Colobus_angolensis
ATGGCAAAACTGGTCTGCTACTTCACCAACTGGGCCCAGTACAGACAGGGGGAGGCTCGCTTCCTGCCCA
AGGATGTGGACCCCAGCCTTTGCACCCACCTCATCTACGCCTTTGCTGGCATGACCAACCACCAGCTGAG
CACCATTGAGTGGAATGACGAGACTCTCTACCAGGAGTTCAATGGCCTGAAGAAGATGAATCCCAAGCTG
AAGACCCTGTTAGCCATCGGAGGCTGGAATTTCGGTACTCAGAAGTTCACAGATATGGTTGCCACGGCCA
ACAACCGTCAGACCTTCGTCAACTCAGCCATCAGGTTTCTGCGCAAATACGGCTTTGATGGCCTTGACCT
TGACTGGGAGTACCCAGGAAGCCGGGGGAGCCCTGCCATAGACAAGGAACGCTTCACAGCCCTGGTGCAG
GACTTGGCCAACGCCTTCCAGCAGGAAGCCCAGACCTCAGGGAAGGGACGTCTCCTTCTGAGTGCAGCGG
TTCCAGCTGGGCAGACCTATGTGGATGCTGGATATGAGGTGGACAAAATCGCCAGGAACCTGGATTTTGT
CAACCTTATGGCCTATGACTTCCATGGCTCTTGGGAGAAGGTCACGGGACATAATAGCCCCCTCTACAAG
AGGCAAGAGGAGAGTGGTGCAGCGGCCAGCCTCAACGTGGATGCTGCTGTGCAACAGTGGCTGCAGAAGG
GGACCCCTGCCAGCAAGCTGATCCTTGGCATGCCTACCTATGGACGCTCCTTCACCCTGGCCTCCTCGTC
AGACACCAGAGTGGGGGCCCCAGCCACAGGGTCTGGCACTCCTGGCCCCTTCACCAAGGAAGGAGGGATG
TTGGCCTACTATGAGGTCTGCTCCTGGAAGGGGGCCACCAAACAGAGAATCCAGGACCAGGAGGTGCCCT
ACATCTTCAGGGACAACCAGTGGGTGGGCTTTGATGATGTGGAGAGCTTCAAAACCAAGGTCAGCTATCT
GAAGCAGAAGGGACTGGGCGGGGCGATGGTCTGGGCACTGGATTTAGATGACTTTGCCGGCTTCTCCTGT
GAC
>Carlito_syrichta
ATGACAAAACTGGTCTGCTACTTCACCAACTGGGCTCAGTACAGACAAGAGGCGGCTCGCTTCCTGCCCA
AGGATGTGGACCCCAACCTTTGCACCCACCTCATCTACGCCTTTGCCGGCATGAACAATCACCAACTCAA

CACCATCGAGTGGAATGATGAGGTTCTCTACCAGGAGTTCAACGGCCTGAAGAGGACGAATCCCAAGCTG
AAGACTCTGTTAGCCATCGGGGGCTGGAACTTCGGCACTCAGAAGTTCACTGATATGGTGGCCTCGGCCA
ACAATCGTCAGATCTTTGTCAACTCAGCCATCAAGTTTCTGCGCAAATATGGCTTTGATGGCCTTGACCT
TGACTGGGAGTATCCAGGAAGCCGGGGGAGCCCTGCCATAGATAAGGAGCATTTCACAGCCCTGGTGCAG
GACTTGGCCAGCGCTTTCCAGCAGGAAGCCCAGTCCTCAAAGAAGGAACGACTCCTCTTGAGTGCAGCTG
TCCCAGCTGGGCGAGGCTCCGTGGAGGCTGGATATGAGGTGGACAAAATTGCCCAGAGCCTGGATTTCAT
CAACCTTATGGCCTATGACTTTCATGGCTCTTGGGAGAGAGTCACAGGACATAACAGCCCTCTTTACAAG
GGGCAGGGAGAGAGCGGTGCAGAGGCTGAACTCAACGTGGACGCTGCTGTGCAACTGTGGCTGCAGAAAG
GGGCCCCTGCCAACAAACTGATCCTTGGCATGCCCACCTATGGACGATCCTTCACTCTGGCCTCCTCGTC
AGACACCAGAGTGGGGGCCCCAGCCACAGGGTCTGGTACCCCTGGCCCCTTCACCAAGGAGGGAGGAATT
CTGGCTTACTATGAGATCTGCTCCTGGAAAGGAGCCACTGAGCACAGAATCCAGGACCAGAAGGTGCCCT
ACCTCTTCCGGGACAACCAGTGGGTGGGCTTTGATGATGTGGAAAGCTTCAAAGCTAAGGTCAGTTACCT
GAAGCAGAAGGGACTGGGCGGGGCCATGGTCTGGACCCTGGACTTGGACGACTTCGCTGGCGTCTCCTGC
AGC
>Callithrix_jacchus
ATGGCAAAACTGGTCTGCTACTTCACCAACTGGGCCCAGTACAGACAGGGGGCGGCTCGCTTCCTGCCCA
AGGACGTGGACCCCAGCCTGTGCACCCACCTCATCTACGCCTTCGCCGGCATGACCAGTCACCAGCTGAG
CACAACTGAGTGGAATGACGAGACTCTCTACCAGGAGTTCAACGGCCTGAAGAAGATGAATCCCAAGCTG
AAGACCCTGTTGGCCATCGGAGGCTGGAATTTCGGCACTCAGAAGTTCACAGATATGGTAGCCACGGCCA
ACAACCGTCAGACCTTTGTCAACTCAGCCATCAGGTTTCTGCGCAAATACGGCTTTGACGGTCTTGACCT
TGACTGGGAGTACCCAGGGAGCCGGGGAAGCCCTGCCATAGACAAGGAGCGCTTCACAGCCCTGGTGCAG
GACTTGGCCAATGCCTTCCAGAAGGAAGCTAAGACCTCAGGGAAAGAACGCCTCCTTCTGAGTGCAGCGG
TTCCAGCTGGGAGAACCTATGTGGATGCTGGATACGAGGTGGACAAAATTGCCCAGAACCTGGACTTTGT
CAACCTTATGGCCTATGACTTCCATGGCTCTTGGGAGAAGGTCACGGGACACAACAGCCCCCTCTACAAG
AGGCAAGGGGAGAGTGGCGCAGCGGCCAGCCTCAACGTGGATGCTGCCGTGCAACTATGGCTGCAGAAGG
GGACCCCTGCCAGCAAACTGATCCTTGGCATGCCTACCTATGGGCGCTCCTTCACCCTGGCCTCCTTGTC
AGACACCAAAGTGGGGGCCCCAGCCACAGGGTCTGGTACCCCTGGCCCCTTCACCAAGGAAGGAGGGATG
CTGGCTTACTATGAGGTCTGCTCCTGGAAGGGGGCCACCAAGCAGAGAATCCAGGACCAGAAGGTGCCCT
ACATCTTCCGGGACAACCAGTGGGTGGGCTTTGATGATGCAGAGAGCTTCAAAGCCAAGGTCAGCTATAT
GAAGCAGAAAGGACTGGGCGGGGCCATGGTCTGGGCACTGGACTTAGATGACTTTGCCGGCTTCTCCTGC
AAC
>Trachypithecus_francoisi
ATGGCAAAACTGGTCTGCTACTTCACCAACTGGGCCCAGTACAGACAGGGGGAGGCTCGCTTCCTGCCCA
AGGACGTGGACCCCAGCCTTTGCACCCACCTCATCTACGCCTTCGCTGGCATGACCAACCACCAGCTGAG
CACCGTTGAGTGGAATGACGAGACTCTCTACCAGGAGTTCAATGGCCTGAAGAAGATGAATCCCAAGCTG
AAGACCCTGTTAGCCATCGGAGGCTGGAATTTCGGCACTCAGAGGTTCACAGATATGGTAGCCACGGCCA
ACAACCGTCAGACCTTCGTCAACTCAGCCATCAGGTTTCTGCGCAAATACGGCTTTGATGGCCTTGACCT
TGACTGGGAGTACCCAGGAAGCCGGGGAAGCCCTGCCATAGACAAGGAACGCTTCACAGCCCTGGTGCAG
GACTTGGCCAATGCCTTCCAGCAGGAAGCCCAGACCTCAGGGAAGGAATGCCTCCTTCTGAGTGCAGCGG
TTCCAGCTGGGCAGACCTATGTGGATGCTGGATATGAGGTGGACAAAATCACCAGGAACCTGGATTTTGT
CAACCTTATGGCCTATGACTTCCATGGCTCTTGGGAGAAGGTCACAGGACATAACAGCCCCCTCTACAAG
AGGCAAGAGGAGAGTGGTGCAGCGGCCAGCCTCAACGTGGATGCTGCTGTGCAACAGTGGCTGCAGAAGG
GGACCCCTGCCAGCAAGCTGATCCTTGGCATGCCTACCTATGGACGCTCCTTCACCCTGGCCTCCTCGTC
AGACACCAGAGTGGGGGCCCCAGCCACAGGGTCTGGCACTCCTGGCCCCTTCACCAAGGAAGGAGGGATG
TTGGCCTACTATGAGGTCTGCTCCTGGAAGGGGGCCACCAAACAGAGAATCCAGGACCAGGAGGTGCCCT
ACATCTTCCGGGACAACCAGTGGGTGGGCTTTGATGATGTGGAGAGCTTCAAAACCAAGGTCAGCTATCT
GAAGCAGAAGGGACTGGGCGGGGCGATGGTCTGGGCACTGGATTTAGATGACTTTGCCGGCTTCTCCTGC
GAC
>Mandrillus_leucophaeus
ATGGCAAAACTGGTCTGCTACTTCACCAACTGGGCCCAGTACAGACAGGGGGAGGCTCGCTTCATGCCCA
AGGACGTGGACCCCAGCCTTTGCACCCACCTCATCTACGCCTTCGCTGGCATGACCAACCACCAGCTGAG
CACCATTGAGTGGAATGACGAGACTCTCTACCAAGAGTTCAATGGCCTGAAGAAGATGAATCCCAAGCTG
AAGACCCTGTTAGCCATCGGAGGCTGGAATTTCGGCACTCAGAGGTTCACAGATATGGTAGCCACGGCCA
ACAACCGTCAGACCTTCGTCAACTCAGCCATCAGATTTCTGCGCAAATACGGCTTTGATGGCCTTGACCT
TGACTGGGAGTACCCAGGAAGCCGGGGGAGCCCTGCCATAGACAAGGAACGCTTCACAGCCCTGGTGCAG
GACTTGGCCAACGCCTTCCAGCAGGAAGCCCAGACCTCAGGGAAGGAACGCCTCCTTCTGAGTGCAGCGG
TTCCAGCTGGGCGGACCTATGTGGATGCTGGATACGAGGTGGACAAAATCGCCAGGAACCTGGATTTTGT
CAACCTTATGGCCTACGACTTCCATGGCTCTTGGGAGAAGGTCACAGGACATAATAGCCCCCTCTACAAG
AGGCAAGAGGAGAGTGGTGCAGCGGCCAGCCTCAACGTGGATGCTGCTGTGCAAATGTGGCTGCAGAAGG
GGACCCCTGCCAGCAAGCTGATCCTTGGCATGCCTACCTACGGACGCTCCTTCACCCTGGCCTCCCCGTC
AGACACCAGAGTGGGGGCCCCAGCCACAGGGTCTGGCACTCCTGGCCCCTTCACCAAGGAAGGAGGGATA
TTGGCCTACTATGAGGTCTGCTCCTGGAAGGGGGCCACCAAACAGAGAATCCAGGACCAGGAGGTGCCCT
ACATCTTCCGGGACAACCAGTGGGTGGGCTTTGATGATGTGGAGAGCTTCAAAACCAAGGTCAGCTATCT
GAAGCAGAAGGGACTGGGCGGGGCGATGGTCTGGGCACTGGATTTAGATGACTTTGCCGGCTTCTCCTGC
GAC

>Pan_paniscus
ATGGCAAAACTGGTCTGCTACTTCACCAACTGGGCCCAGTACAGACAGGGGGAGGCTCGCTTCCTGCCCA
AGGACGTGGACCCCAGCCTTTGCACCCACCTCATCTACGCCTTCGCTGGCATGACCAACCACCAGCTAAG
CACCATTGAGTGGAATGACGAGACTCTCTACCAGGAGTTCAATGGCCTGAAGAAGATGAATCCCAAGCTG
AAGACCCTGTTAGCCATCGGAGGCTGGAATTTCGGCACTCAGAAGTTCACAGATATGGTAGCCACGGCCA
ACAACCGTCAGACCTTTGTCAACTCGGCCATCAGGTTTCTGCGCAAATACAGCTTTGACGGCCTTGACCT
TGACTGGGAGTACCCAGGAAGCCGGGGGAGCCCTGCCGTAGACAAGGAGCGCTTCACAACCCTGGTACAG
GACTTGGCCAATGCCTTCCAGCAGGAAGCCCAGACCTCAGGGAAGGAACGCCTTCTTCTGAGTGCAGCGG
TTCCAGCTGGGCAGACCTATGTGGATGCTGGATACGAGGTGGACAAAATCACCCAGAATCTGGATTTTGT
CAACCTTATGGCCTACGACTTCCATGGCTCTTGGGAGAAGGTCACGGGACATAACAGCCCCCTCTACAAG
AGGCAAGAGGAGAGTGGTGCAGCAGCCAACCTCAACGTGGATGCTGCTGTGCAACAGTGGCTGCAGAAGG
GGACCCCGGCCAGCAAGCTGATCCTTGGCATGCCTACCTACGGACGCTCCTTCACACTGGCCTCCTCATC
AGACACCAGAGTGGGGGCCCCAGCCACAGGGTCTGGCACTCCCGGCCCCTTCACCAAGGAAGGAGGGATG
CTGGCCTACTATGAGGTCTGCTCCTGGAAGGGGGCCACCAAACAGAGAATCCAGGATCAGAAGGTGCCCT
ACATCTTCCGGGACAACCAGTGGGTGGGCTTTGATGATGTGGAGAGCTTCAAAACCAAGGTCAGCTATCT
GAAGCAGAAGGGACTGGGCGGGGCCATGGTCTGGGCACTGGACTTAGATGACTTTGCCGGCTTCTCCTGC
AAC
>Nomascus_leucogenys
ATGGCAAAACTGGTCTGCTACTTCACCAACTGGGCCCAGTACAGACAGGGGGAGGCTCGCTTCCTGCCCA
AGGACGTGGACCCCAGCCTTTGCACCCACCTCATCTACGCCTTCGCTGGCATGACCAACCACCAGCTGAG
CACCATTGAGTGGAATGACGAGACTCTGTACCAGGAGTTCAATGGCCTGAAGAAGATGAATCCCAAGCTG
AAGACCCTGTTAGCCATCGGAGGCTGGAATTTCGGCACTCAGAAGTTCACAGATATGGTAGCCACGGCCA
ACAACCGTCAGACCTTTGTCAACTCAGCCATCAGGTTTCTGCGGAAATACAGCTTTGACGGCCTTGACCT
TGACTGGGAGTACCCAGGAAGCCGGGGGAGCCCTGCCGTAGACAAGGAGCGCTTCACAGCCCTGGTACAG
GACTTGGCCAATGCCTTCCAGCAGGAAGCCCAGACCTCAGGGAAGGAACGCCTTCTTCTGAGTGCAGCGG
TTCCAGCTGGGCGGACCTATGTGGATGCTGGATACGAAGTGGACAAAATCACCCAGAACCTGGATTTTGT
CAGCCTTATGGCCTACGACTTCCATGGCTCTTGGGAGAAGGTCACGGGACATAACAGCCCCCTCTACAAG
AGGCAAGAGGAGAGTGGTGCAGCAGCCAGCCTCAATGTGGATGCTGCTGTGCAACTGTGGCTGCAGAAGG
GGACCCCTGCCAGCAAGCTGATTCTTGGCATGCCTACCTACGGACGCTCCTTCACCCTGGCCTCCTCATC
AGACACCAGAGTGGGGGCCCCAGCCACAGGGTCTGGCACTCCCGGCCCCTTCACCAAGGAAGGAGGCATG
CTGGCCTACTATGAGGTCTGCTCCTGGAAGGGGGCCACCAAACAGAGAATCAAGGACCAGGAGGTGCCCT
ACATCTTCCGGGACAACCAGTGGGTGGGCTTTGATGATGTGGAGAGCTTCAAAACCAAGGTCAGCTATCT
GAAGCAGAAGGGACTGGGCGGGGCCATGGTCTGGGCACTGGACTTAGACGACTTTGCCAGCTTCTCCTGC
AAC
>Gorilla_gorilla
ATGGCAAAACTGGTCTGCTACTTCACCAACTGGGCCCAGTACAGACAGGGAGAGGCTCGCTTCCTGCCCA
AGGACGTGGACCCCAGCCTTTGCACCCACCTCATCTACGCCTTCGCTGGCATGACCAACCACCAGCTGAG
CACCATTGAGTGGAATGACGAGACTCTCTACCAGGAGTTCAATGGCCTGAAGAAGATGAATCCCAAGCTG
AAGACCCTGTTAGCCATCGGAGGCTGGAATTTCGGCACTCAGAAGTTCACAGATATGGTAGCCACGGCCA
ACAACCGTCAGACCTTTGTCAACTCGGCCATCAGGTTTCTGCGCAAATACAGCTTTGACGGGCTTGACCT
TGACTGGGAGTACCCAGGAAGCCGGGGGAGCCCTGCCGTAGACAAGGAGCGCTTCACAGCCCTGGTACAG
GACTTGGCCAATGCCTTCCAGCAGGAAGCCCAGACCTCAGGGAAGGAACGCCTTCTTCTGAGTGCAGCGG
TTCCAGCTGGGCAGACCTATGTGGATGCTGGATACGAGGTGGACAAAATCGCCCAGAACCTGGATTTTGT
CAACCTTATGGCCTACGACTTCCATGGCTCTTGGGAGAAGGTCACGGGACATAACAGCCCCCTCTACAAG
AGGCAAGAGGAGAGTGGTGCAGCAGCCAGCCTCAACGTGGATGCTGCTGTGCAACAGTGGCTGCAGAAGG
GGACCCCTGCCAGCAAGCTGATCCTTGGCATGCCTACCTACGGACGCTCCTTCACCCTGGCCTCCTCATC
AGACACCAGAGTGGGGGCCCCAGCCACAGGGTCTGGCACTCCCGGCCCCTTCACCAAGGAAGGAGGGATG
CTGGCCTACTATGAGGTCTGCTCCTGGAAGGGGGCCACCAAACAGAGAATCCAGGACCAGAAGGTGCCCT
ACATCTTCCGGGACAACCAGTGGGTGGGCTTTGATGATGTGGAGAGCTTCAAAACCAAGGTCAGCTATCT
GAAGCAGAAGGGACTGGGCGGGGCCATGGTCTGGGCACTGGACTTAGATGACTTTGCCGGCTTCTCCTGC
AAC
>Pan_troglodytes
ATGGCAAAACTGGTCTGCTACTTCACCAACTGGGCCCAGTACAGACAGGGGGAGGCTCGCTTCCTGCCCA
AGGACGTGGACCCCAGCCTTTGCACCCACCTCATCTATGCCTTCGCTGGCATGACCAACCACCAGCTAAG
CACCATTGAGTGGAATGACGAGACTCTCTACCAGGAGTTCAATGGCCTGAAGAAGATGAATCCCAAGCTG
AAGACCCTGTTAGCCATCGGAGGCTGGAATTTCGGCACTCAGAAGTTCACAGATATGGTAGCCACGGCCA
ACAACCGTCAGACCTTTGTCAACTCGGCCATCAGGTTTCTGCGCAAATACAGCTTTGACGGCCTTGACCT
TGACTGGGAGTACCCAGGAAGCCGGGGGAGCCCTGCCGTAGACAAGGAGCGCTTCACAGCCCTGGTACAG
GACTTGGCCAATGCCTTCCAGCAGGAAGCCCAGACCTCAGGGAAGGAACGCCTTCTTCTGAGTGCAGCGG
TTCCAGCTGGGCAGACCTATGTGGATGCTGGATACGAGGTGGACAAAATCGCCCAGAACCTGGATTTTGT
CAACCTTATGGCCTACGACTTCCATGGCTCTTGGGAGAAGGTCACGGGACATAACAGCCCCCTCTACAAG
AGGCAAGAGGAGAGTGGTGCAGCAGCCAGCCTCAACGTGGATGCTGCTGTGCAACAGTGGCTGCAGAAGG
GGACCCCGGCCAGCAAGCTGATCCTTGGCATGCCTACCTACGGACGCTCCTTCACACTGGCCTCCTCATC
AGACACCAGAGTGGGGGCCCCAGCCACAGGGTCTGGCACTCCCGGCCCCTTCACCAAGGAAGGAGGGATG
CTGGCCTACTATGAGGTCTGCTCCTGGAAGGGGGCCACCAAACAGAGAATCCAGGATCAGAAGGTGCCCT

```
ACATCTTCCGGGACAACCAGTGGGTGGGCTTTGATGATGTGGAGAGCTTCAAAACCAAGGTCAGCTATCT
GAAGCAGAAGGGACTGGGCGGGGCCATGGTCTGGGCACTGGACTTAGATGACTTTGCCGGCTTCTCCTGC
AAC
>Saimiri_boliviensis
ATGGCGAAACTGGTCTGCTACTTCACCAACTGGGCCCAGTACAGACAGGGGGCGGCTCGCTTCCTGCCCA
AGGACGTGGACCCCGGCCTGTGCACCCATCTCATCTACGCCTTCGCCGGCATGACCAGTCACCAGCTGAG
CACCATTGAGTGGAATGACGAGACTCTCTACCAGGAGTTCAACAGCCTGAAGAAGATGAATCCCAACCTG
AAGACCCTGTTGGCCATCGGAGGCTGGAATTTTGGCACTCAGAAGTTCACAGATATGGTAGCTACGGCCA
ACAACCGTCAGACCTTTGTCAACTCAGCCATCAGGTTTCTGCGCAAATACGGCTTTGATGGCCTTGACCT
TGACTGGGAGTACCCAGGGAGCCGGGGGAGCCCTGCCGTAGACAAGGAGCGCTTCACAGCCCTGGTGCAG
GACTTGGCCAACGCCTTCCAGAAGGAAGCTCAGACCTCAGGGAAGGCACGTCTCCTTCTGAGTGCGGCGG
TTCCAGCTGGACGAACCTATGTGGATGCTGGATACGAGGTGGACAAAATTGCCCAGACCCTGGACTTTGT
CAACCTGATGGCCTATGACTTCCATGGCTCTTGGGAGAAGGTCACGGGACACAACAGCCCCCTCTACAAG
AGGCAAGGGGAGAGTGGTGCAGCGGCCAGCCTCAACGTGGATGCTGCTGTGCAACTGTGGCTGCAGAAGG
GGACCCCTGCCAGCAAGCTGATCCTTGGCATGCCTACCTACGGACGCTCCTTCACCTTGGCCTCCTCATC
AGACACCAGAGTGGGGGCCCCAGCCACAGGGTCTGGTACCCCTGGCCCACTCACCAAGGAAGGAGGGATG
CTGGCCTACTATGAGGTCTGCTCCTGGAAGGGGGCCACCAAGCAGAGAATCCAGGACCAGAAGGTGCCCT
ACATCTTCCGGGACAACCAGTGGGTGGGCTTTGATGATGCGGAGAGCTTCAAAGCCAAGGTCAGCTATCT
GAAGCAGAAAGGACTGGGCGGGGCCATGGTCTGGGCACTGGACTTAGATGACTTTGCCGGCTTCTCCTGC
AAC
>Piliocolobus_tephrosceles
ATGGCAAAACTGGTCTGCTACTTCACCAACTGGGCCCAGTACAGACAGGGGGAGGCTCGCTTCCTGCCCA
AGGACGTGGACCCCAGCCTTTGCACCCACCTCATCTACGCCTTCGCTGGCATGACCAACCACCAGCTGAG
CACCATTGAGTGGAATGATGAGACTCTCTACCAGGAGTTCAATGGCCTGAAGAAGATGAATCCCAAGCTG
AAGACCCTGTTAGCCATCGGAGGCTGGAATTTCGGCACTCAGAAGTTCACAGATATGGTAGCCACGGCCA
ACAACCGTCAGACCTTCGTCAACTCAGCCATCAGGTTTCTGCGCAAATACAGCTTTGATGGCCTTGACCT
TGACTGGGAGTACCCAGGAAGCCGGGGGAGCCCTGCCATAGACAAGGAACGCTTCACGGCCCTGGTGCAG
GACTTGGCCAACGCCTTCCAGCAGGAAGCCCAGACCTCAGGGAAGGAACGCCTCCTTCTGAGTGCAGCGG
TTCCAGCTGGGCAGACCTATGTGGATGCTGGATATGAGGTGGACAAAATCGCCAGGAACCTGGATTTTGT
CAACCTTATGGCCTATGACTTCCATGGCTCTTGGGAGAAGGTCACGGGACATAATAGCCCCCTCTACAAG
AGGCAAGAGGAGAGTGGTGCAGCGGCCAGCCTCAACGTGGATGCTGCTGTGCAACAGTGGCTGCAGAAGG
GGACCCCTGCCAGCAAGCTGATCCTTGGCATGCCTACCTATGGACGCTCCTTCACCCTGGCCTCCTCGTT
AGACACCAGAGTGGGGGCCCCAGCCACAGGGTCTGGCACTCCTGGCCCCTTCACCAAGGAAGGAGGGATG
TTGGCCTACTATGAGGTCTGCTCCTGGAAGGGGGCCACCAAACAGAGAATCCAGGACCAGGAGGTGCCCT
ACATCTTCAGGGACAACCAGTGGGTGGGCTTTGATGATGTGGAGAGCTTCAAAACCAAGGTCAGCTATCT
GAAGCAGAAGGGACTGGGCGGGGCGATGGTCTGGGCACTGGATTTAGATGACTTTGCCGGCTTCTCCTGC
GAC
>Sapajus_apella
ATGGCAAAACTGGTCTGCTACTTCACCAACTGGGCCCAGTACAGACAGGGGGCGGCTCGCTTCCTGCCCA
AGGACGTGGACCCCAGCCTGTGCACCCACCTCATCTACGCCTTCGCTGGCATGACCAATCACCAGCTGAG
CACCACTGAGTGGAATGACGAGACTCTCTACCAGGAGTTCAACAGCCTGAAGAAGATGAATCCCAAGCTG
AAGACCCTGTTGGCCATCGGAGGCTGGAATTTCGGCACTCAGAAGTTCACAGATATGGTAGCCACGGCCA
ACAACCGTCAGACCTTCGTCAACTCAGCCATCAGGTTTCTGCGCAAATACGGCTTTGACGGCCTTGACCT
TGACTGGGAGTACCCAGGGAGCCGGGGGAGCCCTGCCATAGACAAGGAGCGCTTCACAGCCCTGGTGCAG
GACTTGGCCAACGCCTTCCAGAAGGAAGCTCAGACCTCAGGGAAGGAACGCCTCCTTCTGAGTGCAGCGG
TTCCAGCTGGGCGAACCTATGTGGATACTGGATACGAGGTGGACAAAATTGCCCAGAACCTGGATTTTGT
CAACCTTATGGCCTATGACTTCCATGGCTCTTGGGAGAAGGTCACGGGACACAACAGCCCCCTCTACAAG
AGGCAAGGGGAGAGTGGTGCAGCGGCCAGCCTCAACGTGGATGCTGCTGTGCAGCTGTGGCTGCAGAAGG
GGACACCTGCCAGCAAGCTGATCCTTGGCATGCCTACCTACGGACGCTCCTTCACCCTGGCCTCCTCGTC
AGACACCAGAGTGGGGGCCCCAGCCACAGGGTCTGGTGCCCCTGGCCCCTTCACCAAGGAAGGAGGGATG
CTGGCCTACTATGAGGTCTGCTCCTGGAAGGGGGCCACCAAGCAGAGAATCCAGGACCAGAAGGTGCCCT
ACATCTTCCGGGACAAGCAGTGGGTGGGCTTTGATGATGCAGAGAGCTTCAAAGCCAAGGTCAGCTATCT
GAAGCAGAAAGGACTGGGCGGGGCCATGGTCTGGGCGCTGGACTTAGATGACTTTGCCGGCTTCTCCTGC
AAC
>Pongo_abelii
ATGGCAAAACTGGTCTGCTACTTCACCAACTGGGCCCAATACAGACAGGGGGAGGCTCGCTTCCTGCCCA
AGGACGTGGACCCCAGTCTTTGCACCCACCTCATCTATGCCTTCGCTGGCATGACCAACCATCAGCTGAG
CACCGTTGAGTGGAATGACGAGACTCTCTACCAGGAGTTCAATGGCCTGAAGAAGATGAATCCCAAGCTG
AAGACCCTGTTAGCCATCGGAGGCTGGAATTTCGGCACTCAGAAGTTCACAGATATGGTAGCCACAGCCA
AAAACCGTCAGACGTTTGTCAACTCGGCCATCAGGTTTCTGCGCAAATACGGCTTTGACGGCCTTGACCT
TGACTGGGAGTACCCTGGAAGCCGGGGGAGCCCTGCCGTAGACAAGGAGCGCTTCACAGCCCTGGTACAG
GACTTGGCCAATGCCTTCCAGCAGGAAGCCCAGACCTCAGGGAAAGAAGGCCTTCTTCTGAGTGCAGCGG
TTCCAGCTGGGCGGACCTATGTGGATGCTGGATACGAGGTGGACAAAATCGCCCAGAACCTGGATTTTGT
CAACCTTATGGCCTACGACTTCCATGGCTCTTGGGAGAAGGTCACGGGACATAACAGCCCCCTCTACAAG
AGGCAAGAGGAGAGTGGTGCAGCAGCCAGCCTCAACGTGGATGCTGCTGTGCAACTGTGGCTGCAGAAGG
```

GGACCCCTGCCAGCAAGCTGATCCTTGGCATGCCTACCTACGGACGCTCCTTCACCCTGGCCTCCTCATC
AGACACCAGAGTGGGGGCCCCAGCCACAGGGTCTGGCACTCCCGGCCCCTTCACCAAGGAAGGAGGGATG
CTGGCCTACTATGAGGTCTGCTCCTGGAAGGGGGCCACCAAACAGAGAATCCAGGACCAGGAGGTGCCCT
ACATCTTCCGGGACAACCAGTGGGTGGGCTTTGATGATGTGGAGAGCTTCAAAACCAAGGTCAGCTATCT
GAAGCAGAAGGGACTGGGCGGGGCCATGGTCTGGGCACTGGACTTAGATGACTTTGCCAGCTTCTCCTGC
AAC
>Galeopterus_variegatus
ATGATAAAACTAGTCTGCTACTTCTCCAACTGGGCCCAGTACAGAGAGGGGGTGGCTCGCTTCTTGCCCA
GGGATGTGGACGCCAGCCTGTGCACCCACCTCATCTATGCCTTTGCCGGCATGAGCCATCACCAGCTCAG
CTCCGTGGAGTGGAACGACGAGGCTCTCTACCAGGAGTTCAACGGCCTGAAGCAGATGAATCCCAAGCTG
AAGACTCTGTTAGCCATTGGGGGCTGGAACTTCGGCACTCAGAAGTTCACAGATATGGTGGCCACGGCCA
ACAACCGTCAGACCTTTGTCAACTCGGCCATCAAGCTTCTGCGCAAATACAGCTTTGATGGCCTTGACCT
TGACTGGGAGTACCCAGGTAGCCGGGGGAGCCCCGCTGTGGACAAGGAGCGCTTCACAGCCTTGGTGCAG
GACTTGGCCAGAGCCTTCCAGCAGGAAGCCCAGACCTCAGGGAAGGAACGACTCCTTCTGAGCGCTGCAG
TCCCAGCTGGGCGAGTCCACGTGGAGGCTGGATATGAGGTGGACAGAATAGCTCAGAACTTGGATTTCAT
CAACCTCATGGCCTATGACTTCCACGGTTCGTGGGAGAGAGTCACAGGACATAACAGTCCCCTCTACAAG
AGGCAGGGAGAGAGTGGCGCAGCGGCCGAACTCAACGTGGATGCTGCTGTGCAGCAGTGGCTGCAGAAGG
GGGCCCCTGCCAACAAACTGATCCTTGGCATGCCCACCTATGGACGGACCTTCACCCTGGCCTCCTCGTC
AGACACCAGGGTAGGGGCACCAGCCACAGGGCCTGGCATCCGCGGCCCCTTCACCAAGGAGGCAGGGGTG
CTGGCTTACTATGAGGTCTGCTCCTGGAAGGAGGTCAGCAAGCACAGAATCAAGGACCAGGAGGTTCCCT
ACGCCTTCCAGAACAACCAGTGGGTGGGCTTCGATGATGCGGAGAGCTTTAAAGCCAAGGTCAGCTATCT
GAAGCAGAAGGGCCTGGGCGGGGCCATGGTCTGGACGCTGGACTTGGACGACTTTGCTGGCTTCTTCTGC
GGC
>Mus_musculus
ATGGCAAAACTGGTCTGCTACCTCACCAACTGGTCCCAGTACCGGACGGAGGCAGTTCGGTTCTTTCCCA
GGGATGTGGATCCCAACCTGTGTACCCACGTCATCTTTGCTTTTGCTGGAATGGACAACCATCAGCTCAG
CACTGTGGAGCACAATGACGAACTTCTCTACCAGGAGCTGAACAGCCTAAAGACTAAGAACCCCAAGCTC
AAGACCCTGTTAGCCGTTGGAGGCTGGACCTTTGGTACCCAGAAGTTCACAGACATGGTGGCCACCGCCA
GCAACCGGCAGACCTTTGTGAAGTCAGCCCTAAGTTTCCTGCGCACTCAAGGTTTTGATGGCCTTGACCT
TGACTGGGAGTTCCCAGGTGGACGTGGGAGCCCCACAGTAGACAAAGAGAGATTCACAGCCCTGATACAG
GACTTGGCCAAAGCCTTCCAGGAGGAAGCCCAGTCCTCAGGGAAGGAACGCCTCCTTCTGACTGCAGCTG
TACCGAGTGATCGAGGCCTGGTGGATGCTGGCTACGAGGTGGACAAGATTGCCCAGAGCTTGGATTTCAT
CAACCTTATGGCCTACGACTTCCACAGCTCCTTGGAAAAGACCACAGGGCATAACAGCCCCCTCTACAAA
AGGCAAGGAGAAAGTGGGGCAGCCGCTGAGCAAAACGTGGATGCTGCTGTGACGCTCTGGCTGCAGAAGG
GGACCCCAGCCAGCAAACTGATCCTTGGCATGCCTACCTATGGACGCTCTTTCACCTTGGCCTCCTCGTC
AGACAATGGAGTTGGGGCCCCAGCCACAGGGCCTGGTGCCCCAGGCCCCTATACGAAGGACAAAGGGGTC
CTGGCTTACTATGAGGCCTGCTCCTGGAAGGAAAGACACAGAATCGAGGACCAGAAGGTGCCTTACGCCT
TCCAGGACAACCAGTGGGTGAGCTTTGACGACGTGGAAAGCTTCAAAGCCAAGGCTGCCTACCTGAAACA
GAAGGGGCTGGGAGGAGCCATGGTCTGGGTCCTGGACTTGGATGACTTCAAGGGTTCCTTCTGCAAC
>Rattus_norvegicus
ATGGCAAAACTGTTCTGCTACTTCACCAACTGGGCCCAGTACCGGTCCGGGGCAGCTCGATTCCTACCTA
GGGATGTGGATCCCAACCTGTGTACCCATGTCATCTATGCCTTTGCTGGACTGAACAACCACCAGGTCAG
CACTGTAGAGCCCAATGACGAGCTTTTCTACCAAGAGCTGAACAGCCTAAAGAAGAGGAACCCCAAGCTC
AAGACCCTGTTAGCCGTCGGGGGCTGGAGCTTTGGTACCCAGAAGTTCACAGACATGGTGGCCACAGCCA
GCACCCGGCAGACCTTTGTCAACTCAGCTCTCTCGTTCCTGCGCACTCATGGTTTTGACGGCCTTGACCT
TGACTGGGAATACCCAGGAAGCCGAGGGAGCCCAGCAGTAGACAAAGAGAGATTCACAGCGCTGATACAG
GATTTGGCCAAAGCCTTCCAGGAGGAAGCCCGGGCCTCAGGGAAAAGTCGCCTCCTTCTGACTGCAGCTG
TACCAACTGGTCGAGGCCATGTGGATGCTGGTTATGAGGTGGACAAGATTGTTCAGAGTTTGGATTTCAT
CAACCTTATGGCCTACGACTTCCACAGCTCCTGGGACAAGACCACAGGGCACAACAGCCCCCTCTACAAA
AGGCAAGGAGAGACTGGGAAAGATGCTGAAAAAAATGTGGATGCTGCTGTGACGCTCTGGCTGCAGAAGG
GGACCCCTGCCAGCAAACTGATGCTTGGCATGCCCGCCTACGGACGCTCCTTCACCTTGGCCTCCTCATC
AGACAGTGGAGTTGGGGCTCCAGCCACAGGACCTGGGGCCCCAGGCCCCTATACTAAGGAAAAGGGGATA
CTGGTTTACTTTGAGGTCTGCTCCTGGAAGGGAAAACAGAGAATCGAGGACCAGAAGGTGCCCTATGTCT
CCCAGGGCAACCAATGGGTGGGATTTGATGACAGGGAAAGCTTCAGAGCCAAGGCTGCCTATGTGAAACA
GAAGGGACTGGGAGGGGCCATGGTCTGGATTCTGGATGGGGATGACTTCAAAGGTTCCTTCTGCAAC
>Sus_scrofa
ATGGCAAAACTGGTCTGCTACTTCACCAACTGGGCCCAGTACAGAGAGGGGGCTGCTCGCTTCTTGCCCA
AGGATGTGGACCCCAATCTGTGCACCCATCTCATCTATGCCTTTGCTGGCATGAACAACCACCAGCTCAG
CTCCACAGAGTGGAATGATGAGGCGCTATACAAAGACTTCAATGGCCTGAAGAAGATGAATCCCAAGCTG
AAGACACTGCTGGCCATTGGGGGCTGGAACTTCGGCACTCAGAAGTTCACGGACATGGTGGCTACAGTCA
ACAACCGACACACCTTTGTCAACTCAGCCATCAGGTTTCTGCGCAAATATGGCTTTGATGGCCTTGACCT
TGACTGGGAGTACCCAGGAAGCCGAGTAAGTCCCCCTTCAGACAAGCAGCGCTTCACAGCCCTGGTGCAG
GACCTGGCCAATGCCTTTAAGCAGGAAGCCCAGATCACAGGGAAGGCACGCCTCCTACTGAGCGCAGCTG
TCTCAGCAGCACGACCCAGTATAGAAGCTGGATATGAGGTGGATCAAATTGCTCCGAACCTGGACTTCCT
CAGCCTCATGGTCTATGACTTCCATGGCTTGTGGGAGAAGACCACCGGGCATCATAGCCCCCTCTACAGG

AGGCAGGGAGAGAGCGGGGCAGCAGCTGAATTCAATGTGGACTCTGCTGTGCAACAGTGGCTGCAGAAAG
GGACTCCCGCCAACAAACTGATCCTTGGTATGCCCACCTATGGACGGTCCTTTACCCTGGCCTCCTCACT
GGACACTGGGGTAAGGGTCCCAGCCACAGGGCCTGGAACTCCTGGCCCCTTCACCAAAGAGGGAGGACTG
CTGGCCTACTTTGAGGTCTGCTCCTGGAAGGGGGCTTCTGAGCACAGAATCAAGGAACAGAAGGTGCCCT
ATGCCTTCCAGGGCAACCAGTGGGTGGGTTTTGATGACACGGAGAGCTTCAAAACCAAGGTGAGGTGCCT
GAAGCAGAAGAGACTGGGCGGGGCCATGGTCTGGACACTGGATATGGATGACTTTGCTGGCTCCTTCTGT
AAC
